# Fundamental limits on symmetry breaking by Turing-like activator-inhibitor mechanisms

**DOI:** 10.1242/dev.205067

**Published:** 2026-03-23

**Authors:** Daniel Muzatko, Bijoy Daga, Tom W. Hiscock

**Affiliations:** Institute of Medical Sciences, University of Aberdeen, Aberdeen AB25 2ZD, UK

**Keywords:** Self-organisation, Turing patterns, Mathematical models, Embryonic patterning, Developmental signalling, Symmetry breaking

## Abstract

Turing's longstanding reaction-diffusion hypothesis explains how molecular patterns can self-organise *de novo* in otherwise homogeneous tissues. However, whilst Turing-like activator-inhibitor models can qualitatively recapitulate patterning *in silico*, they are often highly simplified approximations of the molecular complexity operating *in vivo.* Here, we investigate significantly more complex reaction-diffusion systems that seek to more directly capture the mechanisms involved in intercellular signalling. By combining large-scale simulations with formal mathematical proofs, we show, rather generally, that symmetry breaking is strongly constrained by the extracellular interactions in the system but is relatively insensitive to the intracellular dynamics assumed. When applied to the activator-inhibitor paradigm, we find a broader repertoire of self-organising circuits than previously recognised, including some which are unexpectedly robust to parameters. Beyond these examples, we have packaged our highly performant numerical methods into a freely available and easy-to-use software pipeline, ReactionDiffusion.jl, that allows arbitrarily complex reaction-diffusion systems to be simulated at scale.

## Teaser

**Summary:** *In silico* analysis of complex reaction-diffusion mechanisms reveals fundamental limits on the ability of activator-inhibitor systems to break symmetry *in vivo*.

## INTRODUCTION

Symmetry breaking is one of the most fundamental processes in developmental biology, allowing otherwise homogeneous tissues/embryos to self-organise into complex patterns and structures. The ability of early embryos to form patterns *de novo* has long been recognised, dating back to some of the earliest experiments in embryology in the late 1800s (Driesch, 1892; Roux, 1888). Beyond early embryos, self-organisation also occurs in many developing tissues, as evidenced by the ability of diverse organoid systems to self-organise patterns *in vitro* (Eiraku et al., 2011; Ishihara and Tanaka, 2018; Meinhardt et al., 2014; Merle et al., 2024; Simunovic et al., 2019)*.* Some of the most well-studied paradigms of self-organisation involve periodic (i.e. repeating) patterns (Hiscock and Megason, 2015; Kondo and Miura, 2010; Marcon and Sharpe, 2012; Raspopovic et al., 2014; Sick et al., 2006), in which initially uniform tissues spontaneously break symmetry to generate identical/similar structures at regular spatial intervals (e.g. teeth, hair, leaves).

**Fig. 1.**
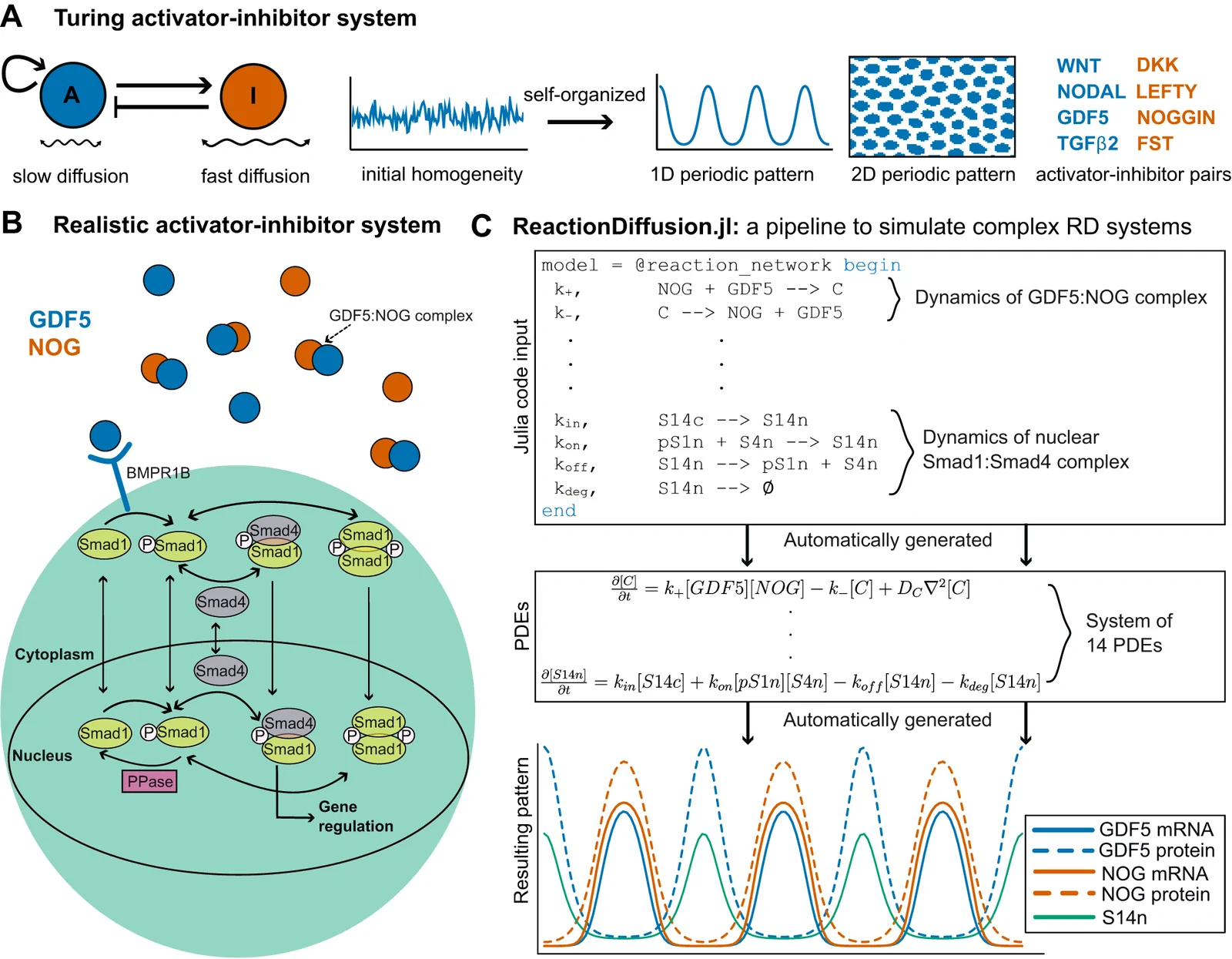
**Self-organisation in activator-inhibitor systems.** (A) Simplified reaction-diffusion models that comprise two diffusible molecules – an activator and its own inhibitor – can self-organise repeated patterns *in silico* (Gierer and Meinhardt, 1972; Turing, 1952). (B) Schematic of complex intercellular interactions involved in an example activator-inhibitor system *in vivo* (Grall et al., 2024; Schmierer et al., 2008). (C) Outline of ReactionDiffusion.jl, a computational pipeline to efficiently simulate complex reaction-diffusion systems. The model shown corresponds to the interactions schematised in B. Model assumptions are written in an intuitive syntax that reflects biochemical interactions. Models are automatically converted to partial differential equations and then automatically simulated until reaching a stationary pattern.

Decades of theoretical work – initiated by Alan Turing in the 1950s (Turing, 1952) – has advanced the hypothesis that these self-organising patterns are the result of reacting and diffusing chemical signals. *In vivo*, these reaction-diffusion systems can be implemented by intercellular feedback circuits of secreted signalling molecules that diffuse in the extracellular space. In the canonical Turing model (as well as subsequent Gierer-Meinhardt models; Gierer and Meinhardt, 1972; Meinhardt and Gierer, 1974) there are two such diffusible molecules – an activator and its (more rapidly diffusing) inhibitor (Fig. 1A). These activator-inhibitor systems can self-organise *in silico* from a homogeneous initial state into a diversity of eventual patterns, often periodic patterns such as dots or stripes. Moreover, putative activator/inhibitor pairs have been proposed for a variety of tissues and signalling pathways, and *in silico* Turing models can be made to qualitatively recapitulate patterns formed *in vivo* (Grall et al., 2024; Kondo and Asai, 1995; Raspopovic et al., 2014; Sick et al., 2006).

However, Turing-like activator-inhibitor models are often highly simplified, approximating complex mechanisms by a handful of equations that loosely mirror the regulatory interactions present *in vivo*, and neglect the essential roles that cellular and/or mechanical interactions may be playing (Hiscock and Megason, 2015; Ho et al., 2019; Nakamasu et al., 2009; Shyer et al., 2017; Singh et al., 2014). Moreover, when modelling pure reaction-diffusion systems *in silico*, they can be highly sensitive to parameters and often only form patterns in a narrow and biophysically unrealistic region of parameter space (Maini et al., 2012; Scholes et al., 2019), although more complex circuits have been proposed for which this parameter space is larger (Marcon et al., 2016; Scholes et al., 2019; Woolley, 2025a; Woolley et al., 2021). However, it remains unclear whether simple activator-inhibitor mechanisms alone are capable of robust patterning *in vivo*, casting doubt on the relevance of Turing's original hypothesis.

Despite this, recent experimental data are beginning to pinpoint specific patterns that are generated by definitively molecular (i.e. reaction-diffusion) mechanisms. The clearest examples draw from tissues that undergo minimal morphogenesis during initial patterning, such as the developing hair follicles (Sick et al., 2006) and fingerprints in the mammalian epidermis (Glover et al., 2023), or the patterning of joints within the digits (Grall et al., 2024). In these cases, a self-organised molecular prepattern is the first sign of symmetry breaking and precedes any overt changes in cell density or tissue morphology, strongly implicating a reaction-diffusion mechanism.

At the same time, theoreticians have investigated reaction-diffusion systems that are considerably more complex than simple activator-inhibitor schemes e.g. by incorporating more than two diffusible molecules. High throughput, systematic analyses of three- and four-node networks have revealed a wide array of regulatory architectures capable of periodic patterning beyond simple two-component Turing models (Diego et al., 2018; Haas and Goldstein, 2021; Marcon et al., 2016; Scholes et al., 2019; Shaberi et al., 2025; Zheng et al., 2016), suggesting that symmetry breaking may involve more than just one activator and one inhibitor *in vivo*. These analyses reveal that many types of periodic pattern can form with a variety of phase relationships possible amongst the different model components. Moreover, a general theme that has emerged is that factors that do not diffuse between cells (e.g. transcription factors, signal transduction elements) can significantly impact the ability of the system to break symmetry (Diego et al., 2018; Marcon et al., 2016; Scholes et al., 2019). However, given the large number of these components and their possible interactions, previous models have necessarily underestimated the intracellular complexity present *in vivo*, and it remains unclear what impact this simplification will have on model predictions. Moreover, whilst containing more than two elements, the models are still simplified network-level abstractions which approximate molecular interactions by simple mathematical expressions (e.g. linear or hill functions), rather than directly capturing the nature of developmental signalling *in vivo*. One strategy has been to assume sigmoidal/hill-type interactions between each component (Scholes et al., 2019; Zheng et al., 2016); this has the advantage of naturally capturing the saturating nature of many biological feedbacks, but poorly reflects more direct biochemical interactions (for example, binding of an inhibitor to an activator molecule in the extracellular space). Another approach is to instead consider linear coupling between each component, facilitating a comprehensive algebraic treatment (Diego et al., 2018; Marcon et al., 2016). Whilst linearised models can predict Turing instabilities even in complex nonlinear systems, a limitation of starting with linear models directly is that the coupling coefficients are allowed to freely vary, whereas their magnitudes are in fact determined by the nonlinear system itself. Therefore, whilst these approximations have allowed systematic analyses of reaction-diffusion circuits *in silico* – which has pointed to important guiding principles – it remains unclear to what extent their simplifying assumptions will impact the applicability of model predictions *in vivo*.

Here, instead of adding more activators/inhibitors, we seek to build models of simple ‘classic’ Turing mechanisms (i.e. one activator, one inhibitor) that more realistically capture the mechanisms of intercellular signalling than network models with linear/hill-function coupling. We move beyond idealised representations of these systems, and instead explicitly consider the multi-step nature of pathway activation (i.e. signal transduction), transcriptional regulation (i.e. feedbacks) and the diverse biochemical mechanisms of pathway inhibition (e.g. some inhibitors sequester ligands, whereas others compete for receptor binding). To deal with the substantial increase in model complexity that this entails, we developed a highly performant and freely available computational pipeline (ReactionDiffusion.jl) that simulates complex reaction-diffusion systems at scale and at speed. By combining large scale numerical simulations with formal mathematical proofs, we uncover fundamental limits on the ability of such activator-inhibitor circuits to break symmetry, which apply rather generally across many types of signalling pathways and intracellular dynamics.

Rather than all behaving similarly – as one might naively expect – we have identified eight distinct classes of activator-inhibitor system, defined by the type of inhibitory mechanism and regulatory feedbacks present. This suggests that symmetry breaking is possible with a more flexible regulatory logic than previously thought and reveals several motifs not predicted by simpler models. Moreover, we predict that symmetry breaking is achievable with biologically feasible parameter values and, in some cases, can be made robust to variations in any single biophysical/biochemical parameter. Thus, by adding complexity and realism to our models, we have revealed a more widespread ability of reaction-diffusion systems – even those that comprise only a single activator and single inhibitor – to self-organise patterns *de novo*.

## RESULTS

### ReactionDiffusion.jl: a pipeline to efficiently simulate complex reaction-diffusion systems

Intercellular feedbacks in reaction-diffusion systems couple cell states across space and time within developing tissues. These interactions can be coarse grained via a continuum approximation into systems of partial differential equations (PDEs) that describe the spatiotemporal dynamics of signalling. Whilst simple to write down, the complexity of our models (large systems of numerically stiff PDEs) makes them highly challenging to simulate. We therefore developed a suite of new computational tools to simulate these complex reaction-diffusion systems at scale.

We have built an end-to-end pipeline in the performant SciML ecosystem that leverages an expressive domain-specific-language, state-of-the-art numerical solvers and automated multicore parallelisation (Loman et al., 2023; Ma et al., 2021 preprint; Rackauckas and Nie, 2017, 2019). Models are first specified in an intuitive syntax that naturally reflects the regulatory interactions involved in signalling (Fig. 1B,C). Mass action kinetics are automatically prescribed for the specified biochemical reactions, and user-defined functions can be used to parameterise other reaction rates (e.g. transcriptional feedbacks). Once specified, the model is then automatically converted to a system of corresponding PDEs, and then automatically simulated to predict the resulting dynamics, all within a single line of code (Fig. 1C). We have implemented highly-performant, adaptive time-stepping solvers that allow systems of more than ten PDEs to be simulated interactively on a personal laptop (see Supplementary information).

We have also implemented Turing instability analysis into our pipeline, mirroring the approaches used in Diego et al. (2018) and Scholes et al. (2019) to predict whether a reaction-diffusion system can break symmetry (Fig. S1A). Again, this is fully automated, going from an intuitive model description to a high-throughput parameter screen in just a single line of code (Fig. S1B). We have demonstrated the efficacy of our approach by correctly identifying symmetry-breaking parameter sets for four separate Turing systems (two to four components) with known ground truths (Fig. S1C-F; Meinhardt and Gierer, 1974; Schnakenberg, 1979; Lengyel and Epstein, 1991; Kurics et al., 2014). By employing multithreaded parallelisation and symbolic computing methods, we can accelerate these computations and are able to screen millions of parameter sets per minute on a personal laptop (see Supplementary information).

Together, these form a fully end-to-end pipeline for efficiently simulating and screening complex reaction-diffusion systems. These tools can be applied to any type/size of reaction-diffusion system and are fully automated, meaning that a detailed knowledge of numerical solvers or fine tuning of algorithm parameters is not required. We have made our pipeline freely-available as an open-source Julia package with extensive documentation and tutorials (https://github.com/hiscocklab/ReactionDiffusion.jl).

### The ability of reaction-diffusion systems to break symmetry is relatively insensitive to their intracellular dynamics

Armed with our new pipeline, we are now able to simulate reaction-diffusion models with sufficient complexity to more accurately mirror intercellular signalling mechanisms. However, despite the significant acceleration in simulation time, the curse of dimensionality still constrains our ability to screen large systems of PDEs. For example, the GDF5 signalling model in Fig. 1 has 20 free parameters and 14 components. Whilst we can simulate this rapidly for a single parameter set, to exhaustively screen through all combinations remains computationally unfeasible. Therefore, we sought strategies to render comprehensive screens tractable, allowing us to derive generally applicable limits regardless of parameter values (many of which are often not quantified *in vivo*).

**Fig. 2.**
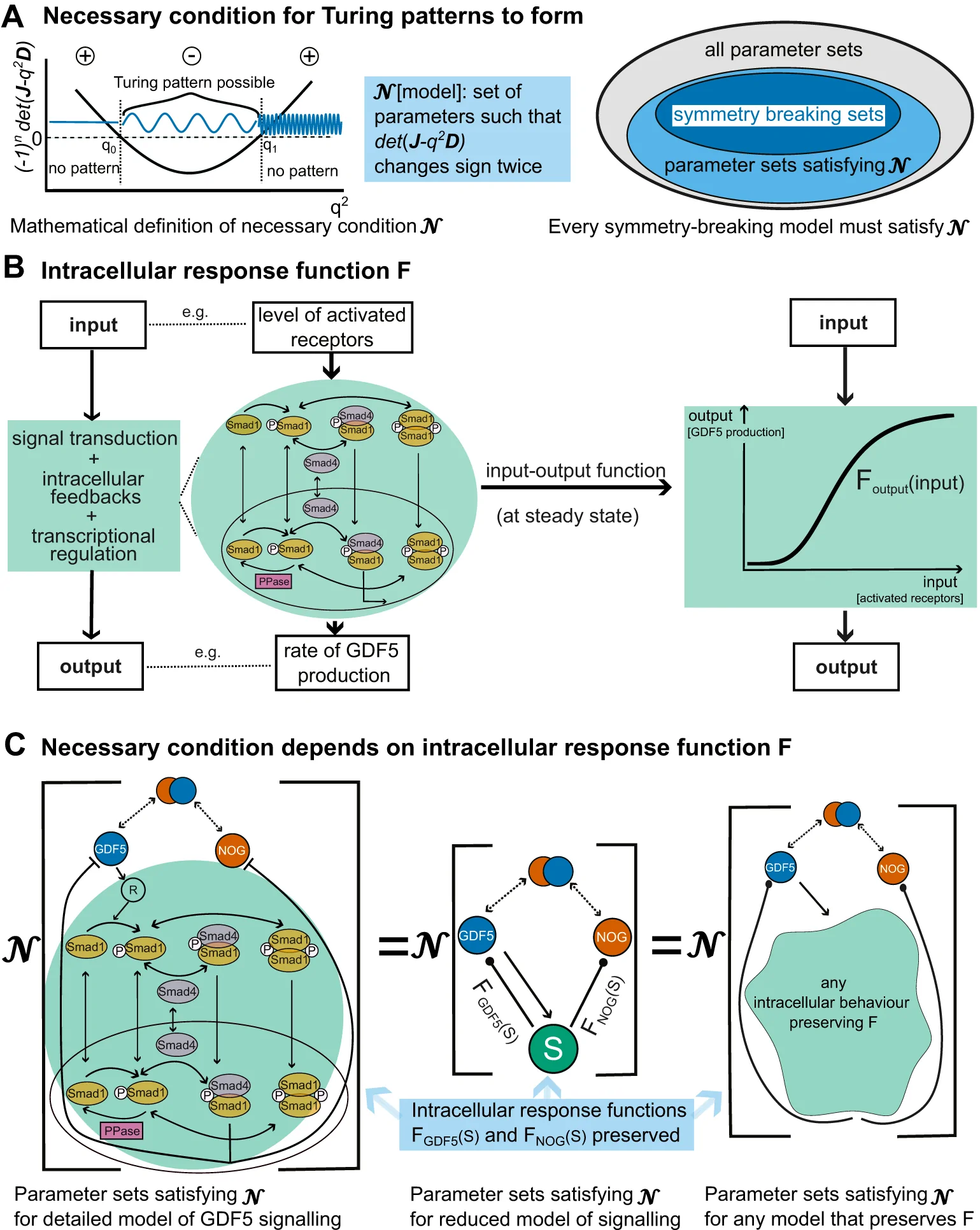
**Necessary conditions for any reaction-diffusion system to break symmetry.** (A) Schematic of a necessary condition for symmetry breaking, 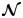, which requires the determinant of the linearised reaction-diffusion matrix to change sign for some finite range of pattern wavevectors, *q*. (B) The intracellular response function, F_output_(input), approximates intracellular interactions (e.g. signal transduction, transcriptional feedbacks) by a single input-output function per pathway, assuming that all intracellular components have reached steady state. (C) The necessary condition, 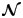, of a reaction-diffusion system can be computed without full knowledge of its intracellular dynamics, provided the steady-state intracellular response function F_output_(input) is known. Lines ending in a filled circle correspond to feedbacks from intracellular signals to extracellular components; in general, these can be either activating or repressive interactions.

To achieve this, we focused on one property of reaction-diffusion systems in particular – their ability to break symmetry i.e., the very earliest step in self-organised patterning. Biologically, this corresponds to an initially uniform tissue spontaneously self-amplifying small stochastic fluctuations in gene expression/protein concentrations across a specific range of spatial wavelengths. The advantage of focusing on the symmetry breaking step is that, even for complex nonlinear systems, the conditions for symmetry breaking can be derived via linear stability analysis. In particular, for a Turing-like instability to arise, there must be some small perturbations with wavenumber *q* (equivalently, periodic patterns with wavelength *λ*=2*π*/*q*) which destabilise an initially homogeneous state (Murray, 2008). One simplification that has been used previously is to examine the determinant of the linearised reaction-diffusion matrix, 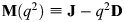 (Diego et al., 2018; Smith and Dalchau, 2018), where 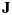 is the Jacobian of the reaction terms and 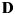 is a diagonal matrix corresponding to diffusion. The Turing instability criterion requires 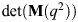 to change sign twice in the range *q*∈[0, ∞) (Smith and Dalchau, 2018), which we hereafter refer to as the necessary condition 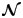 (Fig. 2A). We note that this linear instability analysis does not guarantee that a self-organised pattern will eventually form, but it is sufficient to determine whether a given reaction-diffusion system can break symmetry. In other words, 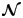 can predict whether a system can initially break symmetry but does not predict what patterns will finally self-organise.

When considering detailed reaction-diffusion models (e.g. like Fig. 1B), we noticed that their structure greatly simplifies the calculation of 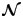 (see Supplementary information). In general, signalling pathways comprise elements which diffuse between cells (e.g. extracellular ligands and inhibitors; we label these as *D*); elements which directly interact/bind to the diffusible elements (e.g. receptors; we label these as *R*); and elements which are confined within the cell itself (e.g. intracellular signal transduction elements; we label these as *I*). We may split 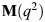 accordingly into blocks, so for example 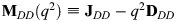 represents interactions/diffusion of the extracellular components *D*, whereas 
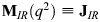
 reflects how the intracellular elements *I* are regulated by (activated) receptors *R*. Whilst the intracellular components, *I*, add significant complexity to the models, they do not contribute to intercellular diffusion (i.e. 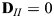) and are not directly affected by the extracellular species (i.e. 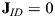). Using Schur's lemma, we therefore find that the determinant of 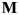 may be expressed in a simpler form:

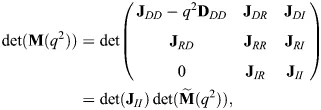
where 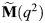 is defined as:

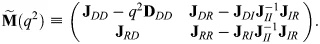
Since 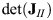 is a constant, it follows that the necessary condition, 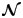, may be computed by considering a much simpler system, 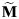, which has precisely the same extracellular interactions (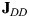, 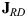) and diffusion 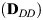, but with all receptors and intracellular components removed and replaced with a single, non-diffusing component per signalling pathway (we have labelled these effective intracellular components as *S*). The overall effect of intracellular signalling on the diffusible elements is then captured by 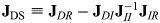, and the dynamics of intracellular signalling is represented by 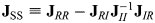. At first glance, the computation of these effective interactions remains challenging and appears to depend on the full complexity of the intracellular components. However, we may use Lemma 2.1 from Smith and Dalchau (2018) to show that these terms can be computed by simply assuming that all intracellular components, *I*, reach quasi-steady state i.e. do not change over time. Therefore, we may rigorously approximate the intracellular dynamics as a ‘black box’ that takes in inputs (e.g. levels of activated signalling receptors) and translates these into outputs (e.g. production rates of signalling ligands and inhibitors), with each steady-state input-output relation (Fig. 2B) quantified by a corresponding intracellular response function, F_output_(input). The advantage of this formulation is that the necessary condition 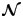 may now be computed by considering the intracellular response functions alone without a detailed characterisation of the intracellular dynamics, which are often complex and hard to quantify. Moreover, we show that this approach is also sufficient to predict the possible wavelengths of pattern that can break symmetry, as well as the phase relations between gene expression domains i.e., whether they are in or out of phase (see Supplementary information).

**Fig. 3.**
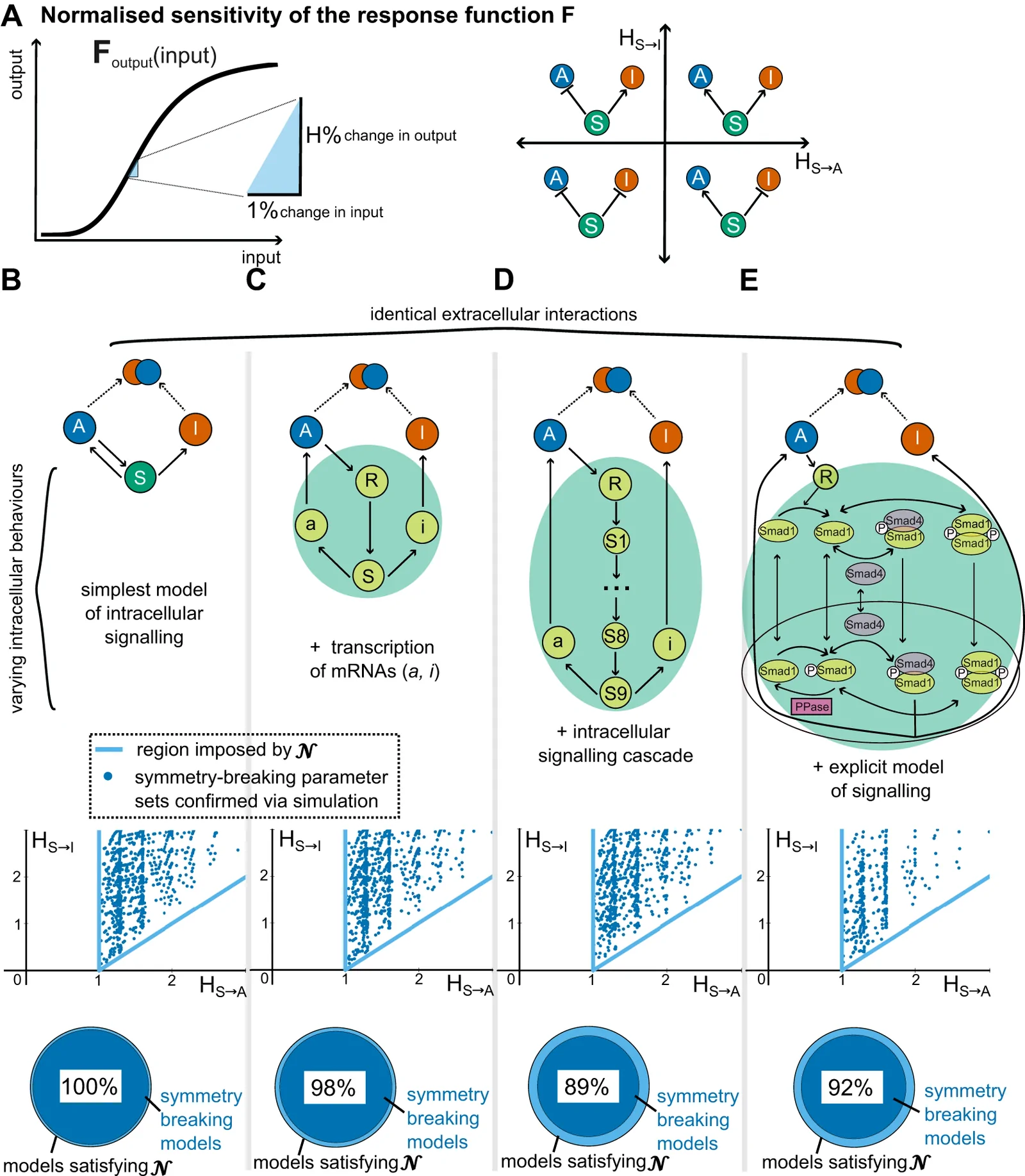
**The ability of activator-inhibitor systems to break symmetry is relatively insensitive to their intracellular dynamics.** (A) The normalised sensitivity, H_input→output_, approximates the effect of a given intracellular feedback by a single number, which reflects how the output responds to changes in the input. H>0 corresponds to activating, and H<0 to repressive interactions. (B-E) Analysis of activator-inhibitor systems with the same extracellular but different intracellular interactions. All models assume that the extracellular inhibitor directly binds to the signalling ligand targeting it for degradation. A variety of intracellular behaviours are considered (schematics, upper row). Regardless of the intracellular network, we find that the sensitivity parameters are subject to the same constraints across all models (light blue lines, middle row) which is confirmed via numerical simulation (blue dots, middle row). By simulating many combinations of parameters for each model, we find that the set of symmetry-breaking parameters can be reasonably approximated by the necessary condition 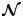 alone (∼90% agreement, lower row), and is thus similar across all models despite differences in their intracellular regulation.

There are multiple ways to measure F_output_(input) in experiments. The most direct would be to experimentally vary the input *in vitro* (e.g. via dosed application of exogeneous ligand to low density cultures) and then quantify the resulting output (e.g. via qPCR quantification of relevant transcript levels). Alternatively, one could use *in vivo* correlations to quantify how natural variations in inputs (e.g. levels of pathway activation measured via immunofluorescence) lead to changes in output levels (e.g. gene expression levels measured via fluorescent *in situ* hybridisation). Moreover, even if F_output_(input) cannot be measured directly, its salient features can often be estimated or at least bounded. We later show that the ability of reaction-diffusion systems to break symmetry depends only on the normalised sensitivity of the intracellular response functions (Paulsson, 2004); this is defined as the percentage change in output for a given percentage change in input, i.e. H_input→output_≡(ΔF_output_/F_output_)/(Δinput/input). These normalised sensitivities, H_input→output_, have an intuitive interpretation – they can be viewed as the effective hill coefficients of the response functions; if H>0, then signalling activates the prescribed output, whereas if H<0, then it inhibits the prescribed output (Fig. 3A). These sensitivities can either be directly estimated from data (e.g. Park et al., 2019), or bounded by biophysical arguments (e.g. Martinez-Corral et al., 2024).

Taken together, these results provide strict limits on symmetry breaking (via the necessary condition 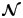) that can be derived without quantifying the full dynamics of intracellular signalling and regulation and can be deduced by considering a simpler model in which all intracellular components reach quasi-steady state. We emphasise that these limits are not approximate nor do they rely on any separation of timescales between intracellular and extracellular components. Instead, the necessary condition 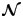 of systems with arbitrarily complex intracellular dynamics (including oscillations, time averaging, other temporal filtering) may, counterintuitively, be determined exactly without explicitly considering these dynamics. This does not imply that these dynamics will have no impact on patterning, but rather that they do not affect the necessary condition 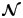, and thus the ability of the circuit to break symmetry. Therefore, there exist general limits on symmetry breaking that will be broadly applicable regardless of the precise intracellular feedback mechanisms – or intracellular parameters – at play (Fig. 2C). This result is unexpected, as previous work has highlighted the importance of considering non-diffusible species in reaction-diffusion circuits (Diego et al., 2018; Marcon et al., 2016), but we have found that a single non-diffusible component per signalling pathway is sufficient to compute 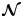.

To complement our formal analysis, we have offered an intuitive explanation of our proof (see Supplementary information) and a concrete biological example where these results have been applied (see Box 1). We have also examined the role of intracellular dynamics by using our ReactionDiffusion.jl pipeline to numerically determine whether a given system breaks symmetry (i.e. satisfies both necessary and sufficient conditions for a Turing instability). In particular, for a given set of extracellular interactions, we considered a variety of intracellular dynamics; e.g. a single, effective intracellular signal (Fig. 3B); incorporation of mRNA transcription (Fig. 3C); a multi-step intracellular signalling cascade (Fig. 3D); and an explicit model of GDF5 signal transduction (Nunns and Goentoro, 2018; Schmierer et al., 2008) (Fig. 3E). We found that any parameter set that is predicted to break symmetry satisfies the necessary condition 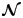 across all models considered (Fig. 3); this is also the case when assuming alternative extracellular interactions (Fig. S2A-D, Fig. S3). Moreover, although an overestimate, we found that 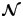 is often a close approximation (e.g. >90% overlap) to the true symmetry-breaking parameter space (see Supplemental information). Taken together, this confirms that the general limits that we derive from 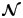 – which apply regardless of the precise intracellular dynamics – place tight constraints on the ability of reaction-diffusion systems to break symmetry.

Box 1. Symmetry breaking without a short-range activator and long-range inhibitor To illustrate how 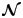 may be applied to understand symmetry breaking *in vivo*, we considered one example activator-inhibitor model in more detail, specifically one that comprises two distinct signalling pathways, WNT and BMP, in which WNT assumes the role of the Turing-like activator (i.e. undergoes positive feedback), and BMP acts as the Turing-like inhibitor. Indeed, there is experimental support for WNT-BMP circuits being involved in symmetry breaking in a variety of tissues, for example in the developing digits (Raspopovic et al., 2014) and fingerprint ridges (Glover et al., 2023). Previous theoretical work has suggested constraints on the diffusive range of these signals for symmetry breaking to occur. In classic two-component Turing models (Gierer and Meinhardt, 1972), the diffusivity of the BMP ligand must be strictly larger than the diffusivity of the WNT ligand (A). However, it was found that adding a single intracellular component to the models alleviated the requirement for differential diffusivity (Marcon et al., 2016), but the overall diffusive range of BMP (defined by both the diffusivity and extracellular half-life, 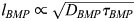) must still be higher than the overall diffusive range of WNT (B). Based on the mathematical insights provided by 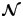, we considered how these constraints might change when adding further intracellular components to the models. Initially, we incorporated just two intracellular components, one to represent WNT signal transduction (i.e. βCAT) and the other to represent BMP signal transduction (i.e. pSMAD). We predict that symmetry breaking is now possible even if WNT and BMP have identical diffusive ranges (i.e. same diffusivity and half-lives), provided there is sufficiently strong positive feedback on WNT signalling (C). Thus, it seems that progressively adding intracellular components to the models reduces the constraints on the diffusive ranges of WNT and BMP, consistent with previous theoretical arguments (Marcon et al., 2016). One might imagine, therefore, that adding even more intracellular components would reduce further the requirements on WNT and BMP to break symmetry; indeed, a more detailed five-component model of WNT-BMP interactions in the developing digits was reported to be highly robust to parameters (Marcon et al., 2016). However, we have shown, rather generally, that the limits derived from 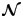 for a WNT-BMP system with just two intracellular components (one per signalling pathway) are exactly equivalent to those derived for WNT-BMP models with any more complex intracellular scheme (D). Therefore, whilst the requirements of differential diffusivity (A) and differential diffusive range (B) are not fundamental, but instead a result of model oversimplification, the requirement for strong positive feedback (C) is a general limit that must apply for all WNT-BMP systems of this type no matter how complex the intracellular circuitry is. Finally, we note that this property is not universal to all types of activator-inhibitor mechanism – for example, as outlined in the Supplementary information, a WNT-DKK circuit must always have differential diffusive range between WNT and DKK for symmetry breaking to occur.

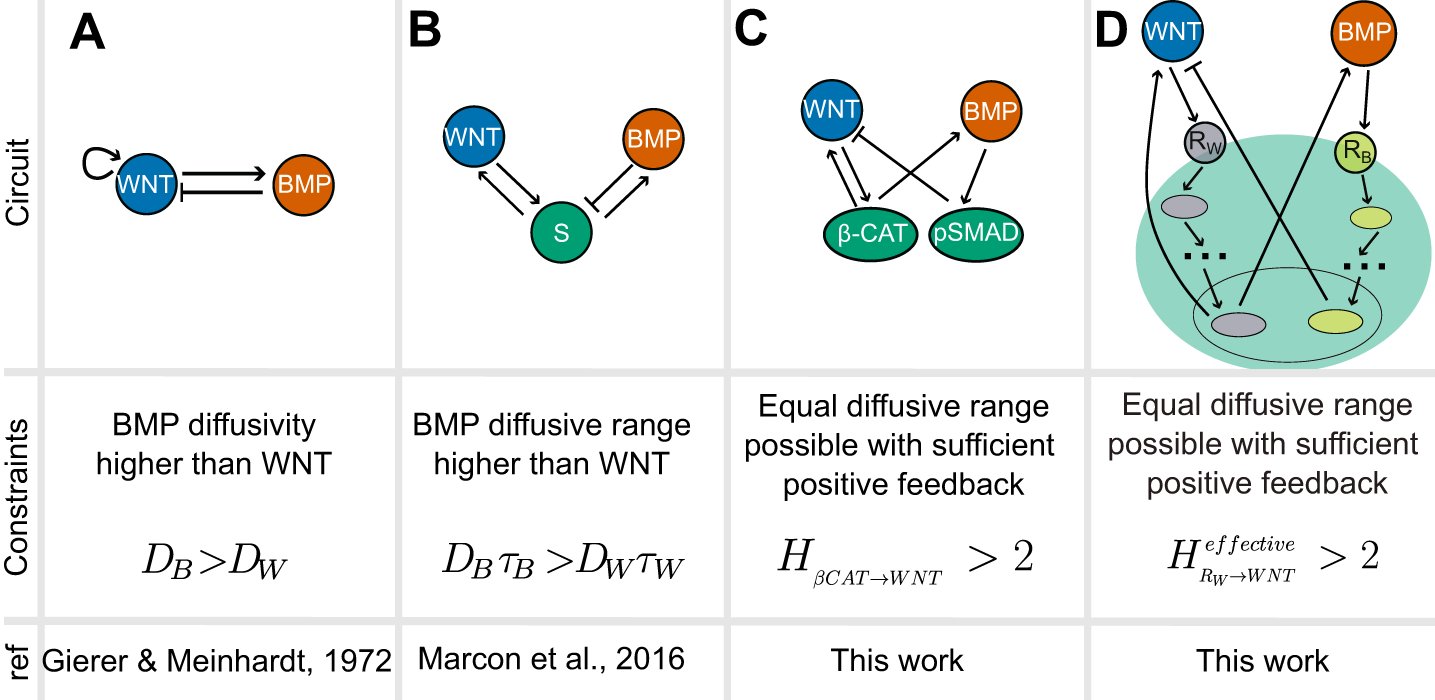

**Impact of intracellular complexity on symmetry breaking of WNT-BMP circuits**. Upper: schematised WNT-BMP circuits with increasing intracellular complexity, containing no intracellular components (A), one intracellular component (B), two intracellular components (C) and unlimited intracellular components (D). Lower: constraints on WNT-BMP diffusion required for symmetry breaking. Differential diffusive range is not required provided models do not oversimplify below two intracellular components. In B, S corresponds to a single intracellular factor that integrates WNT and BMP signalling. In D, R_W_/R_B_ correspond to activated WNT/BMP receptor complexes, which then activate downstream intracellular feedbacks of arbitrary complexity.

### Inhibitor type strongly constrains the regulatory logic of symmetry breaking activator-inhibitor circuits

**Fig. 4.**
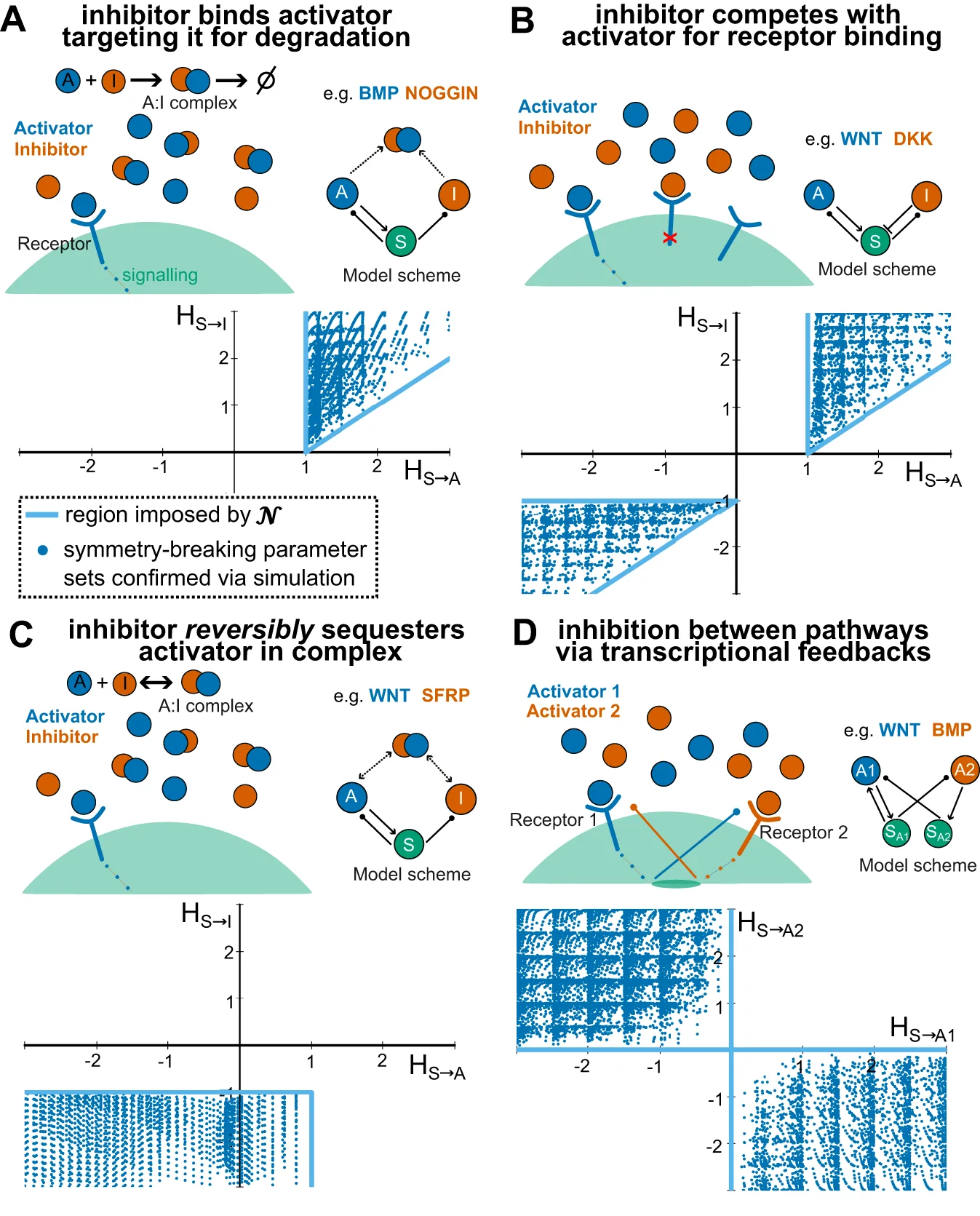
**The type of extracellular inhibitor strongly constrains the regulatory logic of symmetry-breaking activator-inhibitor circuits.** (A-D) Analysis of activator-inhibitor systems with different types of extracellular interactions (schematics, upper) reveal strict constraints on feedback regulation required for symmetry breaking to occur (visualised via the normalised sensitivities H_S→A_, H_S→I_, lower). Shown are analytical limits that apply regardless of the intracellular dynamics (light blue lines), as well as numerical simulations of models in which signalling is approximated by a single variable (blue dots). Parameter ranges were chosen to adequately explore the space defined by the necessary condition 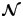. Lines ending in a filled circle in the model schemes correspond to feedbacks from intracellular signals to extracellular components; in general, these can be either activating or repressive interactions. (A) When inhibitors bind directly to signalling ligands, sequestering them into inert complexes that eventually degrade, both the activator and the inhibitor must be activated by signalling to break symmetry. (B) When inhibitors bind to receptors but do not activate them – competing with ligand-induced signalling – the system permits symmetry breaking only if signalling either activates or inhibits the expression of both activator and inhibitor. (C) When inhibitors reversibly bind to ligands, forming complexes that cannot activate signalling but can dissociate to release the free ligand, signalling must inhibit the expression of the inhibitor. The activator may be activated, inhibited or unaffected by signalling. (D) When inhibitors act indirectly on the activator, i.e., ligands activate other signalling pathways, symmetry breaking requires one pathway to activate expression of its own ligand and exactly one pathway to inhibit the ligand of the other.

So far, we have explored how different intracellular dynamics impact the capacity of reaction-diffusion models to break symmetry. Next, we investigated the effect of incorporating different extracellular interactions. We restricted our attention to activator-inhibitor systems (i.e. those with just two secreted molecules that either activate or inhibit signalling) and explicitly modelled the mechanism of pathway inhibition. This led us to consider four distinct types of (diffusible) inhibitor: (1) inhibitors that bind directly to signalling ligands, sequestering them into an inert complex that eventually degrades (Fig. 4A); (2) inhibitors that bind to receptors but do not activate them, thus competing with ligand-induced signalling (Fig. 4B); (3) inhibitors that reversibly form complexes with ligands, in which the complex cannot activate signalling but can dissociate to release the ligand (Fig. 4C); (4) inhibitors that act indirectly on the activator i.e. ligands that activate other signalling pathways, which then leads to transcriptional inhibition of the activator (Fig. 4D). For each inhibitor type, we sought to understand what regulatory interactions were compatible with symmetry breaking; for example, the classic activator-inhibitor scheme posits that both activator and inhibitor are induced by signalling (H_S→A_>0, H_S→I_>0).

We took two complementary approaches. First, we derived general analytical limits on the normalised sensitivities, that apply regardless of the assumed intracellular networks and across all intracellular/extracellular parameter values (light blue lines in Fig. 4; see Supplementary information). Second, we performed numerical simulations assuming a specific intracellular network (for simplicity, a single effective component, *S*, per pathway) and systematically varied parameters to plot a wide range of symmetry-breaking parameter sets (blue dots in Fig. 4; compared directly with 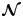 in Fig. S3).

Strikingly, we found that each type of diffusible inhibitor requires distinct regulatory circuits to self-organise patterns *de novo*: (1) When the inhibitor irreversibly sequesters the activator by directly binding to it, then both activator and inhibitor must be activated by signalling for symmetry breaking to occur (Fig. 4A); this is consistent with the intuition from classic activator-inhibitor models. (2) When the inhibitor instead competes with the activator for receptor binding, then an additional type of regulation is also compatible with symmetry breaking, in which signalling inhibits both activator and inhibitor expression (Fig. 4B). (3) When the inhibitor binds the ligand, but the resulting complex can readily dissociate, then the classic activator-inhibitor logic (i.e. signalling activates both activator and inhibitor) no longer permits symmetry breaking. Instead, signalling must always inhibit expression of the inhibitor (Fig. 4C). The activator may also be inhibited by signalling, leading to a similar circuit as above in (2). However, it is also possible that the activator is itself activated by signalling, meaning that activator and inhibitor are no longer co-expressed, a scenario not predicted by simpler activator-inhibitor models. Finally, symmetry breaking appears to be possible even if the activator is uniformly expressed and not impacted by signalling level at all. (4) When inhibition acts indirectly (i.e. when there are two diffusible ligands activating different signalling pathways), we found that one pathway must activate expression of its own signalling ligand (see Supplementary information); without loss of generality, we defined this to be pathway 1. We also found that a single inhibitory interaction between the pathways is required (Fig. 4D), i.e. activation of pathway 1 inhibits the expression of ligand 2 (or vice versa).

We also considered a more general case that combines scenarios (1) and (3), in which the inhibitor and activator form a complex that both degrades and dissociates. We found that the circuits able to break symmetry are a combination of the limiting cases defined by scenarios (1) and (3), with different circuits possible based on the ratio of the complex dissociation rate to the complex degradation rate (Fig. S4).

Together these results demonstrate that, unlike the intracellular components, the extracellular interactions in reaction-diffusion systems strongly impact their symmetry-breaking potential and lead to qualitatively distinct types of activator-inhibitor circuit. To give a concrete example, consider activator-inhibitor circuits based on the WNT signalling pathway, with two common families of inhibitors: DKKs and sFRPs. Whilst DKKs and sFRPs are both inhibitors of WNT signalling, their (extracellular) mode of action is rather different; DKKs bind to LRP5/6 co-receptors preventing formation of an activated receptor complex, whereas sFRPs inhibit WNT ligands by binding to them directly. Our analysis shows that this results in qualitative differences (i.e. distinct regulatory circuits capable of symmetry breaking) when comparing WNT-DKK and WNT-sFRP systems. We emphasise that the constraints on the type of regulatory interactions may be derived from the necessary condition 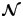 alone and thus depend only on the type of diffusible inhibitor present and not on the precise intracellular dynamics assumed.

### An atlas of pattern-forming activator-inhibitor circuits

**Fig. 5.**
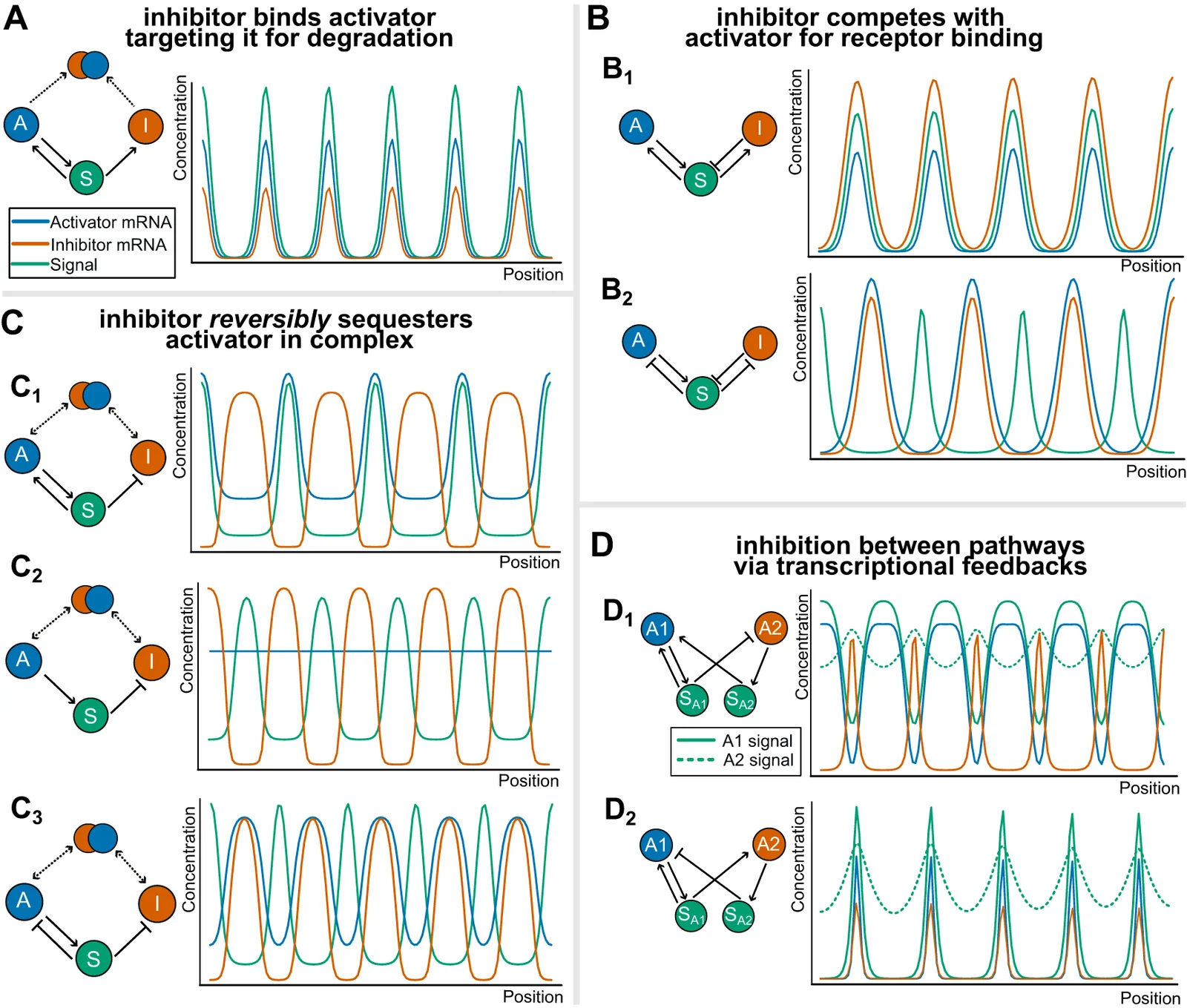
**An atlas of symmetry-breaking activator-inhibitor circuits.** (A-D) Predicted expression patterns for each type of inhibitory mechanism and corresponding regulatory circuit(s) from Fig. 4. Plots show spatial distributions of relative mRNA concentrations for the activator (blue) and inhibitor (red), alongside levels of signalling pathway activation (green). (A) Pathway activation is co-expressed (in-phase) with both the activator and inhibitor expression domains. (B) Pathway activation is either in-phase (B_1_) or out-of-phase (B_2_) with both the activator and the inhibitor. (C) Pathway activation is out-of-phase (anticorrelated) with the inhibitor, and is either: in-phase with the activator (C_1_), uniform across the tissue (C_2_) or out-of-phase with the activator (C_3_). (D) The two ligands of the two signalling pathways are either expressed out-of-phase with each other (D_1_) or co-expressed in the same location (D_2_).

For each type of inhibitor, and corresponding regulatory circuit(s) identified in Fig. 4, we used numerical simulations via ReactionDiffusion.jl to predict the resulting classes of self-organised patterns (Fig. 5). One key difference amongst them is the predicted phase difference between components. Regions of active signalling may be in-phase (i.e. co-expressed) with both the ligand and inhibitor expression domains (Fig. 5A,B_1_). Alternatively, signalling may be out-of-phase (i.e. anticorrelated) with both ligand and inhibitor expression (Fig. 5B_2_,C_3_). Beyond these two scenarios, the ligand need not always be co-expressed with the inhibitor but may instead be expressed in regions where inhibitor levels are low (Fig. 5C_1_) or present uniformly throughout the tissue (Fig. 5C_2_). Finally, when two distinct signalling pathways are involved, the two ligands may either be anticorrelated and thus expressed in anti-phase to each other (Fig. 5D_1_) or co-expressed in the same location (Fig. 5D_2_). These phase differences provide concrete, qualitative predictions – independent of specific parameter values – that may then be compared to measurements of gene expression patterns *in vivo*.

We also explored the parameter-dependence of symmetry breaking for each of the circuits. We have found, and show with examples in Fig. S5, that there exist parameter values that are predicted to break symmetry in a biologically plausible range. Moreover, for these representative parameter sets, we have shown that symmetry breaking can still occur despite significant variation in any of the individual parameters of the models (Fig. S5, left column). This suggests that, at least for the regions of parameter space shown here, the ability of activator-inhibitor circuits to break symmetry can be highly robust to parameters. We also computed robustness metrics for each parameter individually by calculating the fraction of parameter space that breaks symmetry (Fig. S5, right column). This reveals general tendencies that affect the ability of each of the circuits to self-organise; for example, we predicted that a highly diffusive complex of activator and inhibitor increases the likelihood of the circuits in Fig. S5C being able to break symmetry. We found that these broad tendencies can be derived by considering the necessary condition 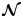 alone and are therefore likely to apply regardless of the precise intracellular dynamics present.

## DISCUSSION

Activator-inhibitor circuits have been widely hypothesised to self-organise patterns in a variety of developing tissues (Kondo and Miura, 2010; Marcon and Sharpe, 2012). Here, we develop activator-inhibitor models that are more closely aligned to *in vivo* signalling mechanisms and find that they display qualitatively distinct properties to simpler, phenomenological approximations. In particular, the mode of extracellular inhibition strongly constrains the type of regulatory architectures capable of self-organising patterns *de novo.* In contrast, the ability to break symmetry is relatively insensitive to the intracellular dynamics and depends only on the overall, steady-state response of the cell to signalling, which can often be approximated or estimated directly from data. We have combined formal mathematical proofs with large scale numerical simulations to support the generality of our conclusions and derive strict constraints on symmetry breaking (via the necessary condition 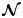) which apply regardless of intracellular signalling mechanisms.

It is important to note that an inherent limitation of our approach is that the necessary condition 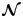 applies only to phenomena which are genuinely symmetry breaking i.e. patterns that self-organise *de novo* within homogeneous tissues. The constraints we have derived are thus necessary for a circuit to break symmetry, but do not predict whether a stable self-organised pattern will eventually form, nor the properties of that pattern (e.g. the amplitude, shape, and spacing of each periodic repeat, or its dependence on tissue geometry; Woolley, 2022, 2025b). For example, in the case of multistable reaction-diffusion systems, there can be an initial symmetry breaking from one of the stable states, but for some parameters the resulting periodic pattern is transient and eventually disappears to be replaced by an alternative (homogeneous) steady state (Krause et al., 2024; Scholes et al., 2019). Moreover, our analysis does not exclude the presence of other instabilities [for example, patterns may also self-organise via oscillatory dynamics (Oates et al., 2012), localised wavefronts (Rulands et al., 2013), mass-redistribution (Brauns et al., 2020) or wave-pinning type interactions (Mori et al., 2008)] and is formally only exact in the limit of low noise. Nevertheless, provided we restrict our attention solely to whether a given mechanism can break symmetry, then 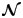 does provide strict, broadly applicable conditions that are necessary (but not sufficient) for symmetry breaking to occur.

Guided by these necessary conditions, we systematically characterise systems with only a single activator and a single inhibitor, aiming to comprehensively enumerate symmetry-breaking circuits and identify key gene expression signatures associated with each class of mechanism. These include the classic Turing-type logic, in which signalling promotes the simultaneous expression of both activator and inhibitor, as observed for example with WNT/DKK in hair follicle patterning (Sick et al., 2006) and Nodal/Lefty in early embryogenesis (Müller et al., 2012). However, several other circuits break symmetry but have an inverse relationship between signalling and activator/inhibitor production. Indeed, recent experimental observations point to numerous examples of this inverted logic operating *in vivo*, including GDF5/NOG in the developing joints (Grall et al., 2024) and TGFβ2/Fst in the optic cup (Grocott et al., 2020). Importantly, our computational atlas provides an explanation for these different circuits, highlighting the essential role that extracellular interactions play in dictating what regulatory logic is compatible with symmetry breaking.

In addition to recovering known motifs, when we allow the inhibitor to reversibly bind the signalling ligand, we predict several further types of circuit capable of breaking symmetry. This includes cases where activator and inhibitor are expressed in opposite locations, a property not predicted by simpler activator-inhibitor models, but something that has been observed for several self-organising patterns *in vivo* (e.g. WNT/sFRP in cortical organoids; Takata et al., 2017). We speculate that this may be a previously unrecognised mechanism for symmetry breaking. Moreover, we predict that an elevated diffusivity of the ligand in complex promotes symmetry breaking (Fig. S5C), suggesting that ligand shuttling [as has been suggested for WNT/sFRP (Mii and Taira, 2009, 2011), but also other systems e.g. BMP/Chordin (Zhu et al., 2023)] may not only be relevant for shaping/scaling morphogen gradients (Shilo et al., 2013), but may also be involved in symmetry breaking.

Finally, when multiple signalling pathways are operating, we predict a variety of possible expression patterns, as observed and modelled across several *in vivo* systems, including patterning of the digits (Raspopovic et al., 2014) or the fingerprints (Glover et al., 2023). Together, our comprehensive atlas of activator-inhibitor mechanisms reveals a more varied repertoire of symmetry-breaking motifs than previously recognised, which may be operating across a diverse range of tissues *in vivo*.

We highlight that our computational atlas of activator-inhibitor motifs naturally complements results from existing *in silico* screens. Whereas these previous studies systematically varied the topology of interaction networks – assuming coupling via linear or hill functions – we instead took a more targeted approach by considering specific molecular mechanisms associated with signalling. Our mathematical results (i.e. the limits derived from 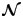) provide a rigorous basis for classifying circuits based purely on the type of extracellular inhibitor involved. Some of the circuits we describe can be mapped to topologies identified in previous high-throughput screens [e.g. the type II networks analysed in Marcon et al. (2016)]; however, we have also uncovered mechanisms that had not previously been associated with symmetry breaking (see Box 2). More generally, there are instances where a topology/network-based approach may be more instructive – for example, performing fast algebraic analysis of linear models with RDNets (Marcon et al., 2016) or numerical analysis of generic network architectures (Scholes et al., 2019; Zheng et al., 2016) – but in other cases a more explicitly mechanistic model, as we have explored here, may be more informative.

Box 2. A putative WNT-sFRP circuit that can break symmetry Many of the symmetry-breaking motifs in our atlas (Fig. 5) are present in activator-inhibitor circuits known to be involved in periodic patterning (see Discussion). However, the regulatory logic and resulting expression patterns associated with one circuit, namely that in Fig. 5C_1_, represents a previously unrecognised activator-inhibitor mechanism capable of symmetry breaking. We hypothesised that this type of circuit may be implemented by WNT ligands and their diffusible inhibitors, sFRPs. Consistent with the circuit in Fig. 5C_1_, sFRPs are known to be highly diffusive (Mii and Taira, 2009), they inhibit WNT ligands by directly binding to them but this binding is reversible allowing WNT ligands to be released from the WNT-sFRP complex (Mii and Taira, 2011). Moreover, WNT and sFRP expression domains are often observed to be out-of-phase with one another, for example in early embryos (Yamaguchi, 2001; Yamamoto et al., 2022), regenerating flatworms (Gurley et al., 2010) and symmetry-breaking cortical organoids (Takata et al., 2017). Traditionally, this type of pattern – in which the domains of ligand and inhibitor expression are opposed to each other – is not associated with symmetry breaking, but rather in shaping morphogen gradients; for example, operating as an expander-repressor type circuit. Indeed, all other mechanisms in our atlas require ligand and inhibitor to be co-expressed in the same domain for symmetry breaking to occur (excluding the case of multiple signalling pathways). However, to explore the possibility that the observed antiphasic WNT-sFRP patterns may self-organise *de novo*, we simulated the dynamics of a WNT-sFRP circuit in which WNT signalling promotes WNT ligand expression and inhibits sFRP expression (see Fig. S7 for code). Using parameter values informed by previous studies (Mii et al., 2021; Wawrzak et al., 2007; Yamamoto et al., 2022), we found that this mechanism can reliably break symmetry *in silico* to form patterns over biologically plausible time- and length-scales. Moreover, we found that if sFRP enhances the diffusivity of WNT when in complex – which has been confirmed *in vivo* (Mii and Taira, 2009) – then this significantly increases the likelihood that the system will break symmetry (Fig. S5C). This aligns with the concept of ligand shuttling (Shilo et al., 2013), which is typically associated with regulation of morphogen gradients (e.g. gradient scaling); we suggest that another role for ligand shuttling may be to allow the circuit to break symmetry. Together, our results identify WNT-sFRP as a novel circuit that can break symmetry *in silico*; further work will be required to evaluate to what extent it is involved in symmetry breaking *in vivo*.
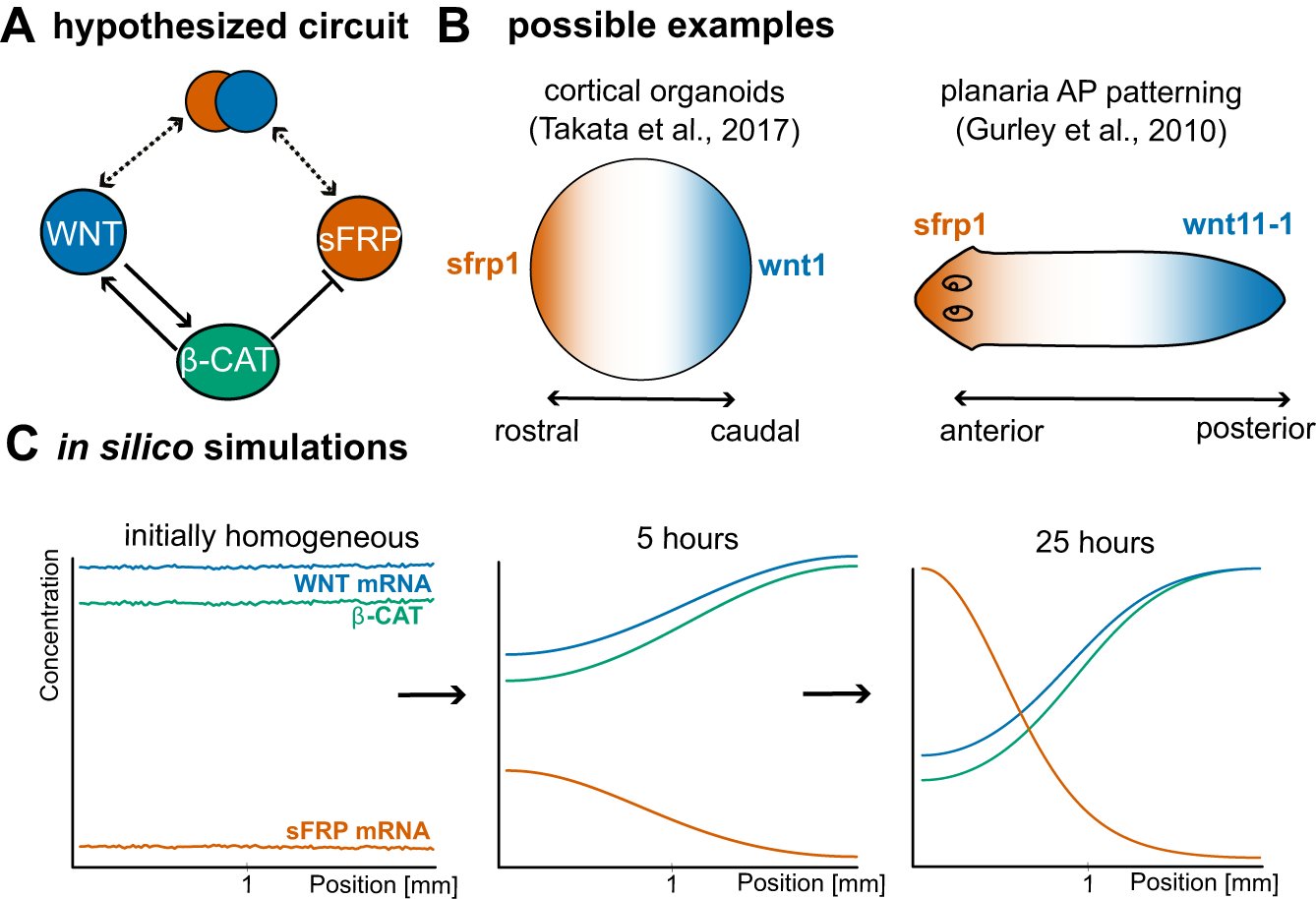

**A WNT-sFRP circuit that can break symmetry *in silico***. (A) Schematic of hypothesised regulatory feedbacks. (B) Example anti-phasic WNT-sFRP expression patterns observed *in vivo.* (C) *In silico* symmetry breaking with biologically feasible parameter values.

When exploring the parameter-dependence of our models, we find examples where symmetry breaking is both robust to parameter variation and occurs within a biologically feasible range, hinting that systems consisting of only a single activator/inhibitor pair might, by themselves, be capable of highly robust patterning. However, our analysis of model robustness is rather preliminary, characterising either isolated exemplar parameter sets (Fig. S5, left column) or collapsing the full parameter space down to one dimension for ease of visualisation (Fig. S5, right column). Further work is needed to fully characterise the high-dimensional parameter spaces and thus robustness of each of the models in Fig. 5, using tools and approaches applied elsewhere. Moreover, integrating model predictions with *in vivo* data and biophysical measurements of key parameters will also be necessary to definitively test the hypothetical circuits that we have proposed here (Kuhn et al., 2022; Müller et al., 2012).

A further limitation of this study is that we restrict our attention to mechanisms with only two secreted diffusible molecules, which considerably underestimates the complexity present *in vivo.* Whilst some *in vivo* patterns do appear to involve single activator/inhibitor pairs (e.g. GDF5/NOG in the developing finger joints; Grall et al., 2024), others are suggested to involve multiple signals and pathways [e.g. patterning of the rugae (Economou et al., 2012, 2021), or murine hair follicles (Glover et al., 2017)]. Moreover, secreted ligands/inhibitors often play more than one role in a given system as assumed here; for example, Lefty inhibits Nodal signalling by binding to both the Nodal ligand and its obligate co-receptor (Chen and Shen, 2004). Importantly, *in silico* studies have shown that adding such diffusible molecules not only expands the number of possible pattern-forming networks but can also be associated with increased robustness (Marcon et al., 2016; Scholes et al., 2019; Shaberi et al., 2025). In addition, our models have so far approximated multi-subunit receptor complexes by a single limiting receptor component (Klumpe et al., 2022); they have neglected impacts of receptor dynamics on extracellular ligands and inhibitors [e.g. receptor-mediated ligand sequestration (Lord et al., 2019; Preiß et al., 2022), endocytic ligand recycling (Romanova-Michaelides et al., 2022)] and have focused on activator-inhibitor complexes with 1:1 stoichiometries (like GDF5-NOG). Despite these simplifications, our models capture the minimal mechanistic features associated with intercellular signalling and distinct types of pathway inhibition, and this already revealed qualitative differences with phenomenological Turing models. We expect that making our models more realistic by incorporating such additional interactions and components will further expand our understanding of reaction-diffusion based patterning *in vivo*. We also hope that the principles derived from purely molecular patterning systems may also be relevant to systems that combine reaction-diffusion with cellular and/or mechanical processes.

Beyond the principles we have uncovered, the computational frameworks that we have developed are broadly applicable to any reaction-diffusion mechanism. We have built an end-to-end, freely-available simulation pipeline (ReactionDiffusion.jl) that generalises to mechanisms with any number of components/interactions and that may be used without detailed knowledge or fine tuning of PDE solvers. We are currently limited to one-dimensional simulations on a static domain, which are sufficient to determine the overall characteristics of symmetry breaking (i.e. can a pattern form *de novo*, and what components are co-expressed?) but can often not be compared directly to experimental data. Moreover, we have restricted our attention to fully self-organising systems (i.e. no previous asymmetries in the tissue) with a strong focus on periodic patterns. Future work will be required to extend our pipeline to broader classes of pattern with more realistic geometries, tissue growth, anisotropic diffusion and external/initial asymmetries, as is often present *in vivo* (Grall et al., 2024; Haupaix et al., 2018; Ho et al., 2019; Raspopovic et al., 2014)*,* and as has been achieved for simpler (two to four component) models elsewhere (Walker et al., 2023).

Together, this work has revealed strong constraints and general design principles for symmetry breaking via reaction-diffusion mechanisms. The candidate circuits we have identified may point experimentalists to new activator-inhibitor circuits that may be present *in vivo*, and also suggest promising designs for synthetic morphogen circuits that can self-organise *in vitro* (Sekine et al., 2018; Tica et al., 2024; Toda et al., 2020). Combined with experimental advances in spatiotemporal measurement and perturbation, we are now poised to further understand the implications and implementation of Turing's reaction-diffusion hypothesis *in vivo*.

## MATERIALS AND METHODS

### Simulating complex reaction-diffusion PDEs

We simulate reaction-diffusion PDEs in the performant SciML ecosystem in Julia. We have combined our scripts into a freely available, open-source Julia package: https://github.com/hiscocklab/ReactionDiffusion.jl. Briefly, PDEs are discretised in one dimension via the finite difference method, resulting in a system of ordinary differential equations (ODEs) that is then solved using the method of lines with an adaptive ODE solver suitable for stiff problems. For full details of our simulation methods, see Section 1 of the Supplementary Materials and Methods.

### Predicting Turing instabilities numerically

We have also developed scripts in ReactionDiffusion.jl to predict whether a given system of reaction-diffusion PDEs exhibits a Turing instability. Briefly, we compute a homogeneous steady state of the system by modelling the dynamics without diffusion, then we analyse the stability of this homogeneous state to periodic disturbances of a wide range of wavelengths. For full details of our automated Turing instability analysis, see Section 1 of the Supplementary Materials and Methods. We confirm the accuracy of our approach by comparing our numerical predictions to models with known ground truths; these are described in Appendix 1 of the Supplementary Materials and Methods.

### Predicting Turing instabilities analytically

In addition to our numerical analyses, we have performed algebraic linear instability analyses to derive parameter constraints for specific classes of activator-inhibitor model. This includes the derivation of a necessary condition, 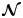, that applies broadly regardless of the intracellular dynamics of the system; for a full derivation and discussion of 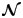, see Section 2 of the Supplementary Materials and Methods. We then apply these results to specific activator-inhibitor systems in Section 3 of the Supplementary Materials and Methods. The resulting predictions are then directly compared to numerical analyses in Section 4 of the Supplementary Materials and Methods.

### Parameter values

Rationale and description of parameter values used can be found in Section 5 of the Supplementary Materials and Methods.

## Supplementary Material



10.1242/develop.205067_sup1 Supplementary information
