# Supplementary material for: Fundamental limits on symmetry breaking by Turing-like activator-inhibitor mechanisms
Source: Development. 2026 Mar 23;153(16):dev205067. doi: 10.1242/dev.205067 (PMC13495787; doi:10.1242/dev.205067)
Supplement: Supplementary file 2 [file develop-153-205067_review_history.pdf]

# Fundamental limits on symmetry breaking by Turing-like activator-inhibitor mechanisms

Daniel Muzatko, Bijoy Daga and Tom W. Hiscock
DOI: 10.1242/dev.205067

**Editor**: James Briscoe

**Review timeline**

| | |
|---|---|
| Original submission: | 1 July 2025 |
| Editorial decision: | 31 July 2025 |
| First revision received: | 28 January 2026 |
| Editorial decision: | 9 February 2026 |
| Second revision received: | 24 February 2026 |
| Accepted: | 24 February 2026 |


---

**Original submission**

First decision letter

MS ID#: dev.205067

MS TITLE: Fundamental limits on patterning by Turing-like reaction-diffusion mechanisms

AUTHORS: Daniel Muzatko, Bijoy Daga and Tom W. Hiscock

Dear Dr Hiscock,

I have now received all the referees' reports on the above manuscript, and have reached a decision. The referees' comments are appended below, or you can access them online: please go to: ********

As you will see, the referees express considerable interest in your work, but have some significant criticisms and recommend a substantial revision of your manuscript before we can consider publication. The referees acknowledge the clarity and rigour of the analysis and they appreciate the development of the open-source software. However, there are areas that need strengthening. The referees would like to see a stronger connection made to common developmental systems studied in the field. They also request you place your work in the context of previous studies in the field. Finally, analysis of the nonlinear effects of dimerisation would strengthen the manuscript and provide new insight.

If you are able to revise the manuscript along the lines suggested, which may involve further analyses, I will be happy to receive a revised version of the manuscript. Your revised paper will be re-reviewed by one or more of the original referees, and acceptance of your manuscript will depend on your addressing satisfactorily the reviewers' major concerns. Please also note that Development will normally permit only one round of major revision. If it would be helpful, you are welcome to contact us to discuss your revision in greater detail. Please send us a point-by-point response indicating your plans for addressing the referees' comments, and we will look over this and provide further guidance.

Please attend to all of the reviewers' comments and ensure that you clearly highlight all changes made in the revised manuscript. Please avoid using 'Tracked changes' in Word files as these are lost in PDF conversion. I should be grateful if you would also provide a point-by-point response detailing

© 2026. Published by The Company of Biologists under the terms of the Creative Commons Attribution License (https://creativecommons.org/licenses/by/4.0/).

how you have dealt with the points raised by the reviewers in the 'Response to Reviewers' box. If you do not agree with any of their criticisms or suggestions please explain clearly why this is so.

Reviewer 1

*Advance summary and potential significance to field*

This manuscript investigates Turing-type pattern formation in reaction-diffusion systems incorporating nondiffusible intracellular species, modeled under the assumption of fast equilibration relative to extracellular dynamics. By applying a quasi-steady-state (QSS) approximation to eliminate nondiffusible nodes, the authors derive a reduced system involving fewer species. They then employ classical linear stability analysis, partitioning the stability matrix to assess pattern-forming instabilities, and explore parameter regimes and pattern robustness using numerical simulations implemented via a dedicated Julia package, ReactionDiffusion.jl. The study is methodologically robust, with a well-structured analytical framework and a flexible, well-documented computational tool. However, its biological insights are limited, and its theoretical contributions primarily reaffirm established principles without significantly advancing the understanding of pattern formation in developmental biology. Below, I elaborate on these points, highlighting the weaknesses and specific strengths that could boost novelty.

1 - Biological relevance remains underdeveloped
The manuscript investigates activator-inhibitor circuits within reaction-diffusion systems, focusing on how extracellular interactions constrain pattern-forming regulatory logic (lines 269-300, Figure 4). It references biologically inspired mechanisms, such as ligand-inhibitor interactions (e.g., WNT/sFRP in cortical organoids, Takata et al., 2017; GDF5/NOG in finger joint development, Grall et al., 2024), but does not anchor these generic network motifs to specific developmental processes or signaling pathways in a detailed manner. For example, the study identifies eight distinct classes of pattern-forming circuits (lines 327-328) based on phase relationships (e.g., in-phase or out-of-phase expression, Figure 5A-D), yet it does not map these motifs to well-characterized morphogenetic contexts, limb patterning, rugae or hair follicle formation.
The supplementary material provides mathematical derivations, including partial differential equations (PDEs) for receptor-inhibitor dynamics, but lacks any attempt to link these models to biological measurements, such as spatial gene expression profiles or protein concentration observed in vivo.

This absence of direct comparison with experimental data or specific developmental systems limits the study's ability to generate testable hypotheses or provide mechanistic insights into developmental processes. For a journal like Development, which prioritizes studies with strong biological grounding and experimental relevance, this lack of specific application significantly diminishes the manuscript's impact. To address this shortcoming, the authors could select a well-studied developmental system, such as BMP-Chordin signaling in Xenopus dorsal-ventral patterning (Zhu et al., 2023), and use their framework to simulate patterns, comparing outputs to experimental data to validate predictions and demonstrate biological relevance. Such an approach would strengthen the manuscript's alignment with the journal's focus on advancing developmental biology.

2 - The network structures and associated phase behaviors have been explored previously
The manuscript examines reaction-diffusion systems composed of two diffusible species (activator and inhibitor) and one or two nondiffusible intracellular species, analyzing their capacity to generate self-organized patterns with diverse phase relationships, such as co-expression (in-phase), anticorrelation (out-of-phase), or uniform expression of components (Figure 5, lines 301-314). These network architectures, particularly those involving nondiffusible nodes corresponding to signaling components, have been extensively studied in prior work, including Diego et al. (2018), Scholes et al. (2019), and Marcon et al. (2016). Notably, Marcon et al. (2016) analyzed three- and four-node networks with nondiffusible nodes representing signaling pathways, demonstrating that such architectures expand the repertoire of spatial phase relationships and enhance pattern robustness to parameter variations. Similarly, Diego et al. (2018) employed QSS reductions to simplify systems with nondiffusible components, deriving instability conditions that partition the stability matrix into diffusible and non difussible modules aligning with the manuscript's approach

© 2026. Published by The Company of Biologists under the terms of the Creative Commons Attribution License (https://creativecommons.org/licenses/by/4.0/). 

(Supplementary, Section 2.2). Scholes et al. (2019) further explored how network topology influences Turing pattern formation, identifying phase behaviors comparable to those cataloged in the manuscript's atlas (Figure 5A-D).

The current manuscript builds on this framework by simulating more complex dynamical realizations of these network motifs. While this is potentially useful, the resulting patterns and behaviors are consistent with what earlier theoretical analyses already predicted based on linearized systems. In particular, the emergence of diverse in-phase and out-of-phase profiles across species as a function of topology and diffusion ratios has already been established. The numerical simulations conducted using ReactionDiffusion.jl confirm these established patterns, such as in-phase co-expression or out-of-phase anticorrelation (Figure 5), but do not venture into novel dynamical regimes that may arise by the dimerization dynamics.

To enhance its contribution, the authors could explore uncharted dynamical regimes, such as those involving multistability that could give rise to more complex spatial patterns. In this way, their framework would generate unique predictions that extend beyond existing literature.

3 - The QSS-derived condition is mathematically valid but closely aligned with classical results
The manuscript's core analytical contribution is the derivation of a necessary condition (N) for pattern formation, achieved through a quasi-steady-state (QSS) approximation that eliminates nondiffusible species, such as receptors and receptor-inhibitor complexes, to produce a reduced system of diffusible species (Supplementary, Section 2). The stability matrix is partitioned into diffusible and nondiffusible components, and the Schur complement approach is used to compute the reduced Jacobian matrix (Equation 23), which is analyzed via classical linear stability criteria to identify Turing instabilities. This partition of the stability matrix, separating diffusible and nondiffusible dynamics, is mathematically rigorous but closely mirrors the methodology employed in Diego et al. (2018), where a similar block decomposition was used to derive instability conditions for reaction-diffusion systems with nondiffusible signaling components. The manuscript explicitly notes that the constraints derived from N depend solely on the type of extracellular inhibitor and are independent of intracellular dynamics (lines 298-300), aligning with classical Turing theory's focus on diffusion-driven instabilities. While the derivation is technically sound, it does not introduce fundamentally new mechanisms, effectively re-expressing established linear stability conditions for reduced systems. The assumption that nondiffusible components rapidly reach a unique, stable steady state is standard but limits the analysis to linear regimes.
Nonlinear scenarios, such as bistability or oscillatory dynamics, are only briefly acknowledged in the context of combined degradation and dissociation of complexes (lines 291-295) without thorough investigation. This omission is significant, as nonlinear dynamics could reveal novel pattern-forming mechanisms relevant to developmental biology. To strengthen its theoretical novelty, the authors could analyze the impact of nonlinearities, such as multistability or bifurcations (as explained below), or assess the robustness of the QSS approximation under conditions where the steady-state assumption fails, thereby extending the framework beyond classical results.

4 - Dimerization introduces nonlinearity, but its dynamic implications are not explored
The manuscript incorporates dimerization-like reactions, such as the formation of ligand-inhibitor complexes that either degrade irreversibly (Figure 4A, lines 269-274) or dissociate reversibly (Figure 4C, lines 278-285), introducing nonlinear interactions into the reaction-diffusion system. These nonlinearities are evident in the PDEs governing receptor-inhibitor dynamics (Supplementary, Equation 23, lines 310-311), which include binding ($k+[I][R]$) and dissociation ($k-[C]$) terms that could lead to multistability, bifurcations, or other complex dynamical behaviors.

Such phenomena are highly relevant to developmental biology, where switch-like behaviors and multiple attractors underpin processes like cell fate decisions and tissue compartmentalization (e.g., WNT signaling, Mii and Taira, 2011). However, the manuscript restricts its analysis to linear stability and QSS assumptions, focusing on the reduced system's Jacobian matrix (Supplementary, lines 311-314) without exploring how dimerization affects the number, stability, or spatial distribution of steady states. For instance, the interplay between binding and dissociation rates could produce multiple steady states, altering the applicability of the QSS reduction or leading to novel pattern types, such as localized domains or traveling waves.

© 2026. Published by The Company of Biologists under the terms of the Creative Commons Attribution License (https://creativecommons.org/licenses/by/4.0/).

The manuscript does not investigate these possibilities, nor does it assess whether the QSS approximation remains valid under conditions where dimerization induces complex dynamics. This oversight limits the study's ability to uncover new mechanistic insights into pattern formation. To address this, the authors could conduct a bifurcation analysis to examine how dimerization parameters (e.g., k+, k−, δC) influence steady-state multiplicity or pattern robustness, or simulate scenarios where nonlinear effects yield distinct patterns, such as those observed in developmental systems with bistable signaling pathways. Such an analysis would significantly enhance the manuscript's theoretical and biological relevance.

5 - The software tool is useful but not sufficiently applied
The ReactionDiffusion.jl package is presented as a significant contribution, offering a flexible, open-source computational framework for simulating reaction-diffusion systems with both diffusible and nondiffusible species (lines 388-389). The package is well-implemented, as evidenced by its use in generating the pattern atlas in Figure 5 (lines 301-314), which illustrates spatial distributions of activators, inhibitors, and signaling pathway activation across various circuit types (e.g., in-phase, out-of-phase, or uniform expression). Its modular design and ability to handle hybrid systems make it a valuable tool for studying Turing-type pattern formation, particularly for systems with complex extracellular interactions. However, the manuscript applies ReactionDiffusion.jl exclusively to generic activator-inhibitor motifs, producing abstract patterns without modeling specific biological signaling pathways, such as those involved limb development (e.g., Bmp-Wnt), or dorsal-ventral patterning (e.g., BMP-Chordin, Zhu et al., 2023).

This abstract focus limits the tool's demonstrated utility, as it does not generate system-specific predictions or hypotheses that could be tested experimentally. For instance, the authors could have used the package to simulate a known developmental system, such as WNT/sFRP interactions in cortical organoids (Takata et al., 2017), and compared the resulting patterns to experimental gene expression data to validate the model's predictive power. The lack of such applications restricts the study's impact, particularly for a journal like Development, which values computational tools that advance biological understanding through concrete applications. To strengthen this aspect, the authors could apply ReactionDiffusion.jl to a specific developmental context, using biologically realistic parameters derived from literature (e.g., diffusion coefficients, reaction rates) to generate testable predictions, thereby showcasing the tool's practical value and relevance to developmental biology.

6 - Literature engagement could be deepened
The manuscript cites key prior studies, including Diego et al. (2018), Scholes et al. (2019), and Marcon et al. (2016), which analyzed Turing pattern formation in reaction-diffusion systems with nondiffusible nodes corresponding to signaling components (lines 376-378). Marcon et al. (2016) specifically explored three- and four-node networks with nondiffusible signaling nodes, demonstrating how such architectures enhance pattern robustness and diversify phase relationships, while Diego et al. (2018) used QSS reductions and stability matrix partitioning to derive instability conditions, mirroring the manuscript's methodology (Supplementary, Section 3.2.4). Scholes et al. (2019) further established how network topology determines robustness and spatial phase arrangements, aligning with the manuscript's atlas of patterns (Figure 5). However, the manuscript does not sufficiently engage with these works to clarify how its findings extend or diverge from their results. For example, the classification of eight pattern-forming circuits (lines 327-328) overlaps with phase behaviors identified in Marcon et al. (2016) and Scholes et al. (2019), but the manuscript does not discuss these similarities or articulate unique contributions, such as novel circuit types or parameter regimes driven by extracellular interactions.

The lack of deep engagement with these prior works obscures the manuscript's novelty and limits its scholarly impact. To address this, the authors should provide a comprehensive comparison with Diego et al., Scholes et al., Marcon et al., and Zhang et al., explicitly highlighting how their analysis advances the field, such as through new insights into extracellular inhibitor mechanisms or specific pattern-forming circuits not previously identified.

Conclusion and recommendation
The manuscript presents a mathematically rigorous investigation of activator-inhibitor circuits in reaction-diffusion systems, leveraging a well-executed quasi-steady-state (QSS) reduction and a flexible, open-source simulation tool, ReactionDiffusion.jl. The derivation of the necessary

© 2026. Published by The Company of Biologists under the terms of the Creative Commons Attribution License (https://creativecommons.org/licenses/by/4.0/).

condition N for pattern formation and the cataloging of eight distinct pattern-forming circuits are technically sound, and the software implementation is a valuable contribution to the field. However, the study's theoretical findings largely reaffirm established principles from prior work, such as Diego et al. (2018), Scholes et al. (2019), and Marcon et al. (2016), which similarly analyzed networks with nondiffusible signaling nodes and derived instability conditions using stability matrix partitioning.

The manuscript's biological relevance is limited by the absence of specific applications to developmental systems or comparisons with experimental data, which is critical for a journal like Development. The failure to explore nonlinear dynamics, such as multistability arising from dimerization-like interactions (e.g., ligand-inhibitor complexes), and the insufficient engagement with relevant literature, including the omission of Zhang et al. (2016), further weaken its impact.

I recommend rejection in its current form but I encourage resubmission after substantial revisions to address the following:
-Clearly articulate the study's novel contributions relative to prior work by Diego et al. (2018), Scholes et al. (2019), Marcon et al. (2016), and Zhang et al. (2016), emphasizing unique insights into extracellular interaction mechanisms or circuit designs not previously explored.
-Investigate the nonlinear dynamics introduced by dimerization, such as multistability or bifurcations, through bifurcation analysis or simulations to uncover new pattern-forming mechanisms relevant to developmental biology.
-Apply the modeling framework and ReactionDiffusion.jl to a specific developmental system, such as WNT/sFRP signaling in cortical organoids (Takata et al., 2017) or BMP-Chordin in Xenopus dorsal-ventral patterning (Zhu et al., 2023), generating testable predictions or constraints validated against experimental data to enhance biological relevance.

These revisions would transform the manuscript from a methodological exercise into a conceptually significant contribution to developmental biology, aligning with Development's standards for impactful research.

*************************************References********************************************

Zheng, M., Mocarlo, Bin Shao, and Qi Ouyang. "Identifying network topologies that can generate Turing pattern." Journal of Theoretical Biology, 2016.

Reviewer 2

*Advance summary and potential significance to field*

This paper analyses reaction-diffusion models with intercellular signalling mechanisms that build upon the classic two-component Turing models. The authors demonstrate, through a combination of formal mathematics and high-throughput numerical simulation, that the capacity for pattern formation is determined by extracellular inhibitory interactions and is largely insensitive to intracellular dynamics. By providing an open-source software pipeline (ReactionDiffusion.jl) for simulating such systems at scale, the work both clarifies the design principles underlying Turing-like patterning and offers practical tools for experimentalists and theorists.

*Comments for the author*

Overall, a very nice clear and well-written paper. In fact, the only sentence I would suggest needs an edit is line 223 where "it inhibits it" is a clumsy way to express signalling inhibits the prescribed output. The only other part that could be additionally clarified is that throughout the manuscript a script form of N is used to represent the patterning conditions. However, this becomes a bold sans-serif in the figures. If the authors could match the fonts better then it would be the connection between the text and figures better.

Apart from these points the only thing that the paper needs is perhaps a few more references to current theoretical literature as a number of the comments or questions raised in the introduction are either answered or clarified.

© 2026. Published by The Company of Biologists under the terms of the Creative Commons Attribution License (https://creativecommons.org/licenses/by/4.0/).

- There is some discussion about whether N provides a necessary, or sufficient set of conditions

The following paper show that they aren't sufficient

@Article{Krause-2024-TIA,
author = {Krause, A. L. and Gaffney, E. A. and Jewell, T. J. and Klika, V. and Walker, B. J.},
title = {Turing instabilities are not enough to ensure pattern formation},
number = {2},
pages = {21},
volume = {86},
journal= {Bull. Math. Biol.},
publisher = {Springer},
year = {2024},
}

The following paper shows that they aren't necessary
@Article{Brauns-2020-MassConserving,
author = {Brauns, F. and Halatek, J. and Frey, E.},
title = {Phase-space geometry of mass-conserving reaction-diffusion dynamics},
number = {4},
pages = {041036},
volume = {10},
journal = {Physical Review X},
publisher = {APS},
year = {2020},
}

Although there is a separation of the domains, since the paper considers 1D patterns there's little mention of the effect of the domain and its boundary conditions in terms of driving pattern
@Article{Woolley-2022-BCC,
author = {Woolley, T. E.},
title = {{Boundary conditions cause different generic bifurcation structures in Turing systems}},
number = {9},
pages = {1--38},
volume = {84},
file = {:https\://link.springer.com/content/pdf/10.1007/s11538-022-01055-x.pdf?pdf=button:},
journal = {Bull. Math. Biol.},
publisher = {Springer},
year = {2022},
}

@Article{Woolley-2025-PFR,
author = {Woolley, T. E.},
title = {Pattern formation on regular polygons and circles},
number = {1},
pages = {1--44},
volume = {35},
journal = {J. Nonlinear Sci.},
publisher = {Springer},
year = {2025},
}

Since you're creating a new tool, it is probably worth highlighting the recent online development of Turing simulations
@article{walker2023visualpde,
title={VisualPDE: rapid interactive simulations of partial differential equations},
author={Walker, B. J. and Townsend, A. K. and Chudasama, A. K. and Krause, A. L.},
journal={Bulletin of Mathematical Biology},
volume={85},
number={11},

© 2026. Published by The Company of Biologists under the terms of the Creative Commons Attribution License (https://creativecommons.org/licenses/by/4.0/).

```
pages={113},
year={2023},
publisher={Springer}
}
```

In the first part of the introduction the authors highlight that Turing patterns only exist in small parameter regions, but the next two papers have fully generalised the creation of Turing patterns to exist in whatever parameter region is required.

```
@Article{Woolley-2021-BTS,
author    = {Woolley, T. E. and Krause, A. L. and Gaffney, E. A.},
title     = {{Bespoke Turing Systems}},
number    = {5},
pages     = {1--32},
volume    = {83},
journal   = {Bull. Math. Biol.},
publisher = {Springer},
year      = {2021},
}

@Article{Woolley-2025-Bespoke2,
author   = {Woolley, T. E.},
title    = {Bespoke Turing patterns with specific nonlinear properties},
doi      = {10.1098/rspa.2025.0030},
eprint   = {https://royalsocietypublishing.org/doi/pdf/10.1098/rspa.2025.0030},
number   = {2312},
pages    = {20250030},
url      = {https://royalsocietypublishing.org/doi/abs/10.1098/rspa.2025.0030},
volume   = {481},
abstract = {Turing patterns offer a mechanism for understanding self-organization in biological
systems. However, due to their flexibility, it is a mechanism that can often be abused. Here, we
construct a minimal Turing system defined by just four parameters controlling the: diffusion rate,
steady state, linear dynamics and nonlinear dynamics. Using just these four parameters, we can
construct a set of kinetics with a number of desirable properties. Firstly, we can turn any
homogeneous steady state into a Turing unstable steady state. Secondly, we can ensure that the
Turing instability appears within any chosen parameter region. Thirdly, this formulation provides an
unbounded patterning parameter space with guaranteed positive solutions. Finally, using weakly
nonlinear analysis, we demonstrate that if we have freedom in any two of the parameters, then we
can define any required pattern transition (i.e. spots-to-stripes, or stripes-to-spots) under any given
changes of one of the parameters. Thus, if a Turing system is going to be applied to understand a
specific biological system and, moreover, if it is going to be used to extrapolate predictions for
experimental perturbations, then our findings underscore the necessity of heavily restricting the
modelling components and parameter values, since any freedom could be exploited to generate
potentially contradictory predictions.},
journal  = {Proc. Roy. Soc. A},
year     = {2025},
}
```

In summary, a very nice paper that offers a new tool to analyse the applicability of Turing patterns. I hope the authors find my additional citations useful for the introduction.

Reviewer 3

Advance summary and potential significance to field

In this manuscript, Muzatko et al. investigate complex reaction-diffusion mechanisms with the aim to incorporate realistic intercellular signaling mechanisms, intracellular dynamics, and multi-step interactions. The authors introduce the practical ReactionDiffusion.jl software tool, which automates

© 2026. Published by The Company of Biologists under the terms of the Creative Commons Attribution License (https://creativecommons.org/licenses/by/4.0/).

the analysis and simulation of reaction-diffusion systems. ReactionDiffusion.jl is easy to use, well-documented, and openly available, which will likely facilitate broad usage in the field. A key claim from using this tool along with additional mathematical analyses is that extracellular interactions largely determine the pattern-forming capacity of reaction-diffusion systems, whereas intracellular circuitry matters comparatively little and can be compressed into steady-state input-output relations. Indeed, the authors show across simulations of multiple intracellular implementations that the set of predicted parameter points overlaps strongly with the true pattern-forming set. The reduction of complex PDE systems into simplified criteria depending only on extracellular and steady-state intracellular response functions is elegant and useful. The manuscript is very well written, accessible to a broad range of readers, and acknowledges the limitations of the approach. I only have a few minor recommendations that the authors could consider to bolster the conclusions and further improve the presentation of this excellent paper.

*Comments for the author*

1) Conceptual advance
a) The models are limited to a single activator and inhibitor and to one-dimensional domains for simulations. While the authors acknowledge this, many developmental systems exhibit geometry-dependent instabilities, anisotropic diffusion, and multi-ligand crosstalk that can qualitatively change patterning windows and robustness. It would be very useful for the readers if the authors could further extend their analyses. I suggest to at least include another common activator/inhibitor case, where an inhibitor can both act at the level of the receptor and the activator (Chen and Shen 2004).
b) The title includes the very general reference to "Turing-like reaction-diffusion mechanisms", but the paper exclusively focuses on activator/inhibitor systems. To support the generality implied in the title, it would be helpful if the authors could also include non-activator/inhibitor-based circuits (Marcon et al. 2016).
c) To support the statement "We have implemented highly-performant, adaptive time-stepping solvers that allow systems of 10+ PDEs to be simulated interactively on a standard desktop machine", the authors could show the limits of their approach for larger systems - such as an exhaustive Turing space characterization of a previously postulated 10-node network (Diego et al. 2018).
d) The authors found high empirical agreement between the necessary condition and true pattern-forming regimes, but there are acknowledged counter-examples, where the agreement is < 40%. Where agreement drops (e.g. due to a large activator/inhibitor diffusion ratio), it would be very useful if the authors could diagnose the mechanism. Even a heuristic explanation would guide users of ReactionDiffusion.jl about when to rely on the necessary condition compared to a full dispersion analysis.
e) As stated on page 7 of the Supplementary Information, the analysis depends on the steady-state assumption of intracellular species, which could be problematic on the time-scale of pattern formation. In systems where intracellular kinetics introduce delays, bistability, or oscillatory transients, such approximations can reshape instability loci and selected wavelengths. The empirical overlap analysis is helpful, but a more explicit treatment of when the quasi-steady approximation is justified (e.g. time-scale separation criteria) would be more compelling. It would also be very valuable to explore a counterexample with explicit delays, which are well known to play crucial roles in shaping cellular dynamics and pattern formation.
f) The binding of A and I in equation system 6 of the Supplementary Information relates well to the law of mass action, whereas Case 2 (The inhibitor binds to the receptor) actually uses A/I as the inhibitory term, which is not easily related to law of mass action kinetics. Consistent with the motivation of the authors to describe realistic signaling systems, it would be very helpful to model Case 2 consistent with the law of mass action.
g) $F_s$ is always assumed to be linear (e.g. p. 9 of the Supplementary Information). What is the outcome with non-linear versions, which are biologically more realistic?
h) The ratios and dimensionless groups used throughout the manuscript are insightful, but additional examples with real measured values - where possible - would be more useful for developmental biologists, also because the final pattern (wavelength etc.) depends on the absolute values.

© 2026. Published by The Company of Biologists under the terms of the Creative Commons Attribution License (https://creativecommons.org/licenses/by/4.0/). 

i) To at least provide an initial assessment of how a simple physical process affects the stability and scaling of the predicted patterns from Figure 5, the authors could introduce uniform domain growth into the existing 1D simulations within the ReactionDiffusion.jl framework. This would further bolster conclusions regarding robustness.

2) ReactionDiffusion.jl software
a) To quantify and justify claims about computational performance in the manuscript, it would be useful to perform benchmarking of ReactionDiffusion.jl compared to other existing tools.
b) To substantiate "able to screen millions of parameter sets per minute" and aid users in planning screens, it would further be useful to provide the details of one specific setup in terms of problem size / simulation task and computer configuration.

3) Data presentation
a) I suggest to rephrase overly strong statements in the abstract and title. For example, the title appears too general, although many important factors have not been included in the study (mechanics, delays, reaction-diffusion systems beyond activators and inhibitors etc.).
b) I suggest to adjust the abstract to better reflect the limitations of the study. For example, "to directly capture the mechanisms involved in intercellular signaling" does not seem appropriate given that modeling of some inhibition mechanisms is currently not directly derived from law of mass action kinetics.
c) For the abstract, I also suggest to give more credit to previous exhaustive studies that have analyzed multi-node Turing models with a separation of extracellular (diffusible) and intracellular (non-diffusible) compartments. This also applies to the introduction paragraph in lines 48-55. Similarly, I suggest to rephrase the introduction paragraph starting with "Rather than all behaving similarly - as implicitly assumed in simple 2-component Turing models", which has already been found by previous work published over the last 10 years.
d) I recommend to rephrase a few statements in the manuscript that overemphasize "realistic". The authors acknowledge that pre-patterns and more complex boundary conditions are not considered, which limits the aspect of more realistic systems (e.g. Haupaix et al. 2018, Singh et al. 2014). In addition, many known aspects crucial for signaling systems (e.g. the involvement of Type II receptors in Fig. 1B) are not captured by the current models.
r) Please explicitly mention in the text or figure legend what the special non-arrow connectors mean in Fig. 4 and related schematics (i.e. the effective influence of the intracellular black box).
f) In Fig. S5, is the range really from 0-1%, or is this rather the fraction ranging from 0-100%? The legend is ambiguous in that respect and states both "fraction" and "%".
g) The code is great and user-friendly, but a few minor typos in the tutorial (e.g. switched comma and semicolon for parameters) should be corrected.

---

**First revision**

Author response to reviewers' comments

Our response

Quoted text from revised manuscript

**Reviewer 1:**

This manuscript investigates Turing-type pattern formation in reaction-diffusion systems incorporating nondiffusible intracellular species, modeled under the assumption of fast equilibration relative to extracellular dynamics. By applying a quasi-steady-state (QSS) approximation to eliminate nondiffusible nodes, the authors derive a reduced system involving fewer species. They then employ classical linear stability analysis, partitioning the stability matrix to assess pattern-forming instabilities, and explore parameter regimes and pattern robustness using numerical simulations implemented via a dedicated Julia package, ReactionDiffusion.jl. The study is methodologically robust, with a well-structured analytical

© 2026. Published by The Company of Biologists under the terms of the Creative Commons Attribution License (https://creativecommons.org/licenses/by/4.0/).

framework and a flexible, well-documented computational tool. However, its biological insights are limited, and its theoretical contributions primarily reaffirm established principles without significantly advancing the understanding of pattern formation in developmental biology. Below, I elaborate on these points, highlighting the weaknesses and specific strengths that could boost novelty.

We thank the reviewer for their comments, and are encouraged by their evaluation of our study as "methodologically robust" and our computational tool to be "flexible, well-documented". We however do not agree that our findings "primarily reaffirm established principles", and have invested considerable time in improving the manuscript to better articulate our novel contributions and their biological significance. We respond to each point in turn below.

1 - Biological relevance remains underdeveloped
The manuscript investigates activator-inhibitor circuits within reaction-diffusion systems, focusing on how extracellular interactions constrain pattern-forming regulatory logic (lines 269-300, Figure 4). It references biologically inspired mechanisms, such as ligand-inhibitor interactions (e.g., WNT/sFRP in cortical organoids, Takata et al., 2017; GDF5/NOG in finger joint development, Grall et al., 2024), but does not anchor these generic network motifs to specific developmental processes or signaling pathways in a detailed manner. For example, the study identifies eight distinct classes of pattern-forming circuits (lines 327-328) based on phase relationships (e.g., in-phase or out-of-phase expression, Figure 5A-D), yet it does not map these motifs to well-characterized morphogenetic contexts, limb patterning, rugae or hair follicle formation.

The supplementary material provides mathematical derivations, including partial differential equations (PDEs) for receptor-inhibitor dynamics, but lacks any attempt to link these models to biological measurements, such as spatial gene expression profiles or protein concentration observed in vivo.

This absence of direct comparison with experimental data or specific developmental systems limits the study's ability to generate testable hypotheses or provide mechanistic insights into developmental processes. For a journal like Development, which prioritizes studies with strong biological grounding and experimental relevance, this lack of specific application significantly diminishes the manuscript's impact. To address this shortcoming, the authors could select a well-studied developmental system, such as BMP- Chordin signaling in Xenopus dorsal-ventral patterning (Zhu et al., 2023), and use their framework to simulate patterns, comparing outputs to experimental data to validate predictions and demonstrate biological relevance. Such an approach would strengthen the manuscript's alignment with the journal's focus on advancing developmental biology.

We welcome this suggestion, and acknowledge that in our enthusiasm for the mathematical methods and general insights we found, we may not have sufficiently highlighted what the implications were of these insights for biological systems.

To address this, we have explicitly referenced key periodic patterning systems in the context of our results in the discussion section, including those suggested by the reviewer:

These include the classic Turing-type logic, in which signalling promotes the simultaneous expression of both activator and inhibitor, as observed for example with WNT/DKK in hair follicle patterning (Sick et al., 2006) and Nodal/Lefty in early embryogenesis (Müller et al., 2012). However, several other circuits break symmetry but have an inverse relationship between signalling and activator/inhibitor production. Indeed, recent experimental observations point to numerous examples of this inverted logic operating in vivo, including GDF5/NOG in the developing joints (Grall et al., 2024) and TGFβ2/Fst in the optic cup (Grocott et al., 2020)…

Finally, when multiple signalling pathways are operating, we predict a variety of possible expression patterns, as observed and modelled across several in vivo systems, including patterning of the digits (Raspopovic et al., 2014), or the fingerprints (Glover et al., 2023)…

© 2026. Published by The Company of Biologists under the terms of the Creative Commons Attribution License (https://creativecommons.org/licenses/by/4.0/).

Whilst some in vivo patterns do appear to involve single activator/inhibitor pairs (e.g., GDF5/NOG in the developing finger joints (Grall et al., 2024)), others are suggested to involve multiple signals and pathways (e.g., patterning of the rugae (Economou et al., 2021, 2012), or murine hair follicles (Glover et al., 2017)).

We do not have the space to discuss each of these in vivo systems in any great detail. Moreover, we consider the main contributions/findings of this work (namely: insensitivity of symmetry breaking to intracellular dynamics; distinct A-I circuits based on extracellular behaviour; performant modelling framework) to be broadly applicable and we do not wish to tie them to any specific system.

We do, however, acknowledge the reviewer's suggestion that some more in-depth case studies would be helpful to illustrate how the general principles we found may be mapped to specific experimental contexts. Therefore - and after discussion with the editor - we decided to add **two new "boxes" that provide a more biological exposition of our results.** We focus on what we consider to be the two most biologically significant findings of our work.

**In Box 1** (and accompanying new Figures Box Fig 1 and Fig S6), we discuss the insensitivity of symmetry breaking to intracellular dynamics, focusing on WNT-BMP systems:

***Example 1: Symmetry breaking without a short-range activator and long-range inhibitor***

To illustrate how $\mathcal{N}$ may be applied to understand symmetry breaking in vivo, we now consider one example activator-inhibitor model in more detail, specifically one that comprises two distinct signalling pathways, WNT and BMP, in which WNT assumes the role of the Turing-like activator (i.e., undergoes positive feedback), and BMP acts as the Turing-like inhibitor. Indeed, there is experimental support for WNT-BMP circuits being involved in symmetry breaking in a variety of tissues, for example in the developing digits (Raspopovic et al., 2014) and fingerprint ridges (Glover et al., 2023). Previous theoretical work suggested constraints on the diffusive range of these signals for symmetry breaking to occur. In classic two-component Turing models (Gierer and Meinhardt, 1972), the diffusivity of the BMP ligand must be strictly larger than the diffusivity of the WNT ligand (Figure B1A). However, it was found that adding a single intracellular component to the models alleviated the requirement for differential diffusivity (Marcon et al., 2016), but the overall diffusive range of BMP (defined by both the diffusivity and extracellular half-life, $l_{BMP} \propto \sqrt{D_{BMP}\tau_{BMP}}$) must still be higher than the overall diffusive range of WNT (Figure B1B). Based on the mathematical insights provided by $\mathcal{N}$, we now considered how these constraints might change when adding further intracellular components to the models (see Supplementary information for an expanded discussion). Initially, we incorporated just two intracellular components, one to represent WNT signal transduction (i.e., ßCAT) and the other to represent BMP signal transduction (i.e., pSMAD). We predict that symmetry breaking is now possible even if WNT and BMP have identical diffusive ranges (i.e., same diffusivity and half-lives), provided there is sufficiently strong positive feedback on WNT signalling (Figure B1C). Thus, it seems that progressively adding intracellular components to the models reduces the constraints on the diffusive ranges of WNT and BMP, consistent with previous theoretical arguments (Marcon et al., 2016). One might imagine, therefore, that adding even more intracellular components would reduce further the requirements on WNT and BMP to break symmetry; indeed, a more detailed 5-component model of WNT-BMP interactions in the developing digits was reported to be highly robust to parameters (Marcon et al., 2016). However, we have shown, rather generally, that the limits derived from $\mathcal{N}$ for a WNT-BMP system with just two intracellular components (one per signalling pathway) are exactly equivalent to those derived for WNT-BMP models with any more complex intracellular scheme (Figure B1D). Therefore, whilst the requirements of differential diffusivity (Figure B1A) and differential diffusive range (Figure B1B) are not fundamental but instead a result of model oversimplification, the requirement for strong positive feedback (Figure B1C) is a general limit that must apply for all WNT-BMP systems of this type no matter how complex the intracellular circuitry is. Finally, we note that this property is not universal to all types of activator-inhibitor mechanism - for example, as outlined in the Supplementary information, a WNT-DKK circuit must always have differential diffusive range between WNT and DKK for symmetry breaking to occur.

© 2026. Published by The Company of Biologists under the terms of the Creative Commons Attribution License (https://creativecommons.org/licenses/by/4.0/).

**In Box 2** (and accompanying new figures Box Fig 2 and Fig S7), we propose WNT-sFRP as an activator- inhibitor pair, and highlight how the extracellular interactions between WNT and sFRP allow the system to break symmetry with a regulatory logic (and resulting expression patterns) not typically associated with Turing-like activator-inhibitor systems. [We consider this to be the most compelling/interesting biological example to present, and indeed note that the reviewer suggests WNT-sFRP as a suitable case study later in their comments]:

***Example 2: A putative WNT-sFRP circuit that can break symmetry***

Many of the symmetry-breaking motifs in our atlas (Figure 5) are present in activator-inhibitor circuits known to be involved in periodic patterning (see Discussion). However, the regulatory logic and resulting expression patterns associated with one circuit, namely that in Figure 5C1, represents a previously unrecognized activator-inhibitor mechanism capable of symmetry breaking. We hypothesize that this type of circuit may be implemented by WNT ligands and their diffusible inhibitors, sFRPs. Consistent with the circuit in Figure 5C1, sFRPs are known to be highly diffusive (Mii and Taira, 2009), they inhibit WNT ligands by directly binding to them but this binding is reversible allowing WNT ligands to be released from the WNT- sFRP complex (Mii and Taira, 2011). Moreover, WNT and sFRP expression domains are often observed to be out-of-phase with one another, for example in early embryos (Yamaguchi, 2001; Yamamoto et al., 2022), regenerating flatworms (Gurley et al., 2010) and symmetry-breaking cortical organoids (Takata et al., 2017).
Traditionally, this type of pattern – in which the domains of ligand and inhibitor expression are opposed to each other – is not associated with symmetry breaking, but rather in shaping morphogen gradients; for example, operating as an expander-repressor type circuit. Indeed, all other mechanisms in our atlas require ligand and inhibitor to be co-expressed in the same domain for symmetry breaking to occur (excluding the case of multiple signalling pathways). However, to explore the possibility that the observed antiphasic WNT-sFRP patterns may self-organize de novo, we simulated the dynamics of a WNT-sFRP circuit in which WNT signalling promotes WNT ligand expression and inhibits sFRP expression (Figure B2, see Figure S7 for code). Using parameter values informed by previous studies (Mii et al., 2021; Wawrzak et al., 2007; Yamamoto et al., 2022), we find that this mechanism can reliably break symmetry *in silico* to form patterns over biologically plausible time- and length-scales. Moreover, we find that if sFRP enhances the diffusivity of WNT when in complex – which has been confirmed in vivo (Mii and Taira, 2009) – then this significantly increases the likelihood that the system will break symmetry (Figure S5C). This aligns with the concept of ligand shuttling (Shilo et al., 2013), which is typically associated with regulation of morphogen gradients (e.g., gradient scaling); we suggest that another role for ligand shuttling may be to allow the circuit to break symmetry. Together, our results identify WNT-sFRP as a novel circuit that can break symmetry *in silico*; further experimental studies will be required to evaluate to what extent it is involved in symmetry breaking *in vivo*.

We thank the reviewer raising this point, and very much hope that these substantial additions help to articulate how the theory that we have done may be directly useful for understanding specific symmetry breaking phenomena in vivo.

<u>2 - The network structures and associated phase behaviors have been explored previously</u>

and

<u>6 - Literature engagement could be deepened</u>

We have grouped comments #2&#6 together as there is significant overlap, and our responses to each point are interrelated. We copy both sets of comments in full and provide our responses below.

The manuscript examines reaction-diffusion systems composed of two diffusible species (activator and inhibitor) and one or two nondiffusible intracellular species, analyzing their capacity to generate self- organized patterns with diverse phase relationships, such as co-expression (in-phase), anticorrelation (out- of-phase), or uniform expression of components (Figure 5, lines 301-314). These network architectures, particularly those involving nondiffusible nodes corresponding to signaling components, have been extensively studied in prior work, including Diego et al.

© 2026. Published by The Company of Biologists under the terms of the Creative Commons Attribution License (https://creativecommons.org/licenses/by/4.0/). 

(2018), Scholes et al. (2019), and Marcon et al. (2016). Notably, Marcon et al. (2016) analyzed three- and four-node networks with nondiffusible nodes representing signaling pathways, demonstrating that such architectures expand the repertoire of spatial phase relationships and enhance pattern robustness to parameter variations. Similarly, Diego et al. (2018) employed QSS reductions to simplify systems with nondiffusible components, deriving instability conditions that partition the stability matrix into diffusible and non difussible modules aligning with the manuscript's approach (Supplementary, Section 2.2). Scholes et al. (2019) further explored how network topology influences Turing pattern formation, identifying phase behaviors comparable to those cataloged in the manuscript's atlas (Figure 5A-D).

The current manuscript builds on this framework by simulating more complex dynamical realizations of these network motifs. While this is potentially useful, the resulting patterns and behaviors are consistent with what earlier theoretical analyses already predicted based on linearized systems. In particular, the emergence of diverse in-phase and out-of-phase profiles across species as a function of topology and diffusion ratios has already been established. The numerical simulations conducted using ReactionDiffusion.jl confirm these established patterns, such as in-phase co-expression or out-of-phase anticorrelation (Figure 5), but do not venture into novel dynamical regimes that may arise by the dimerization dynamics.

To enhance its contribution, the authors could explore uncharted dynamical regimes, such as those involving multistability that could give rise to more complex spatial patterns. In this way, their framework would generate unique predictions that extend beyond existing literature.

6 - Literature engagement could be deepened

The manuscript cites key prior studies, including Diego et al. (2018), Scholes et al. (2019), and Marcon et al. (2016), which analyzed Turing pattern formation in reaction-diffusion systems with nondiffusible nodes corresponding to signaling components (lines 376-378). Marcon et al. (2016) specifically explored three- and four-node networks with nondiffusible signaling nodes, demonstrating how such architectures enhance pattern robustness and diversify phase relationships, while Diego et al. (2018) used QSS reductions and stability matrix partitioning to derive instability conditions, mirroring the manuscript's methodology (Supplementary, Section 3.2.4). Scholes et al. (2019) further established how network topology determines robustness and spatial phase arrangements, aligning with the manuscript's atlas of patterns (Figure 5). However, the manuscript does not sufficiently engage with these works to clarify how its findings extend or diverge from their results. For example, the classification of eight pattern-forming circuits (lines 327-328) overlaps with phase behaviors identified in Marcon et al. (2016) and Scholes et al. (2019), but the manuscript does not discuss these similarities or articulate unique contributions, such as novel circuit types or parameter regimes driven by extracellular interactions.

The lack of deep engagement with these prior works obscures the manuscript's novelty and limits its scholarly impact. To address this, the authors should provide a comprehensive comparison with Diego et al., Scholes et al., Marcon et al., and Zhang et al., explicitly highlighting how their analysis advances the field, such as through new insights into extracellular inhibitor mechanisms or specific pattern-forming circuits not previously identified.

We appreciate the suggestion to more deeply engage with prior results from the literature, and hope that by more specifically describing what has been done before, we will also more specifically showcase the novel contributions that we have made in this study – thus addressing both comments #2 and #6.

In general terms, we have made two types of edit along these lines. The first is to expand our discussion of key published papers on this topic (including those suggested: Diego et al., Scholes et al., Marcon et al., and Zhang et al.). We do this in several places throughout the manuscript, including a significantly expanded section of the introduction, and a new paragraph in the Discussion. The second type of edit was to more clearly articulate the novel contribution that we have made in this paper.

We group these changes into the following themes:

© 2026. Published by The Company of Biologists under the terms of the Creative Commons Attribution License (https://creativecommons.org/licenses/by/4.0/).

### A. Complexity of intracellular interactions

A key distinction between our results and those considered in prior computational screens (like Diego et al., Scholes et al., Marcon et al., and Zhang et al.) is in the complexity of intracellular interactions (i.e., number of non-diffusible variables) considered. We do not, as the reviewer states, only consider "systems composed of two diffusible species (activator and inhibitor) and **one or two** nondiffusible intracellular species". For example, the model in Figure 1C contains **11** nondiffusible intracellular species; each of the models in Figure 3C, 3D, 3E, S2B, S2C, S2D include **more than two** nondiffusible intracellular species. More fundamentally, our mathematical approach (via the necessary condition N) generalizes to an **arbitrarily large number** of nondiffusible intracellular species, and thus the general limits we derive (i.e., the blue lines in Fig 4) implicitly accommodate models with significantly more than two nondiffusible intracellular species.

We consider this to be a major advance of this study, and we may not have made this sufficiently clear in our first submission. To address this, we begin by more deeply engaging with the literature on this general point – because, as the reviewer suggests, one of the important findings of previous computational screens (like Diego et al., Scholes et al., Marcon et al., and Zhang et al.) is the importance of the nondiffusible species. In many ways, this observation is the starting point and motivation for us to consider systems with more than 2 nondiffusible species, since we did not know a priori to what extent these more complex intracellular networks may further challenge our understanding of Turing-like patterning. Indeed we were surprised to be able to prove some general limits for arbitrarily complex intracellular networks.

To better articulate this, we have significantly expanded the introduction to emphasize how previous papers have demonstrated the importance of nondiffusible species in reaction-diffusion patterning, and then use this to motivate us to consider more complex intracellular networks:

Moreover, a general theme that has emerged is that factors that do not diffuse between cells (e.g., transcription factors, signal transduction elements) can significantly impact the ability of the system to break symmetry (Diego et al., 2018a; Marcon et al., 2016; Scholes et al., 2019). However, given the large number of these components and their possible interactions, previous models have necessarily underestimated the intracellular complexity present *in vivo*, and it remains unclear what impact this simplification will have on model predictions

To illustrate more concretely how our results on more complex intracellular dynamics are relevant to biology, we have focused on a specific system in Box 1, again being sure to highlight previous work in this area and articulate our novel contribution.

### B. Biochemical detail of model equations

A second key difference between our study and previous computational screens is in the specific model equations used. Some start directly with linear models (like Diego et al., Marcon et al.), whereas others using hill-type functions to denote regulatory coupling (like Scholes et al., and Zhang et al.). In contrast, we use mass action kinetics to describe the dynamics of known biochemical interactions, and arbitrary functions to denote aggregated intracellular response functions (in simulations we use hill functions for these terms, but our general limits on the sensitivities, H, do not depend on the precise functional forms chosen).

Importantly, these mathematical differences matter! This is true even when studying the linearized dynamics of our models, because the nonlinear terms implicitly define the linearized coefficients. This is perhaps easiest to illustrate with an example, which is rather simple/trivial, but we hope makes explicit and concrete the point we are trying to make.

*Example:*

Consider a hypothetical system in which two diffusible molecules, A and B, reversibly form a complex, C; and that A and B are produced at a steady rate, and all molecules degrade at a certain rate (degradation rates can be different to one another). If we

© 2026. Published by The Company of Biologists under the terms of the Creative Commons Attribution License (https://creativecommons.org/licenses/by/4.0/).

write mass action kinetics for this system, this results in the following PDEs:

$$\frac{\partial A}{\partial t} = \lambda_A - k_+ AB + k_- C - \delta_A A + D_A \nabla^2 A$$

$$\frac{\partial B}{\partial t} = \lambda_B \quad - k_+ AB + k_- C - \delta_B B + D_B \nabla^2 B$$

$$\frac{\partial C}{\partial t} = k_+ AB - k_- C - \delta_C C + D_C \nabla^2 C$$

Model 1

The resulting Jacobian for the reaction terms therefore has the sign structure:

$$\begin{pmatrix} - & - & + \\ - & - & + \\ + & + & - \end{pmatrix}$$

i.e., is represented by a graph with the following network interactions (ignoring self-loops for simplicity):

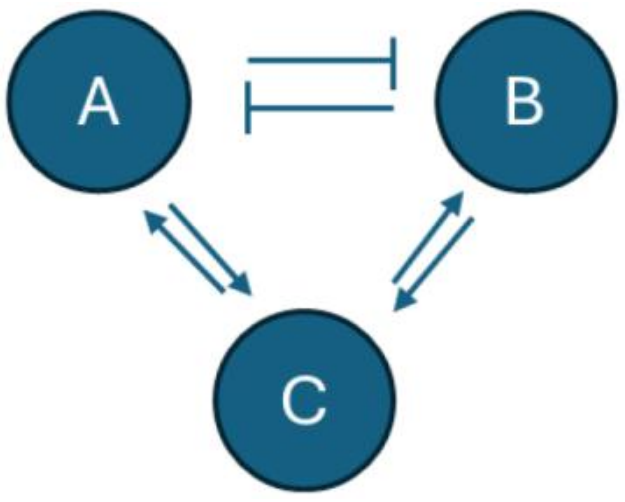


Now, if we started directly from this graph/Jacobian sign structure/network topology, and allowed linear/hill-function coupling based on these interactions – with the parameters of each coupling term allowed to vary freely – then in general this would not be identical to the linearized behaviour of Model 1. This is because the equations of model 1 not only determine the sign structure of the Jacobian, they also constrain the specific values and these in turn impact the calculation of the Turing-instability criterion. Thus the biochemical models we use (like the simple example of model 1) are more specific – but equally, less general – than methods based on interaction networks.

We have highlighted this distinction in the introduction:

Moreover, whilst containing more than 2 elements, the models are still simplified network-level abstractions which approximate molecular interactions by simple mathematical expressions (e.g., linear or hill functions), rather than directly capturing the nature of developmental signalling *in vivo*. One strategy has been to assume sigmoidal/hill-type interactions between each component (Scholes et al., 2019; Zheng et al., 2016); this has the advantage of naturally capturing the saturating nature of many biological feedbacks, but poorly reflects more direct biochemical interactions (for example, binding of an inhibitor to an activator molecule in the extracellular space). Another approach is to instead consider linear coupling between each component, facilitating a comprehensive algebraic treatment (Diego et al., 2018a; Marcon et al., 2016). However, the assumption of linearity neglects the intrinsically nonlinear character of most biological interactions (even when considering linearized dynamics since the nonlinearities

© 2026. Published by The Company of Biologists under the terms of the Creative Commons Attribution License (https://creativecommons.org/licenses/by/4.0/). 

themselves dictate the allowed values of any linearized coefficients). Therefore, whilst these approximations have allowed systematic analyses of reaction-diffusion circuits *in silico* – which has pointed to important guiding principles – it remains unclear to what extent their simplifying assumptions will impact the applicability of model predictions *in vivo*.

Further, we do not wish to imply that employing mass action kinetics, like we do, is always the best option – sometimes it may be more appropriate to use linear or hill-function coupling. In the Discussion, we have therefore discussed the advantages of linear or hill-function coupling (e.g., more natural way to perform an unbiased screen focusing on network topology; facilitates algebraic analysis; easier to look at models with more than 2 *diffusible* components):

More generally, there are instances where a topology/network-based approach may be more instructive – for example, performing fast algebraic analysis of linear models with RDNets (Marcon et al., 2016) or numerical analysis of generic network architectures (Scholes et al., 2019; Zheng et al., 2016) – but in other cases, a more explicitly mechanistic model, as we have explored here, may be more informative.

**C. Phase relationships**

We certainly agree that a "diverse in-phase and out-of-phase profiles across species as a function of topology and diffusion ratios has already been established". We acknowledge that this may not have been clearly articulated in our initial submission, and have modified text in the introduction accordingly:

These analyses reveal that many types of periodic pattern can form with a variety of phase relationships possible amongst the different model components.

However, whilst *in general* it is well established that many in-phase/out-of-phase expression are possible, we have predicted what those specific phase relationships are for the specific 1-activator, 1-inhibitor systems we have considered [which, as described in point B above, are not trivially equivalent to models with similar *network topologies* but different nonlinear terms]. The key difference is therefore not relating the phase relationships to network topology (as has been done systematically in e.g., Diego et al., Scholes et al., Marcon et al., including for models with more than 2 diffusible species), but rather relating the phase differences to the type of *extracellular inhibitor* present.

We have tried to highlight this distinction with an additional concrete example in the results section:

To give a concrete example, consider activator-inhibitor circuits based on the WNT signalling pathway, with two common families of inhibitors: DKKs and sFRPs. Whilst DKKs and sFRPs are both inhibitors of WNT signalling, their (extracellular) mode of action is rather different; DKKs bind to LRP5/6 co-receptors preventing formation of an activated receptor complex, whereas sFRPs inhibit WNT ligands by binding to them directly. Our analysis shows that this results in qualitative differences (i.e., distinct regulatory circuits capable of symmetry breaking) when comparing WNT-DKK and WNT-sFRP systems.

**D. Computational atlas of activator-inhibitor patterns**

Our hope in the computational atlas of Figure 5 was to provide a unifying/complete description of all 1- activator, 1-inhibitor circuits that can break symmetry. We acknowledge that some of the motifs are consistent with topologies identified previously (like Diego et al., Scholes et al., Marcon et al., and Zhang et al.) but again refer to point B to emphasize that this is not a trivial result given the difference between the nonlinear model components. To highlight this, in the Discussion we have referred to the areas of commonality between our computational atlas and network topologies proposed previously:

We highlight that our computational atlas of activator-inhibitor motifs naturally complements results from existing in silico screens. Whereas these previous studies systematically varied the topology of interaction networks – assuming coupling via linear or hill functions – we instead took a

© 2026. Published by The Company of Biologists under the terms of the Creative Commons Attribution License (https://creativecommons.org/licenses/by/4.0/).

more targeted approach by considering specific molecular mechanisms associated with signalling. Our mathematical results (i.e., the limits derived from N) provide a rigorous basis for classifying circuits based purely on the type of extracellular inhibitor involved. Some of the circuits we describe can be mapped to topologies identified in previous high-throughput screens (e.g., the type II networks analysed in (Marcon et al., 2016)), however we have also uncovered mechanisms that had not previously been associated with symmetry breaking (see Box 2).

However, we would like to emphasize that our approach is different in two important ways from previous screens. Firstly, we are systematically varying extracellular inhibitor type, not network topology; we consider there to be complementary value in both types of systematic approach. Indeed, by focusing on the inhibitor type, this pointed us towards considering an inhibitor which reversibly forms a complex with a signalling ligand, thus adding a new diffusible element to our model – and, as we describe in Box 2 through the lens of WNT-sFRP, this led us to hypothesize a circuit that has not typically been associated with symmetry breaking. Thus, whilst our computational screen recovered many known topologies, it also pointed us to a circuit that we were not expecting.

Secondly, in contrast to previous work, we have derived general limits (via N) for each of our proposed extracellular inhibitor type, and therefore our atlas extends beyond systems with only one or two nondiffusible elements, thus providing a rigorous basis to classify mechanisms based on the extracellular inhibitor type alone.

**Together**, we hope that the substantial edits we have made to the main text better articulate the both: (i) the results from prior literature, (ii) novel contributions made in this manuscript.

[For completeness, there are two specific comments in points #2&#6 that we have chosen to address elsewhere as they overlap with other comments, specifically:

explore uncharted dynamical regimes, such as those involving multistability that could give rise to more complex spatial patterns We respond to this point more fully in comment #4 below.

Similarly, Diego et al. (2018) employed QSS reductions to simplify systems with nondiffusible components patterns We respond to this point more fully in comment #3 below.]

<u>3 - The QSS-derived condition is mathematically valid but closely aligned with classical results</u>
The manuscript's core analytical contribution is the derivation of a necessary condition (N) for pattern formation, achieved through a quasi-steady-state (QSS) approximation that eliminates nondiffusible species, such as receptors and receptor-inhibitor complexes, to produce a reduced system of diffusible species (Supplementary, Section 2). The stability matrix is partitioned into diffusible and nondiffusible components, and the Schur complement approach is used to compute the reduced Jacobian matrix (Equation 23), which is analyzed via classical linear stability criteria to identify Turing instabilities. This partition of the stability matrix, separating diffusible and nondiffusible dynamics, is mathematically rigorous but closely mirrors the methodology employed in Diego et al. (2018), where a similar block decomposition was used to derive instability conditions for reaction-diffusion systems with nondiffusible signaling components. The manuscript explicitly notes that the constraints derived from N depend solely on the type of extracellular inhibitor and are independent of intracellular dynamics (lines 298-300), aligning with classical Turing theory's focus on diffusion-driven instabilities. While the derivation is technically sound, it does not introduce fundamentally new mechanisms, effectively re-expressing established linear stability conditions for reduced systems. The assumption that nondiffusible components rapidly reach a unique, stable steady state is standard but limits the analysis to linear regimes.

We thank the reviewer for these comments, which describe – to us – the most exciting part of our results, namely the ability to derive *general yet exact conclusions from simple/reduced* models that assume the QSS approximation. We want to emphasize that our results are not approximate nor do they rely on the QSS assumption, but rather that general limits (i.e., N) can be derived *exactly* by considering a reduced system in which we do assume that intracellular

© 2026. Published by The Company of Biologists under the terms of the Creative Commons Attribution License (https://creativecommons.org/licenses/by/4.0/).

species reach quasi-steady-state. This was a very counterintuitive result to us, but one that has been thoroughly tested with our numerical simulations and supported via mathematical proof.

However, based on the above reviewer comments, it is clear that we had failed to accurately communicate these findings in our first submission, and therefore we have added an entire new paragraph to the main text which attempts to explain the generality of our results more explicitly:

We emphasize that these limits are not approximate nor do they rely on any separation of timescales between intracellular and extracellular components. Instead, the necessary condition N of systems with arbitrarily complex intracellular dynamics (including oscillations, time averaging, other temporal filtering) may, counterintuitively, be determined exactly without explicitly considering these dynamics (see Supplementary information). This does not imply that these dynamics will have no impact on patterning, but rather that they do not affect the necessary condition N, and thus the ability of the circuit to break symmetry. Therefore, there exist general limits on symmetry breaking that will be broadly applicable regardless of the precise intracellular feedback mechanisms – or intracellular parameters – at play (Figure 2C). This result is unexpected, since prior work highlighted the importance of considering non-diffusible species in reaction-diffusion circuits (Diego et al., 2018a; Marcon et al., 2016), but we find that a single non-diffusible component per signalling pathway is sufficient to compute N.

Moreover, we have tried to explain the mathematical intuition underpinning these results in the supplementary information.

A loose intuition underpinning this result may be gained by considering the definition of $\mathcal{N}$, which states that $|\mathbf{J} - q^2\mathbf{D}|$ must change sign twice for some positive (and finite) values of $q$ for symmetry breaking to be possible. This means that there must be two values of the wavevector, $q_1, q_2$, for which this determinant (and therefore the relevant eigenvalue) must be exactly zero (i.e., $|\mathbf{J} - q_1^2\mathbf{D}| = 0$ and $|\mathbf{J} - q_2^2\mathbf{D}| = 0$). For example, at $q = q_1$ and infinitesimally above it, the instability will thus have an eigenvalue with an infinitesimally small real part i.e., it will exhibit very slow dynamics. As $q \to q_1^+$, these dynamics will thus become infinitely slow and at some point will be slow enough that all intracellular dynamics will reach a quasi-steady state over this timescale. Now, it is important to note that this argument only applies at values of the wavevector with zero growth rates (i.e., $q_1, q_2$), and that between these values, the intracellular interactions will impact the resulting dynamics and eventual pattern formed. However, since $\mathcal{N}$ is determined by the determinant sign change (i.e., precisely by the points $q_1, q_2$), this means that the intracellular dynamics do not impact the computation of $\mathcal{N}$. Following the same arguments, we also conjecture that time delays in the intracellular compartment may also be neglected when computing $\mathcal{N}$, but we have not formally proved this or demonstrated its validity numerically.

Finally, we have included a biological case study (Box 1), in which we aim to describe the biological implications of these results.

We hope that these major edits have clarified the comments raised above.

For completeness, we now also respond to the individual comments:

This partition of the stability matrix, separating diffusible and nondiffusible dynamics, is mathematically rigorous but closely mirrors the methodology employed in Diego et al. (2018), where a similar block decomposition was used to derive instability conditions for reaction-diffusion systems with nondiffusible signaling components.

Whilst our approach takes inspiration from the methods used in Diego et al. (2018), our novelty is to explicitly derive the necessary condition N and show that it is preserved under the QSS approximation even for more complex intracellular dynamics. Having thoroughly read Diego et al. (2018), we still cannot find where block decomposition/Schur's lemma is explicitly used (although some of the algebraic steps may implicitly use it without stating it), and there is no mention made to connect the QSS regime and the simplification afforded by block decomposition/Schur's lemma. Instead, we primarily use the methods from Diego et al. (2018) to evaluate the determinant of the reaction-diffusion matrix as a polynomial in q. We do use – and clearly acknowledge in the main text – results from a different paper, Smith and Dalchau (2018), to draw a connection between the

© 2026. Published by The Company of Biologists under the terms of the Creative Commons Attribution License (https://creativecommons.org/licenses/by/4.0/).

QSS limit and our reduced Jacobian, allowing us to provide a biological/experimental interpretation of our reduced Jacobian. However, whilst drawing on the methodologies of both Diego et al. (2018) and Smith and Dalchau et al. (2018), our definition and evaluation of the necessary condition N; our explicit separation of extracellular, transmembrane, and intracellular components based on the structure of signalling pathways; the resulting simplification this implies for the Jacobian structure; our definition of a reduced yet experimentally-interpretable/measurable model; and our method to compute N exactly *without* considering the full intracellular dynamics are, to our knowledge, contributions that are novel to this manuscript. We hope that our above edits to the main text and supplement are sufficient to clarify this point.

The manuscript explicitly notes that the constraints derived from N depend solely on the type of extracellular inhibitor and are independent of intracellular dynamics (lines 298-300), aligning with classical Turing theory's focus on diffusion-driven instabilities.

Here we disagree. One of the surprising but important results from Marcon et al. 2016 and Diego et al. 2018 is that adding non-diffusible species to reaction-diffusion circuits can drastically change their behaviour (e.g., remove the requirement for differential diffusivity in activator-inhibitor models). It was not obvious to us before doing this work whether adding more intracellular dynamics (i.e., more non-diffusible species) would further change the properties of such systems. We were initially surprised to find that we could derive general limits (via N) that *did* not depend on the intracellular species, provided one includes at least one intracellular component per pathway. Thus, it seems that adding one intracellular component can give *qualitatively* different conclusions compared to classic Turing models (from Marcon et al 2016, Diego et al 2018), but that only one non-diffusible component per pathway is required to derive rather general constraints on symmetry breaking parameters.

To better highlight this point, we have included a new biological case study (Box 1), which shows how these results may apply to a concrete biological example. We also hope that this goes some way to addressing point #6 below, namely more specifically articulating our results in the context of prior work.

While the derivation is technically sound, it does not introduce fundamentally new mechanisms, effectively re-expressing established linear stability conditions for reduced systems.

Referencing our arguments and edits above, we highlight that the necessary condition N is not only valid for the reduced systems, but rather they may be derived from the reduced systems but apply rather generally to a diversity of intracellular dynamics. We consider this, by itself, to be a fundamentally new and important result, and hope that the edits to our manuscript adequately convey this. In terms of “fundamentally new mechanisms”, we also hope that our expanded description of Figure 5 and the additional case study (Box 2) clearly articulate where our approach has indeed revealed such a mechanism.

The assumption that nondiffusible components rapidly reach a unique, stable steady state is standard but limits the analysis to linear regimes.

We hope that our above arguments and edits to main text/supplementary outline why this assumption is not required for us to compute the necessary condition N. Moreover, the models we have considered (both algebraically and numerically) are all explicitly nonlinear, and our findings do not only apply to linear models. We expand upon this below in our response to point #4 which also touches upon the importance of nonlinear dynamics.

Nonlinear scenarios, such as bistability or oscillatory dynamics, are only briefly acknowledged in the context of combined degradation and dissociation of complexes (lines 291-295) without thorough investigation. This omission is significant, as nonlinear dynamics could reveal novel pattern-forming mechanisms relevant to developmental biology. To strengthen its theoretical novelty, the authors could analyze the impact of nonlinearities, such as multistability or bifurcations (as explained below), or assess the robustness of the QSS approximation under conditions where the steady-state assumption fails, thereby extending the framework beyond classical results.

© 2026. Published by The Company of Biologists under the terms of the Creative Commons Attribution License (https://creativecommons.org/licenses/by/4.0/).

We acknowledge these comments on nonlinearities and multistability, but since point #4 below deals with these arguments too, we have decided to collate our responses to both sets of comments and provide these below in our response to point #4.

4 - Dimerization introduces nonlinearity, but its dynamic implications are not explored
The manuscript incorporates dimerization-like reactions, such as the formation of ligand-inhibitor complexes that either degrade irreversibly (Figure 4A, lines 269-274) or dissociate reversibly (Figure 4C, lines 278-285), introducing nonlinear interactions into the reaction-diffusion system. These nonlinearities are evident in the PDEs governing receptor-inhibitor dynamics (Supplementary, Equation 23, lines 310-311), which include binding (k+[I][R]) and dissociation (k−[C]) terms that could lead to multistability, bifurcations, or other complex dynamical behaviors.

Such phenomena are highly relevant to developmental biology, where switch-like behaviors and multiple attractors underpin processes like cell fate decisions and tissue compartmentalization (e.g., WNT signaling, Mii and Taira, 2011). However, the manuscript restricts its analysis to linear stability and QSS assumptions, focusing on the reduced system's Jacobian matrix (Supplementary, lines 311-314) without exploring how dimerization affects the number, stability, or spatial distribution of steady states. For instance, the interplay between binding and dissociation rates could produce multiple steady states, altering the applicability of the QSS reduction or leading to novel pattern types, such as localized domains or traveling waves.

The manuscript does not investigate these possibilities, nor does it assess whether the QSS approximation remains valid under conditions where dimerization induces complex dynamics. This oversight limits the study's ability to uncover new mechanistic insights into pattern formation. To address this, the authors could conduct a bifurcation analysis to examine how dimerization parameters (e.g., k+, k−, δC) influence steady- state multiplicity or pattern robustness, or simulate scenarios where nonlinear effects yield distinct patterns, such as those observed in developmental systems with bistable signaling pathways. Such an analysis would significantly enhance the manuscript's theoretical and biological relevance.

We begin by broadly responding to these comments as a group, before later addressing individual points specifically.

We certainly share the reviewer's enthusiasm to understand the roles that interesting nonlinear dynamics may play in developmental biology - including "multistability", "oscillatory dynamics", "localized domains or traveling waves". We think this an incredibly rich area of investigation, both mathematically and in the context of development, and indeed we have other exciting projects ongoing in the group that are along these directions. However, we consider that these phenomena are significantly outside of the central focus of this manuscript, specifically Turing-like symmetry breaking mechanisms, which are in their own right incredibly rich and developmentally-important phenomena. Moreover, the absence/presence of these nonlinear dynamics does not change the fact that the necessary condition N (i.e., the [linear] Turing-instability criterion) must still hold if an initially homogeneous pattern is able to break symmetry. In other words, N can reliably predict whether a system can *initially* break symmetry but does not predict what patterns will *finally* self-organize.

In our original manuscript, we acknowledge that we did not make this point sharply enough. We referred to general properties of "patterning" and "pattern-forming", when in fact our analysis focused exclusively on a much narrower phenomenon: initial symmetry breaking (i.e, Turing instabilities). By restricting our attention to symmetry breaking, this allowed us to make some rather general statements - and as we argue above, these include novel results that we hope are of significant interest to the developmental biology community. But we of course acknowledge that our focus on linear instability/symmetry breaking comes with its limitations - not that this contradicts the general limits we derived, but rather that we are choosing to neglect other nonlinear dynamics that could be playing relevant roles in some tissues.

We have made substantial alterations to the manuscript text to better articulate these points, and sharpen exactly what our results do and do not show.

© 2026. Published by The Company of Biologists under the terms of the Creative Commons Attribution License (https://creativecommons.org/licenses/by/4.0/).

Firstly, we have changed all mentions of “patterning” and “pattern-forming” to “symmetry breaking” (or occasionally “self-organize de novo” where it felt too repetitive to say “symmetry breaking”).

Secondly, we have been upfront early in the results section about our restriction to initial symmetry breaking (i.e., linear instability analysis) and our inability to say anything more about more complex nonlinear dynamics:

To achieve this, we focus on one property of reaction-diffusion systems in particular – their ability to break symmetry i.e., the very earliest step in self-organized patterning. Biologically, this corresponds to an initially uniform tissue spontaneously self-amplifying small stochastic fluctuations in gene expression/protein concentrations across a specific range of spatial wavelengths. The advantage of focusing on the symmetry breaking step is that, even for complex nonlinear systems, the conditions for symmetry breaking can be derived via linear stability analysis…

…We note that this linear instability analysis does not guarantee that a self-organized pattern will eventually form, but it is sufficient to determine whether a given reaction-diffusion system can break symmetry. In other words, N can predict whether a system can initially break symmetry but does not predict what patterns will finally self-organize.

Finally, we have expanded the discussion section with a paragraph explicitly highlighting the limitations of our linear stability approach, including referencing examples of specific cases where nonlinear dynamics likely play an important role:

It is important to note that an inherent limitation of our approach is that the necessary condition N applies only to phenomena which are genuinely symmetry breaking i.e., patterns that self-organize de novo within homogeneous tissues. The constraints we have derived are thus necessary for a circuit to break symmetry, but do not predict whether a stable self-organized pattern will eventually form, nor the properties of that pattern (e.g., the amplitude, shape, and spacing of each periodic repeat, or its dependence on tissue geometry (Woolley, 2025b, 2022)). For example, in the case of multistable reaction-diffusion systems, there can be an initial symmetry breaking from one of the stable states, but for some parameters the resulting periodic pattern is transient and eventually disappears to be replaced by an alternative (homogeneous) steady state (Krause et al., 2024; Scholes et al., 2019). Moreover, our analysis does not exclude the presence of other instabilities (for example, patterns may also self-organize via oscillatory dynamics (Oates et al., 2012), localized wavefronts (Rulands et al., 2013), mass-redistribution (Brauns et al., 2020) or wave- pinning type interactions (Mori et al., 2008)) and is formally only exact in the limit of low noise.
Nevertheless, provided we restrict our attention solely to whether a given mechanism can break symmetry, then N does provide strict, broadly applicable conditions that are necessary (but not sufficient) for symmetry breaking to occur.

We thank the reviewer for raising these comments and hope that we have significantly improved the precision with which we have articulated our questions and conclusions.

We shall now address specific points in turn.

The manuscript incorporates dimerization-like reactions, such as the formation of ligand-inhibitor complexes that either degrade irreversibly (Figure 4A, lines 269-274) or dissociate reversibly (Figure 4C, lines 278-285), introducing nonlinear interactions into the reaction-diffusion system. These nonlinearities are evident in the PDEs governing receptor-inhibitor dynamics (Supplementary, Equation 23, lines 310-311), which include binding ($k_+[I][R]$) and dissociation ($k_-[C]$) terms that could lead to multistability, bifurcations, or other complex dynamical behaviors.

We agree that the nonlinear PDEs we studied may exhibit “multistability, bifurcations or other complex dynamical behaviours”. But as we argue above, this does not alleviate the need for the system to satisfy the necessary condition N in order for it to break symmetry.

© 2026. Published by The Company of Biologists under the terms of the Creative Commons Attribution License (https://creativecommons.org/licenses/by/4.0/).

We do acknowledge that these dynamical behaviours may compromise the ability of the system to eventually form periodic patterns even if the necessary condition N is satisfied – and we have tried to clearly articulate this in our edits to the main text. However, we would like to note that we have performed numerical simulations of these nonlinear PDEs and already found a large number of parameter sets do self-organize into stable periodic patterns. We do not mean to exclude the possibility that more complex dynamics might be present for some regions of parameter space, but rather to convey that there does exist regions of parameter space for which our chosen nonlinear PDEs do eventually form stable periodic patterns, which for our purposes, suffices to provide concrete hypotheses for pattern-forming circuits.

Finally, we note that all of our models are nonlinear, not just the ones including dimerization reactions. The intracellular response functions (i.e., impact of signalling on gene expression) are all assumed to be nonlinear sigmoid functions.

Such phenomena are highly relevant to developmental biology, where switch-like behaviors and multiple attractors underpin processes like cell fate decisions and tissue compartmentalization (e.g., WNT signaling, Mii and Taira, 2011). However, the manuscript restricts its analysis to linear stability and QSS assumptions, focusing on the reduced system's Jacobian matrix (Supplementary, lines 311-314) without exploring how dimerization affects the number, stability, or spatial distribution of steady states. For instance, the interplay between binding and dissociation rates could produce multiple steady states, altering the applicability of the QSS reduction or leading to novel pattern types, such as localized domains or traveling waves.
The manuscript does not investigate these possibilities, nor does it assess whether the QSS approximation remains valid under conditions where dimerization induces complex dynamics. This oversight limits the study's ability to uncover new mechanistic insights into pattern formation.

As discussed above, we agree with the reviewer about the potential importance and significance of complex dynamics/travelling waves/localized domains in developmental patterning, but maintain that our focus on symmetry breaking still covers a rich and diverse array of developmental patterning phenomena. In other words, our manuscript provides developmentally-relevant hypotheses specific to symmetry breaking, but explicitly leaves open the possibility of more exotic reaction-diffusion dynamics in other systems. We hope that our edited main text has accurately conveyed this sentiment.

We would also like to refer back to our response to point #3 here, specifically that our results do not require the QSS assumption to hold and apply rather generally – and so the addition of nonlinearities like dimerization will not impact the necessary conditions that we derive. See also our response to the point below.

To address this, the authors could conduct a bifurcation analysis to examine how dimerization parameters (e.g., $k+$, $k-$, $\delta C$) influence steady-state multiplicity or pattern robustness, or simulate scenarios where nonlinear effects yield distinct patterns, such as those observed in developmental systems with bistable signaling pathways. Such an analysis would significantly enhance the manuscript's theoretical and biological relevance.

As argued above, our manuscript focus is on symmetry breaking, and – whilst certainly very interesting –we think that an in-depth study of nonlinear phenomena in patterning is outside of the scope of this article.

However, we acknowledge that bi/multistability is an area in particular that would benefit from more explanation in our text, and outline our edits below.

Firstly, as has been reported elsewhere (e.g., Scholes et al 2019, Krause et al 2024), in reaction-diffusion systems with multiple (homogeneous) steady states, it is possible that one of these steady states undergoes a diffusion-driven instability, but that the ensuing periodic pattern is only transient, and eventually the system settles into the (other) homogeneous steady state, which cannot undergo a diffusion- driven instability. We have discussed this possibility with the following additional sentence in the Discussion:

For example, in the case of multistable reaction-diffusion systems, there can be an initial

© 2026. Published by The Company of Biologists under the terms of the Creative Commons Attribution License (https://creativecommons.org/licenses/by/4.0/).

symmetry breaking from one of the stable states, but for some parameters the resulting periodic pattern is transient and eventually disappears to be replaced by an alternative (homogeneous) steady state (Krause et al., 2024; Scholes et al., 2019).

However, we would like to note that this possibility does not contradict our approach nor our findings. We focus our attention purely on the earliest symmetry breaking step and – as now detailed in our revised text
– this does allow us to derive necessary conditions for the initial symmetry breaking, but it does not guarantee that a stable periodic pattern will eventually form, since other possibilities may arise (including but not limited to the scenario where the system relaxes back to an alternative homogeneous steady state).

For cases in which there are multiple steady states, our definition of symmetry breaking must, strictly speaking, refer to the specific steady state from which symmetry breaking occurs – and therefore in multistable systems, it's possible that some steady states but not others can break symmetry. However, regardless of the steady state, our general limits (i.e., the algebraic inequalities derived in the Supplementary materials, an example of which is plotted in the blue lines in Figure 4) still apply. In particular, the normalized sensitivities (e.g., H_S->A, H_S->I) will have the same constraints and therefore same signs, and so the regulatory logic that we have identified in Figures 4&5 will hold regardless. For example, if we have an activator-inhibitor system where the inhibitor irreversibly binds the activator (i.e., in Figure 4A/5A), then it must be the case that signalling activates both activator and inhibitor for symmetry breaking to occur *about any possible steady state of the system, including where there are multiple steady states*. We have articulated this point in our revised supplementary materials:

### 1.3 Considerations for multistable reaction-diffusion systems

In the default mode of *ReactionDiffusion.jl*, only one steady state is explored per parameter set. However, for reaction-diffusion systems that exhibit multistability (i.e., there are more than one stable homogeneous steady state), then strictly speaking the condition for symmetry breaking will apply only to the specific steady state that is identified. For the models considered in this manuscript, this approach was sufficient to identify many symmetry-breaking parameter sets. We also emphasize that the necessary condition $\mathcal{N}$ (derived in the next section) applies regardless of the steady state, and therefore all the algebraic inequalities and general limits that we find do not depend on any assumption of monostability. However, to broaden the scope of its applicability, we here describe how to deal with steady state multiplicity in *ReactionDiffusion.jl*.

Now, a further point to consider is that steady state multiplicity raises the possibility that we may miss systems capable of breaking symmetry (i.e., there may be false negatives) because we did not choose the appropriate steady state to investigate. In general, this is a hard problem – for arbitrary nonlinear systems of ODEs, we do not know of a performant way to exhaustively find all (stable) steady states of the system with 100% certainty (there are approaches for specific choices of the nonlinearities e.g., polynomial functions, but we want our approach to be more general). A practical approach – mirroring that taken in Scholes et al 2019 – is to allow our algorithm to vary the initial condition when computing the steady states, thus allowing alternative steady states to be found. This is not guaranteed to find all steady states (since it itself depends on what initial states are used to seed the steady state solver), but it has been proposed and applied in Scholes et al 2019. We describe the consideration of multiple steady states in our expanded supplementary materials:

© 2026. Published by The Company of Biologists under the terms of the Creative Commons Attribution License (https://creativecommons.org/licenses/by/4.0/).

Ideally, one would want to be able to formally detect, with certainty, all stable steady states of a system. However, for an arbitrarily nonlinear system of ODEs, we are not aware of a performant method to exhaustively detect steady states. For some systems (e.g., those involving only polynomial functions), methods such as homotopy continuation may be employed. However, in *ReactionDiffusion.jl*, we take a more practical approach that is more general than polynomial functions, mirroring that which was employed in previous studies [4]. In particular, when detecting Turing-instabilities, we have allowed users to vary not just parameter values, but also the initial values used to seed the steady state solver. In this way, by varying these initial values, it is possible to find alternative steady states and consequently perform the same Turing-instability detection but about more than one steady state per parameter set. In *ReactionDiffusion.jl*, this is achieved by varying the initial values; for example, to vary the initial value of the gene GDF5 to a value of 0, 1, or 10 to detect Turing-instabilities in the model of Figure 1, one would write:

```
params.initial_condition["GDF5"] = [0; 1; 10]
```

We emphasize that for the specific models that we considered, we were not noticeably hampered by this type of false negative, even if it's possible that some of the parameter sets did indeed exhibit multistability. In particular, for each model we considered, we found a large number of parameter sets predicted to break symmetry, and these identified parameter sets were relatively close to the algebraic limits (i.e., close to the light blue lines in Figure 4), which was sufficient for us to demonstrate that the necessary conditions we derived were relatively tight. Moreover, across all models we considered, when we performed PDE simulations, we found many exemplar parameter sets that not only broke symmetry but also formed stable periodic patterns.

*Example:*

To illustrate these points in more detail, we follow the reviewer's suggestion to conduct a more in- depth analysis of a model involving dimerization. Specifically, to assess the impact of dimerization- induced nonlinearities and steady-state multiplicity, we analysed in detail the steady states of a model in which the inhibitor reversibly sequesters the activator into a complex (i.e., the model in Figure 4C/5C). In general, we do not know of a performant way to exhaustively characterize the steady states of an ODE system of arbitrary complexity. However, if we restrict ourselves to polynomial-type functions – including Hill functions with integer coefficients – then homotopy continuation methods may be employed to comprehensively catalogue steady states.

We therefore restricted our attention to intracellular response functions with integer hill function terms, scanning approx. 490,000 parameter sets (corresponding to the same ranges shown in Figure S5). We computed all steady states (and their stability) for each parameter set with HomotopyContinuation.jl, and then determined which of these steady states underwent a Turing- instability using the methods developed in ReactionDiffusion.jl.

Of the 1,937 unique symmetry-breaking parameter sets identified by ReactionDiffusion.jl alone, 1,740 (90%) are monostable, while 197 (10%) are multistable. We then examined whether any symmetry-breaking cases were missed because the system was multistable and only a single steady state had been analysed by ReactionDiffusion.jl. Among the 488,063 parameter sets not predicted to break symmetry by ReactionDiffusion.jl alone, we identified 15 cases in which symmetry breaking occurs at an alternative steady state. This corresponds to <0.004% of all parameter sets screened. These results demonstrate that, for the model considered here, steady-state multiplicity has a negligible effect on our conclusions regarding symmetry breaking – although, as we describe in our revised submission, multistability *may* impact the eventual pattern formed for some parameter sets.

We do not wish to include the analysis of this model in our revision since: (1) the method is restricted to specific choices of nonlinear terms and (2) more importantly, because whilst steady state multiplicity does not strongly impact the behaviour of this *specific* model, we do not wish to suggest that it will not impact the behaviour of all models *in*

© 2026. Published by The Company of Biologists under the terms of the Creative Commons Attribution License (https://creativecommons.org/licenses/by/4.0/). 

*general*. Instead, we hope that our expanded discussion of multistability in the supplement will make readers aware of this point and its associated caveats.

**In summary**, we can demonstrate that: (1) in general, our necessary conditions for symmetry breaking apply even when there are multiple steady states, and (2) for the specific models we considered, we were able to find many symmetry breaking parameter sets without proving whether we had found all steady states of each system.

5 - The software tool is useful but not sufficiently applied
The ReactionDiffusion.jl package is presented as a significant contribution, offering a flexible, open-source computational framework for simulating reaction-diffusion systems with both diffusible and nondiffusible species (lines 388-389). The package is well-implemented, as evidenced by its use in generating the pattern atlas in Figure 5 (lines 301-314), which illustrates spatial distributions of activators, inhibitors, and signaling pathway activation across various circuit types (e.g., in-phase, out-of-phase, or uniform expression). Its modular design and ability to handle hybrid systems make it a valuable tool for studying Turing-type pattern formation, particularly for systems with complex extracellular interactions. However, the manuscript applies ReactionDiffusion.jl exclusively to generic activator-inhibitor motifs, producing abstract patterns without modeling specific biological signaling pathways, such as those involved limb development (e.g., Bmp-Wnt), or dorsal-ventral patterning (e.g., BMP-Chordin, Zhu et al., 2023).

This abstract focus limits the tool's demonstrated utility, as it does not generate system-specific predictions or hypotheses that could be tested experimentally. For instance, the authors could have used the package to simulate a known developmental system, such as WNT/sFRP interactions in cortical organoids (Takata et al., 2017), and compared the resulting patterns to experimental gene expression data to validate the model's predictive power. The lack of such applications restricts the study's impact, particularly for a journal like Development, which values computational tools that advance biological understanding through concrete applications. To strengthen this aspect, the authors could apply ReactionDiffusion.jl to a specific developmental context, using biologically realistic parameters derived from literature (e.g., diffusion coefficients, reaction rates) to generate testable predictions, thereby showcasing the tool's practical value and relevance to developmental biology.

We thank the reviewer for their positive evaluation of ReactionDiffusion.jl, and also for the suggestion to include a more explicit example of using the software in a specific biological context, which we agree will improve the presentation of the methods to a wider audience.

Therefore, complementing our WNT-sFRP case study of Box 2, we provide a new supplementary figure (S7) which details how such a model was built with ReactionDiffusion.jl along with biologically informed parameter values.

Conclusion and recommendation
The manuscript presents a mathematically rigorous investigation of activator-inhibitor circuits in reaction- diffusion systems, leveraging a well-executed quasi-steady-state (QSS) reduction and a flexible, open- source simulation tool, ReactionDiffusion.jl. The derivation of the necessary condition N for pattern formation and the cataloging of eight distinct pattern-forming circuits are technically sound, and the software implementation is a valuable contribution to the field. However, the study's theoretical findings largely reaffirm established principles from prior work, such as Diego et al. (2018), Scholes et al. (2019), and Marcon et al. (2016), which similarly analyzed networks with nondiffusible signaling nodes and derived instability conditions using stability matrix partitioning.

The manuscript's biological relevance is limited by the absence of specific applications to developmental systems or comparisons with experimental data, which is critical for a journal like Development. The failure to explore nonlinear dynamics, such as multistability arising from dimerization-like interactions (e.g., ligand- inhibitor complexes), and the insufficient engagement with relevant literature further weaken its impact.

I recommend rejection in its current form but I encourage resubmission after substantial revisions to address the following:

© 2026. Published by The Company of Biologists under the terms of the Creative Commons Attribution License (https://creativecommons.org/licenses/by/4.0/).

-Clearly articulate the study's novel contributions relative to prior work by Diego et al. (2018), Scholes et al. (2019), Marcon et al. (2016), and Zhang et al. (2016), emphasizing unique insights into extracellular interaction mechanisms or circuit designs not previously explored.
-Investigate the nonlinear dynamics introduced by dimerization, such as multistability or bifurcations, through bifurcation analysis or simulations to uncover new pattern-forming mechanisms relevant to developmental biology.

-Apply the modeling framework and ReactionDiffusion.jl to a specific developmental system, such as WNT/sFRP signaling in cortical organoids (Takata et al., 2017) or BMP-Chordin in Xenopus dorsal-ventral patterning (Zhu et al., 2023), generating testable predictions or constraints validated against experimental data to enhance biological relevance.

These revisions would transform the manuscript from a methodological exercise into a conceptually significant contribution to developmental biology, aligning with Development's standards for impactful research.

We thank the reviewer for their thorough review and helpful suggestions, and are pleased to hear that they consider our results to be “technically sound” and our software to be “a valuable contribution”. We hope that the substantial changes we have made in revision better showcase how these theoretical findings and computational methods indeed do make a “conceptually significant contribution” to our understanding of symmetry breaking in vivo.

*************************************References*********************************************

Zheng, M., Mocarlo, Bin Shao, and Qi Ouyang. "Identifying network topologies that can generate Turing pattern." Journal of Theoretical Biology, 2016.

We confirm that this paper was already cited in our first submission.

**Reviewer 2:**

This paper analyses reaction-diffusion models with intercellular signalling mechanisms that build upon the classic two-component Turing models. The authors demonstrate, through a combination of formal mathematics and high-throughput numerical simulation, that the capacity for pattern formation is determined by extracellular inhibitory interactions and is largely insensitive to intracellular dynamics. By providing an open-source software pipeline (ReactionDiffusion.jl) for simulating such systems at scale, the work both clarifies the design principles underlying Turing-like patterning and offers practical tools for experimentalists and theorists.

We thank the reviewer for their positive evaluation and helpful comments below, each of which we have incorporated in our revised manuscript.

SUGGESTIONS TO AUTHORS
Overall, a very nice clear and well-written paper. In fact, the only sentence I would suggest needs an edit is line 223 where "it inhibits it" is a clumsy way to express signalling inhibits the prescribed output.

We agree, and have edited as suggested.

The only other part that could be additionally clarified is that throughout the manuscript a script form of N is used to represent the patterning conditions. However, this becomes a bold sans-serif in the figures. If the authors could match the fonts better then it would be the connection between the text and figures better.

Again, we agree, and have edited the relevant figures accordingly.

Apart from these points the only thing that the paper needs is perhaps a few more references to current theoretical literature as a number of the comments or questions raised in the introduction are either answered or clarified.

© 2026. Published by The Company of Biologists under the terms of the Creative Commons Attribution License (https://creativecommons.org/licenses/by/4.0/).

Thank you for flagging these additional references – we have referred to and cited them at points in the manuscript that we felt were most appropriate; in some cases, this was the introduction, in other cases, it was the discussion section.

- There is some discussion about whether N provides a necessary, or sufficient set of conditions

The following paper show that they aren't sufficient

```
@Article{Krause-2024-TIA,
author    = {Krause, A. L. and Gaffney, E. A. and Jewell, T. J. and Klika, V. and Walker, B. J.},
title     = {Turing instabilities are not enough to ensure pattern formation},
number    = {2},
pages     = {21},
volume    = {86},
journal   = {Bull. Math. Biol.},
publisher = {Springer},
year      = {2024},
}
```

The following paper shows that they aren't necessary

```
@Article{Brauns-2020-MassConserving,
author    = {Brauns, F. and Halatek, J. and Frey, E.},
title     = {Phase-space geometry of mass-conserving reaction-diffusion dynamics},
number    = {4},
pages     = {041036},
volume    = {10},
journal   = {Physical Review X},
publisher = {APS},
year      = {2020},
}
```

Although there is a separation of the domains, since the paper considers 1D patterns there's little mention of the effect of the domain and its boundary conditions in terms of driving pattern

```
@Article{Woolley-2022-BCC,
author    = {Woolley, T. E.},
title     = {{Boundary conditions cause different generic bifurcation structures in Turing systems}},
number    = {9},
pages     = {1--38},
volume    = {84},
file      = {:https\://link.springer.com/content/pdf/10.1007/s11538-022-01055-x.pdf?pdf=button:},
journal   = {Bull. Math. Biol.},
publisher = {Springer},
year      = {2022},
}
```

```
@Article{Woolley-2025-PFR,
author    = {Woolley, T. E.},
title     = {Pattern formation on regular polygons and circles},
number    = {1},
pages     = {1--44},
volume    = {35},
journal   = {J. Nonlinear Sci.},
publisher = {Springer},
year      = {2025},
}
```

Since you're creating a new tool, it is probably worth highlighting the recent online development of Turing simulations

```
@article{walker2023visualpde,
```

© 2026. Published by The Company of Biologists under the terms of the Creative Commons Attribution License (https://creativecommons.org/licenses/by/4.0/).

title={VisualPDE: rapid interactive simulations of partial differential equations},
author={Walker, B. J. and Townsend, A. K. and Chudasama, A. K. and Krause, A. L.},
journal={Bulletin of Mathematical Biology},
volume={85},
number={11},
pages={113},
year={2023},
publisher={Springer}
}

In the first part of the introduction the authors highlight that Turing patterns only exist in small parameter regions, but the next two papers have fully generalised the creation of Turing patterns to exist in whatever parameter region is required.

@Article{Woolley-2021-BTS,
author = {Woolley, T. E. and Krause, A. L. and Gaffney, E. A.},
title = {{Bespoke Turing Systems}},
number = {5},
pages = {1--32},
volume = {83},
journal = {Bull. Math. Biol.},
publisher = {Springer},
year = {2021},
}

@Article{Woolley-2025-Bespoke2,
author = {Woolley, T. E.},
title = {Bespoke Turing patterns with specific nonlinear properties},
doi = {10.1098/rspa.2025.0030},
eprint = {https://royalsocietypublishing.org/doi/pdf/10.1098/rspa.2025.0030},
number = {2312},
pages = {20250030},
url = {https://royalsocietypublishing.org/doi/abs/10.1098/rspa.2025.0030},
volume = {481},
abstract = {Turing patterns offer a mechanism for understanding self-organization in biological systems. However, due to their flexibility, it is a mechanism that can often be abused. Here, we construct a minimal Turing system defined by just four parameters controlling the: diffusion rate, steady state, linear dynamics and nonlinear dynamics. Using just these four parameters, we can construct a set of kinetics with a number of desirable properties. Firstly, we can turn any homogeneous steady state into a Turing unstable steady state. Secondly, we can ensure that the Turing instability appears within any chosen parameter region. Thirdly, this formulation provides an unbounded patterning parameter space with guaranteed positive solutions. Finally, using weakly nonlinear analysis, we demonstrate that if we have freedom in any two of the parameters, then we can define any required pattern transition (i.e. spots-to-stripes, or stripes-to-spots) under any given changes of one of the parameters. Thus, if a Turing system is going to be applied to understand a specific biological system and, moreover, if it is going to be used to extrapolate predictions for experimental perturbations, then our findings underscore the necessity of heavily restricting the modelling components and parameter values, since any freedom could be exploited to generate potentially contradictory predictions.},
journal = {Proc. Roy. Soc. A},
year = {2025},
}

In summary, a very nice paper that offers a new tool to analyse the applicability of Turing patterns. I hope the authors find my additional citations useful for the introduction.

**Reviewer 3:**

SUMMARY OF THE ADVANCE MADE IN THIS PAPER AND ITS POTENTIAL SIGNIFICANCE TO THE FIELD

© 2026. Published by The Company of Biologists under the terms of the Creative Commons Attribution License (https://creativecommons.org/licenses/by/4.0/). 

In this manuscript, Muzatko et al. investigate complex reaction-diffusion mechanisms with the aim to incorporate realistic intercellular signaling mechanisms, intracellular dynamics, and multi-step interactions. The authors introduce the practical ReactionDiffusion.jl software tool, which automates the analysis and simulation of reaction-diffusion systems. ReactionDiffusion.jl is easy to use, well-documented, and openly available, which will likely facilitate broad usage in the field. A key claim from using this tool along with additional mathematical analyses is that extracellular interactions largely determine the pattern-forming capacity of reaction-diffusion systems, whereas intracellular circuitry matters comparatively little and can be compressed into steady-state input-output relations. Indeed, the authors show across simulations of multiple intracellular implementations that the set of predicted parameter points overlaps strongly with the true pattern-forming set. The reduction of complex PDE systems into simplified criteria depending only on extracellular and steady-state intracellular response functions is elegant and useful. The manuscript is very well written, accessible to a broad range of readers, and acknowledges the limitations of the approach. I only have a few minor recommendations that the authors could consider to bolster the conclusions and further improve the presentation of this excellent paper.

We thank the reviewer for their positive comments and helpful suggestions to improve the manuscript presentation. We have addressed each point in turn below. We have prioritized new analyses/simulations on those comments which we consider most important to support/present the central findings of our work, and in the other cases have incorporated the suggestions by changes to the manuscript text alone.

SUGGESTIONS TO AUTHORS

1) Conceptual advance

a) The models are limited to a single activator and inhibitor and to one-dimensional domains for simulations. While the authors acknowledge this, many developmental systems exhibit geometry-dependent instabilities, anisotropic diffusion, and multi-ligand crosstalk that can qualitatively change patterning windows and robustness. It would be very useful for the readers if the authors could further extend their analyses. I suggest to at least include another common activator/inhibitor case, where an inhibitor can both act at the level of the receptor and the activator (Chen and Shen 2004).

Whilst we certainly agree with the reviewer that these factors (geometry-dependent instabilities, anisotropic diffusion, multi-ligand crosstalk, multiple roles of Lefty) would be interesting to explore, we have already surveyed a large number of activator-inhibitor models in this work and therefore consider these further extensions to be outside the scope of the current manuscript. Instead, we have highlighted each of these possibilities in the discussion section, highlighting them as future directions for inquiry.

Moreover, secreted ligands/inhibitors often play more than one role in a given system as assumed here; for example, Lefty inhibits Nodal signalling by binding to both the Nodal ligand and its obligate coreceptor (Chen and Shen, 2004)…

Future work will be required to extend our pipeline to broader classes of pattern with more realistic geometries, tissue growth, anisotropic diffusion, and external/initial asymmetries, as is often present in vivo…

b) The title includes the very general reference to "Turing-like reaction-diffusion mechanisms", but the paper exclusively focuses on activator/inhibitor systems. To support the generality implied in the title, it would be helpful if the authors could also include non-activator/inhibitor-based circuits (Marcon et al. 2016).

We acknowledge that the term “reaction-diffusion mechanism” overstates the generality of the systems we directly simulated, therefore we have decided to sharpen our title (and also relevant sections in the abstract) to better reflect this point:

**Fundamental limits on symmetry breaking by Turing-like activator-inhibitor mechanisms**

© 2026. Published by The Company of Biologists under the terms of the Creative Commons Attribution License (https://creativecommons.org/licenses/by/4.0/).

c) To support the statement "We have implemented highly-performant, adaptive time-stepping solvers that allow systems of 10+ PDEs to be simulated interactively on a standard desktop machine", the authors could show the limits of their approach for larger systems - such as an exhaustive Turing space characterization of a previously postulated 10-node network (Diego et al. 2018).

Firstly, in our revised manuscript, we have provided details of the computer setup used to simulate similar complexity PDE systems (e.g., the model in Figure 1c involves 10+ variables) demonstrating the performance of ReactionDiffusion.jl.

Secondly, as we acknowledged in our initial submission, whilst fast, our simulation pipeline is not sufficiently fast to exhaustively characterize parameter spaces for large reaction-diffusion systems, which is what motivated us to search for simplifying model reductions.

However, the reviewer raises the good point that, for linear models, an algebraic approach may be more appropriate for exhaustively characterising the Turing space. Accordingly, we have highlighted this – and associated method RDNets – in our discussion section, highlighting the advantages and disadvantages of ReactionDiffusion.jl compared to other available tools and methods:

More generally, there are instances where a topology/network-based approach may be more instructive – for example, performing fast algebraic analysis of linear models with RDNets (Marcon et al., 2016) or numerical analysis of generic network architectures (Scholes et al., 2019; Zheng et al., 2016) – but in other cases, a more explicitly mechanistic model, as we have explored here, may be more informative.

d) The authors found high empirical agreement between the necessary condition and true pattern-forming regimes, but there are acknowledged counter-examples, where the agreement is < 40%. Where agreement drops (e.g. due to a large activator/inhibitor diffusion ratio), it would be very useful if the authors could diagnose the mechanism. Even a heuristic explanation would guide users of ReactionDiffusion.jl about when to rely on the necessary condition compared to a full dispersion analysis.

We have invested significant time trying to diagnose this scenario, both numerically and analytically. On the analytical side, we have not found any approaches that simplify the computation of the sufficient conditions for symmetry breaking that are compatible with the model simplification that we used to calculate the necessary condition N (which we need to provide general results). On the numerical side, we have hypothesized several possibilities (e.g., we initially thought that the lower agreement would apply when the intracellular reaction rates were very large), but when investigating systematically across all models, we could not confirm this hypothesis. Therefore, even at the heuristic level, there is not a simple rule or guide that we can give that is general.

However, we emphasize that this is not a concern for two reasons:

1) Whilst the number of symmetry-breaking parameter sets can be lower than parameter sets satisfying N (e.g., in this case, < 40%), our numerics confirm that the symmetry breaking space is always *within* the region of parameter space satisfying N. In other words, 100% of the symmetry- breaking parameters satisfy the necessary condition N. In this sense, there is no discrepancy – the numerics confirm our proof that the necessary condition N must always hold – and therefore the limits we identify (e.g., the regulatory motifs compatible with symmetry breaking) will still apply.
2) On a practical level, for users of ReactionDiffusion.jl, we in fact do perform a "full dispersion analysis" rather than computing N, and thus any parameter sets identified by ReactionDiffusion.jl satisfy both the necessary and sufficient conditions for symmetry breaking automatically. The reason that we explicitly compared the symmetry-breaking and N-satisfying parameter sets (like in Figure 3) was to demonstrate that there were parameter regimes in which the limits defined by N were relatively tight and thus informative.

© 2026. Published by The Company of Biologists under the terms of the Creative Commons Attribution License (https://creativecommons.org/licenses/by/4.0/).

We have expanded our discussion on these points by editing the figure caption:

Example parameter regime in which the necessary condition N holds but provides a significant overestimate of the symmetry-breaking parameter space. For this model, when the diffusion ratio is small, D_I/D_A =0.01, less than 40% of the parameters satisfying N satisfy the necessary and sufficient conditions.

And corresponding reference in the main text:

We could find parameter regimes where $\mathcal{N}$ overestimates the symmetry-breaking parameter space (Figure S2E), although this required substantial differences in diffusion rates that may not be relevant *in vivo*.
Moreover, we confirmed that every system that we predicted to break symmetry via ReactionDiffusion.jl indeed satisfied the necessary condition $\mathcal{N}$.

e) As stated on page 7 of the Supplementary Information, the analysis depends on the steady-state assumption of intracellular species, which could be problematic on the time-scale of pattern formation. In systems where intracellular kinetics introduce delays, bistability, or oscillatory transients, such approximations can reshape instability loci and selected wavelengths. The empirical overlap analysis is helpful, but a more explicit treatment of when the quasi-steady approximation is justified (e.g. time-scale separation criteria) would be more compelling.

We thank the reviewer for these comments, which were also raised by Reviewer 1. This gets to the heart of what we feel is the most exciting part of our results, namely the ability to derive general yet exact conclusions from simple/reduced models that assume the quasi-steady-state approximation. We want to emphasize that our results are not approximate, but rather that they can be exactly derived from considering a reduced system in which we do assume that intracellular species reach quasi-steady-state. This was a very counterintuitive result to us, but one that has been thoroughly tested with our numerical simulations, and shown via mathematical proof. However, based on the above reviewer comments, it is clear that we had failed to accurately communicate these findings in our first submission, and therefore we have added an entire new paragraph to the main text which attempts to explain the generality of our results more explicitly:

We emphasize that these limits are not approximate nor do they rely on any separation of timescales between intracellular and extracellular components. Instead, the necessary condition N of systems with arbitrarily complex intracellular dynamics (including oscillations, time averaging, other temporal filtering) may, counterintuitively, be determined exactly without explicitly considering these dynamics (see Supplementary information). This does not imply that these dynamics will have no impact on patterning, but rather that they do not affect the necessary condition N, and thus the ability of the circuit to break symmetry. Therefore, there exist general limits on symmetry breaking that will be broadly applicable regardless of the precise intracellular feedback mechanisms - or intracellular parameters - at play (Figure 2C). This result is unexpected, since prior work highlighted the importance of considering non-diffusible species in reaction-diffusion circuits (Diego et al., 2018a; Marcon et al., 2016), but we find that a single non-diffusible component per signalling pathway is sufficient to compute N.

Moreover, we have tried to explain the mathematical intuition underpinning these results in the supplementary information.

© 2026. Published by The Company of Biologists under the terms of the Creative Commons Attribution License (https://creativecommons.org/licenses/by/4.0/).

A loose intuition underpinning this result may be gained by considering the definition of $\mathcal{N}$, which states that $|\mathbf{J} - q^2\mathbf{D}|$ must change sign twice for some positive (and finite) values of $q$ for symmetry breaking to be possible. This means that there must be two values of the wavevector, $q_1, q_2$, for which this determinant (and therefore the relevant eigenvalue) must be exactly zero (i.e., $|\mathbf{J} - q_1^2\mathbf{D}| = 0$ and $|\mathbf{J} - q_2^2\mathbf{D}| = 0$). For example, at $q = q_1$ and infinitesimally above it, the instability will thus have an eigenvalue with an infinitesimally small real part i.e., it will exhibit very slow dynamics. As $q \to q_1^+$, these dynamics will thus become infinitely slow and at some point will be slow enough that all intracellular dynamics will reach a quasi-steady state over this timescale. Now, it is important to note that this argument only applies at values of the wavevector with zero growth rates (i.e., $q_1, q_2$), and that between these values, the intracellular interactions will impact the resulting dynamics and eventual pattern formed. However, since $\mathcal{N}$ is determined by the determinant sign change (i.e., precisely by the points $q_1, q_2$), this means that the intracellular dynamics do not impact the computation of $\mathcal{N}$. Following the same arguments, we also conjecture that time delays in the intracellular compartment may also be neglected when computing $\mathcal{N}$, but we have not formally proved this or demonstrated its validity numerically.

Finally, we have included a biological case study (Box 1), in which we aim to describe the biological implications of these results.

*Example 1: Symmetry breaking without a short-range activator and long-range inhibitor*

To illustrate how $\mathcal{N}$ may be applied to understand symmetry breaking in vivo, we now consider one example activator-inhibitor model in more detail, specifically one that comprises two distinct signalling pathways, WNT and BMP, in which WNT assumes the role of the Turing-like activator (i.e., undergoes positive feedback), and BMP acts as the Turing-like inhibitor. Indeed, there is experimental support for WNT-BMP circuits being involved in symmetry breaking in a variety of tissues, for example in the developing digits (Raspopovic et al., 2014) and fingerprint ridges (Glover et al., 2023). Previous theoretical work suggested constraints on the diffusive range of these signals for symmetry breaking to occur. In classic two-component Turing models (Gierer and Meinhardt, 1972), the diffusivity of the BMP ligand must be strictly larger than the diffusivity of the WNT ligand (Figure B1A). However, it was found that adding a single intracellular component to the models alleviated the requirement for differential diffusivity (Marcon et al., 2016), but the overall diffusive range of BMP (defined by both the diffusivity and extracellular half-life, $l_{BMP} \propto \sqrt{D_{BMP}\tau_{BMP}}$) must still be higher than the overall diffusive range of WNT (Figure B1B). Based on the mathematical insights provided by $\mathcal{N}$, we now considered how these constraints might change when adding further intracellular components to the models (see Supplementary information for an expanded discussion). Initially, we incorporated just two intracellular components, one to represent WNT signal transduction (i.e., βCAT) and the other to represent BMP signal transduction (i.e., pSMAD). We predict that symmetry breaking is now possible even if WNT and BMP have identical diffusive ranges (i.e., same diffusivity and half-lives), provided there is sufficiently strong positive feedback on WNT signalling (Figure B1C). Thus, it seems that progressively adding intracellular components to the models reduces the constraints on the diffusive ranges of WNT and BMP, consistent with previous theoretical arguments (Marcon et al., 2016). One might imagine, therefore, that adding even more intracellular components would reduce further the requirements on WNT and BMP to break symmetry; indeed, a more detailed 5-component model of WNT-BMP interactions in the developing digits was reported to be highly robust to parameters (Marcon et al., 2016). However, we have shown, rather generally, that the limits derived from $\mathcal{N}$ for a WNT-BMP system with just two intracellular components (one per signalling pathway) are exactly equivalent to those derived for WNT-BMP models with any more complex intracellular scheme (Figure B1D). Therefore, whilst the requirements of differential diffusivity (Figure B1A) and differential diffusive range (Figure B1B) are not fundamental but instead a result of model oversimplification, the requirement for strong positive feedback (Figure B1C) is a general limit that must apply for all WNT-BMP systems of this type no matter how complex the intracellular circuitry is. Finally, we note that this property is not universal to all types of activator-inhibitor mechanism – for example, as outlined in the Supplementary information, a WNT-DKK circuit must always have differential diffusive range between WNT and DKK for symmetry breaking to occur.

We hope that these major edits have clarified the comments raised above.

It would also be very valuable to explore a counterexample with explicit delays, which are

© 2026. Published by The Company of Biologists under the terms of the Creative Commons Attribution License (https://creativecommons.org/licenses/by/4.0/).

well known to play crucial roles in shaping cellular dynamics and pattern formation.

We are also very much interested in time delays! However, we have a preliminary sketch proof and loose intuitive arguments to suggest that – similar to intracellular dynamics – adding time delays won't relax the requirements on N. However, we leave this as a conjecture in the supplement as we have not confirmed these results via direct simulation, and do not have a general mathematical proof – but we strongly suspect that N will not depend on including time delays. Of course, as with the intracellular dynamics, this does not mean that time delays won't impact the resulting pattern! Rather it means that we can still derive general limits that apply regardless of the time delays, and as outlined in Figure 5, we would argue that already these general limits have led us to novel and interesting conclusions.

f) The binding of A and I in equation system 6 of the Supplementary Information relates well to the law of mass action, whereas Case 2 (The inhibitor binds to the receptor) actually uses A/I as the inhibitory term, which is not easily related to law of mass action kinetics. Consistent with the motivation of the authors to describe realistic signaling systems, it would be very helpful to model Case 2 consistent with the law of mass action.

As suggested, we have explicitly considered the case of receptor competition using mass action kinetics. Interestingly, we find that the necessary condition, N, takes a very similar form as the simpler model – we outline the mathematical reason for this in the supplementary materials – and in particular has the same sign constraints on the normalized sensitivities. This more realistic implementation of Case 2 is included in supplementary materials section 3.2.4.

g) F_s is always assumed to be linear (e.g. p. 9 of the Supplementary Information). What is the outcome with non-linear versions, which are biologically more realistic?

This is an interesting point which we had previously considered but decided to leave out of our initial submission as it simplifies the algebra.
There is in fact a rather simple change to the necessary conditions when allowing nonlinear F_s terms, corresponding to multiplying the respective normalized sensitivities by the normalized sensitivity of F_s itself (when F_s is linear, the normalized sensitivity is just 1). We agree it would be helpful to include this scenario in our models, and therefore add this to the supplementary materials:

*Remark:* Above, we assumed that $F_S$ is linear. Whilst often applicable [15], this assumption is not necessary and analogous results follow even if $F_S$ is non-linear. In particular, when $F_S$ is non-linear $H_{A\to S} \neq 1$ and therefore the linearized reaction-diffusion matrix takes the form:

$$\widetilde{\mathrm{M}}(q^2) = \begin{pmatrix} -k_{+I} - 1 - q^2 & -k_{+I} & H_{S\to A}(1+k_{+I}) \\ -k_{+A} & -k_{+A} - \delta_{I/A} - D_{I/A}q^2 & H_{S\to I}(\delta_{I/A} + k_{+A}) \\ H_{A\to S}\delta_{S/A} & 0 & -\delta_{S/A} \end{pmatrix}.$$

Therefore the characteristic polynomial is changed analogously:

$$(-1)^n det(\widetilde{\mathrm{M}}(q^2)) = D_{I/A}\delta_{S/A} \cdot q^4 - \left(\delta_{S/A}\left(D_{I/A}(H_{A\to S}H_{S\to A} - 1)(1+k_{+I}) - \delta_{I/A} - k_{+A}\right)\right) \cdot q^2 + \\ -\delta_{S/A}\left((\delta_{I/A} + k_{+A} + \delta_{I/A}k_{+I})(H_{A\to S}H_{S\to A} - 1) + k_{+I}\left(H_{A\to S}H_{S\to A}k_{+A} - H_{A\to S}H_{S\to I}(\delta_{I/A} + k_{+A})\right)\right). \quad (16)$$

In particular, $H_{A\to S}$ appears always in a product with $H_{S\to A}$ or $H_{S\to I}$. Therefore, inequalities involving $H_{S\to A}$ or $H_{S\to I}$ are replaced by exactly the same inqualities but now involving the products $H_{A\to S}H_{S\to A}$ or $H_{A\to S}H_{S\to I}$. Fortunately, these terms remain interpretable via the intracellular response functions. For example, the term $H_{A\to S}H_{S\to I}$ represents the overall effect of a given concentration of extracellular ligand, $A$, on the resulting production/transcription of the inhibitor, $I$, regulated via the intracellular signal, $S$. The product term naturally arises via the chain rule that captures both the dependence of pathway activation on ligand concentration, as well as the feedback from intracellular signalling to inhibitor production.
Analogous results follow for all the other models considered, showing that the linear assumption on $F_S$ is convenient but not necessary. For simplicity, we will assume $F_S$ is linear for the remaining models considered.

h) The ratios and dimensionless groups used throughout the manuscript are insightful, but additional examples with real measured values - where possible - would be more useful for developmental biologists, also because the final pattern (wavelength etc.) depends on the

© 2026. Published by The Company of Biologists under the terms of the Creative Commons Attribution License (https://creativecommons.org/licenses/by/4.0/).

absolute values.

This is an excellent suggestion, and we have incorporated this into a new extended case study in Box 2, together with supplementary code and parameter values in Figure S7.

i) To at least provide an initial assessment of how a simple physical process affects the stability and scaling of the predicted patterns from Figure 5, the authors could introduce uniform domain growth into the existing 1D simulations within the ReactionDiffusion.jl framework. This would further bolster conclusions regarding robustness.

Growth may indeed be playing an important role in many biological tissues, however incorporating growth into our simulation framework in a robust and easy-to-use manner is beyond the scope of the current manuscript. We have instead highlighted the potential roles for growth as a limitation/future direction in the Discussion:

Future work will be required to extend our pipeline to broader classes of pattern with more realistic geometries, tissue growth, anisotropic diffusion, and external/initial asymmetries, as is often present in vivo (Grall et al., 2024; Haupaix et al., 2018; Ho et al., 2019; Raspopovic et al., 2014), and as has been achieved for simpler (2-4 component) models elsewhere (Walker et al., 2023).

2) ReactionDiffusion.jl software
a) To quantify and justify claims about computational performance in the manuscript, it would be useful to perform benchmarking of ReactionDiffusion.jl compared to other existing tools.

Firstly, we have quantified the computational performance of ReactionDiffusion.jl in our revised manuscript by providing details of simulation time for some concrete simulations as follows:

### 1.2 Performance benchmarks for ReactionDiffusion.jl

We performed benchmarks on the two main features of *ReactionDiffusion.jl*:

1. *Rapid simulation of complex reaction-diffusion PDEs:* As a concrete example, we considered the 14-species model from Figure 1C. The corresponding PDE was discretized on a one-dimensional spatial grid with 128 grid points. Simulations were initialized from the homogeneous steady-state solution with added noise and integrated until convergence to steady state. The simulation time to evaluate the full spatiotemporal dynamics up until steady state was under 200ms (excluding the compilation time which is incurred only on the first run of any Julia function).

2. *Rapid detection of Turing instabilities in reaction-diffusion PDEs:* We can routinely screen millions of parameter sets per minute on standard personal laptops/desktops. For example, for the model in which ligand and inhibitor reversibly bind (i.e., Figure 5C1 of the main text), we could screen 64 million parameter sets in 379 seconds i.e., over 10 million parameter sets per minute.

All benchmarks were run on a MacBook Pro (2024) with an Apple M4 Pro processor (12 CPU cores) and 24 GB of unified memory (RAM). All computations were performed on the CPU using all available hardware threads.

We consider it difficult to specifically benchmark ReactionDiffusion.jl compared to other existing tools, because the use-cases and functionality of ReactionDiffusion.jl are not all contained within these existing tools (e.g., simulating arbitrary nonlinear PDEs, intuitive syntax and automated analysis, Turing-instability detection via numerics). For example, a clear alternative tool to contrast with would be RDNets, but this is rather different – it is confined to linear models (a drawback compared to ReactionDiffusion.jl), but it uses computer algebra to derive analytical expressions (an advantage compared to ReactionDiffusion.jl). Given the difficulty in finding a like-for-like comparison, we have therefore decided to more broadly discuss the existing tools in the discussion section and highlight the relative pros and cons of ReactionDiffusion.jl – including suggesting scenarios where other tools may in fact be more appropriate:

[repeated from point 1c above] More generally, there are instances where a topology/network-based approach may be more instructive – for example, performing fast algebraic analysis of

© 2026. Published by The Company of Biologists under the terms of the Creative Commons Attribution License (https://creativecommons.org/licenses/by/4.0/).

linear models with RDNets (Marcon et al., 2016) or numerical analysis of generic network architectures (Scholes et al., 2019; Zheng et al., 2016) - but in other cases, a more explicitly mechanistic model, as we have explored here, may be more informative.

Moreover, throughout we have ensured that any claim about computational performance is specific to ReactionDiffusion.jl, not implicitly compared to other tools, and supported by our benchmarking results.

b) To substantiate "able to screen millions of parameter sets per minute" and aid users in planning screens, it would further be useful to provide the details of one specific setup in terms of problem size / simulation task and computer configuration.

This is an excellent suggestion and we have provided this in the supplementary materials, see above.

3) Data presentation
a) I suggest to rephrase overly strong statements in the abstract and title. For example, the title appears too general, although many important factors have not been included in the study (mechanics, delays, reaction-diffusion systems beyond activators and inhibitors etc.).

We have changed the title to reflect the restricted focus on activators and inhibitors:

Fundamental limits on symmetry breaking by Turing-like activator-inhibitor mechanisms

Similarly for the abstract:

However, whilst Turing-like activator-inhibitor models can qualitatively recapitulate patterning in silico…

… more realistic systems display qualitatively distinct behaviours compared to simple activator-inhibitor models

We have also mentioned delays in the supplementary materials (see comment above), and alluded to mechanical processes in the Discussion:

We also hope that the principles derived from purely molecular patterning systems may also be relevant to systems that combine reaction-diffusion with cellular and/or mechanical processes.

b) I suggest to adjust the abstract to better reflect the limitations of the study. For example, "to directly capture the mechanisms involved in intercellular signaling" does not seem appropriate given that modeling of some inhibition mechanisms is currently not directly derived from law of mass action kinetics.

Referring to our response above, we have now modelled the case of receptor competition using mass action kinetics, so hope that it is reasonable to keep the essence of this sentence in.

Of course, even with this modification, any model is never going to be a direct/exhaustive/complete description of the enormous complexity present in intercellular signalling mechanisms. Rather, our aspiration was to build models that more directly captured mechanism - and this did give novel predictions
- and in fact we found that some aspects of the complexity (i.e., the intracellular dynamics) could be simplified without substantially changing model predictions.
To better reflect this limitation in the abstract, we have edited the corresponding sentence as follows:

Models that ***seek*** to ***more*** directly capture the mechanisms involved in intercellular signalling

c) For the abstract, I also suggest to give more credit to previous exhaustive studies that have

© 2026. Published by The Company of Biologists under the terms of the Creative Commons Attribution License (https://creativecommons.org/licenses/by/4.0/).

analyzed multi-node Turing models with a separation of extracellular (diffusible) and intracellular (non-diffusible) compartments. This also applies to the introduction paragraph in lines 48-55.

We thank the reviewer for this suggestion, and have taken considerable steps to try to better articulate what previous studies had found, and how our manuscript builds upon these findings to make novel contributions. We have made edits to several areas of the manuscript to reflect this.

First, we have significantly expanded our discussion of previous in silico screens in the introduction. We allude to these briefly in lines 48-55 as suggested, as well as restricting our attention to activator-inhibitor schemes explicitly in the abstract. We have discussed them in detail in a later paragraph of the introduction:

High throughput, systematic analyses of 3- and 4-node networks have revealed a wide array of regulatory architectures capable of periodic patterning beyond simple 2-component Turing models (Diego et al., 2018a; Haas and Goldstein, 2021; Marcon et al., 2016; Scholes et al., 2019; Shaberi et al., 2025; Zheng et al., 2016), suggesting that symmetry breaking may involve more than just one activator and one inhibitor in vivo. These analyses reveal that many types of periodic pattern can form with a variety of phase relationships possible amongst the different model components. Moreover, a general theme that has emerged is that factors that do not diffuse between cells (e.g., transcription factors, signal transduction elements) can significantly impact the ability of the system to break symmetry (Diego et al., 2018a; Marcon et al., 2016; Scholes et al., 2019). However, given the large number of these components and their possible interactions, previous models have necessarily underestimated the intracellular complexity present in vivo, and it remains unclear what impact this simplification will have on model predictions. Moreover, whilst containing more than 2 elements, the models are still simplified network-level abstractions which approximate molecular interactions by simple mathematical expressions (e.g., linear or hill functions), rather than directly capturing the nature of developmental signalling in vivo. One strategy has been to assume sigmoidal/hill-type interactions between each component (Scholes et al., 2019; Zheng et al., 2016); this has the advantage of naturally capturing the saturating nature of many biological feedbacks, but poorly reflects more direct biochemical interactions (for example, binding of an inhibitor to an activator molecule in the extracellular space). Another approach is to instead consider linear coupling between each component, facilitating a comprehensive algebraic treatment (Diego et al., 2018a; Marcon et al., 2016). However, the assumption of linearity neglects the intrinsically nonlinear character of most biological interactions (even when considering linearized dynamics since the nonlinearities themselves dictate the allowed values of any linearized coefficients). Therefore, whilst these approximations have allowed systematic analyses of reaction-diffusion circuits in silico – which has pointed to important guiding principles – it remains unclear to what extent their simplifying assumptions will impact the applicability of model predictions in vivo.

Finally, we have also provided a concrete example where we discuss the importance of intracellular compartments in Box 1, highlighting what was known from previous studies and what novel insights are provided in this study (Box 1 copied above).

Similarly, I suggest to rephrase the introduction paragraph starting with "Rather than all behaving similarly - as implicitly assumed in simple 2-component Turing models", which has already been found by previous work published over the last 10 years.

Agreed – we have changed accordingly:

Rather than all behaving similarly – as one might naively expect

We have kept the general idea of “all behaving similarly” as a possible (naïve) expectation because “simple 2-component Turing models” are still commonly used in biological modelling papers. We therefore think it is helpful to explicitly make the point that e.g., whilst a WNT-DKK system (modelled as a simple 2-component model in Sick et al 2006) and a WNT-BMP system (modelled as a simple 2-component model in Glover et al 2023) are both activator-inhibitor, they are rather different in their properties.

© 2026. Published by The Company of Biologists under the terms of the Creative Commons Attribution License (https://creativecommons.org/licenses/by/4.0/).

d) I recommend to rephrase a few statements in the manuscript that overemphasize "realistic". The authors acknowledge that pre-patterns and more complex boundary conditions are not considered, which limits the aspect of more realistic systems (e.g. Haupaix et al. 2018, Singh et al. 2014). In addition, many known aspects crucial for signaling systems (e.g. the involvement of Type II receptors in Fig. 1B) are not captured by the current models.

As the reviewer suggests, we do acknowledge that our models are considerably more simplistic than the complexity operating *in vivo*. However, our aim is not to build a *completely realistic* model, but rather one that is *more realistic* than simpler approximations (e.g., those that assume linear/hill-function couplings between components). Interestingly, we find that it is the extracellular components that are more important to model directly – thus providing a rationale for modellers to focus their complexity/realism on the extracellular rather than the intracellular compartment.

We have tried to more clearly articulate this aim in the introduction, and ensured to avoid vague claims of “realistic” and instead qualify them as “more realistic” compared to simpler approximations, e.g.,

> Here, instead of adding more activators/inhibitors, we seek to build models of simple “classic” Turing mechanisms (i.e., 1-activator, 1-inhibitor) that more realistically capture the mechanisms of intercellular signalling than network models with linear/hill-function coupling.

In addition, to highlight this point, we have explicitly stated in the discussion some ways in which our models are not realistic – including each of the important points/references raised by the reviewer above e.g., type II receptors.

In addition, our models have so far approximated multi-subunit receptor complexes by a single limiting receptor component (Klumpe et al., 2022)…

r) Please explicitly mention in the text or figure legend what the special non-arrow connectors mean in Fig. 4 and related schematics (i.e. the effective influence of the intracellular black box).

Thank you for highlighting these omissions – for all relevant figure legends, we have added the phrase

> Lines ending in a filled circle correspond to feedbacks from intracellular signals to extracellular components; in general, these can be either activating or repressive interactions.

f) In Fig. S5, is the range really from 0-1%, or is this rather the fraction ranging from 0-100%? The legend is ambiguous in that respect and states both "fraction" and "%".

We have updated the figure legend to remove this ambiguity. The range is from 0-1%, but we note that this is compressing a very high dimensional parameter space onto just a single dimension, and the precise fraction of symmetry-breaking parameters is highly dependent on the overall size/dimension/extent of the total parameter space surveyed. We also found it helpful to look at individual parameter sets and explore locally how robust these were – as provided in the left hand column of Fig S5.

g) The code is great and user-friendly, but a few minor typos in the tutorial (e.g. switched comma and semicolon for parameters) should be corrected.

We thank the reviewer for their positive feedback on ReactionDiffusion.jl, and very much appreciate them taking the time to review our code.

We have checked again the tutorial and have ensured that the example code provided correctly reproduces all tutorial figures/results without error. In particular, we have set this up so that this check is performed automatically, since all figures/results in the extended documentation (https://hiscocklab.github.io/ReactionDiffusion.jl/stable/) are reproduced “from scratch” when the documentation is built. We confirm that the docs are currently building correctly for all

© 2026. Published by The Company of Biologists under the terms of the Creative Commons Attribution License (https://creativecommons.org/licenses/by/4.0/). 

simulation outputs so we can guarantee that the code presented does not contain typos that would alter simulation outputs or cause errors.

---

Second decision letter

MS ID#: dev.205067R1

MS TITLE: Fundamental limits on symmetry breaking by Turing-like activator-inhibitor mechanisms

AUTHORS: Daniel Muzatko, Bijoy Daga and Tom W. Hiscock

Dear Dr Hiscock,

I have now received all the referees reports on the above manuscript, and have reached a decision. The referees' comments are appended below.

The overall evaluation is positive and we would like to publish a revised manuscript in Development, provided that the referees' comments can be satisfactorily addressed. The referees recognise the effort you made to address the comments from the initial review and are satisfied with these revisions. However, there are a couple of points that require clarification before we can formally accept the paper.

Please attend to the reviewers' comments in your revised manuscript and detail them in your point-by-point response. If you do not agree with any of their criticisms or suggestions explain clearly why this is so. Please send us a point-by-point response indicating your plans for addressing the referees' comments, and we will look over this and provide further guidance.

Reviewer 1

*Advance summary and potential significance to field*

I appreciate the authors' clarification that the manuscript is now explicitly restricted to diffusion-driven instabilities, that is, the linear loss of stability of a homogeneous steady state. Framed this way, it becomes clear that the work does not address pattern formation in the nonlinear sense (e.g. multistability), but rather the existence of a Turing-type instability governed entirely by the linearized dynamics.

With this reframing, the scope of the contribution becomes much clearer. In practice, after reduction, the effective systems controlling instability remain structurally equivalent to previously studied reduced reaction-diffusion models with nondiffusible components. The central mathematical object is still a reduced Jacobian whose diffusion-driven eigenvalue crossing determines instability, as in earlier work, where the presence of immobile species changes pattern forming conditions. The classification of activator-inhibitor behaviors and the emergence of in-phase or out-of-phase relationships are all consequences of this linear framework and closely mirror results already accessible in previous studies.

In this sense, the manuscript is now showing more consistently that it does not introduce fundamentally new symmetry-breaking mechanisms, nor does it reveal qualitatively new instability classes beyond those already identified in prior studies. Rather, the main theoretical result is the ability to derived reduced models from larger realistic biochemical descriptions (this is nice and strong) and that once a minimal nondiffusible layer is included, adding further intracellular complexity does not further modify the diffusion-driven instability conditions. This is a technically solid and useful finding, which establishes robustness of the reduced description.

© 2026. Published by The Company of Biologists under the terms of the Creative Commons Attribution License (https://creativecommons.org/licenses/by/4.0/).

IMPORTANT: In this context, before publication the authors must clarify the statement in the Abstract that "Rather than resulting in minor, quantitative differences, we find that these more realistic systems display qualitatively distinct behaviours compared to simple activator-inhibitor models", which remains misleading. The results do not demonstrate qualitatively distinct dynamical behaviours in the sense of new pattern-forming regimes. Indeed, changes in phase relationships are already known from reduced network models, and the relaxation of differential diffusivity requirements follows earlier work incorporating nondiffusible components. The genuinely new qualitative result here is the saturation of intracellular effects, namely that once a single nondiffusible layer is included, further intracellular complexity no longer alters the diffusion-driven instability constraints. For clarity, this sentence could either be removed from the Abstract or replaced with a shorter formulation such as: "We show that diffusion-driven instability constraints saturate once a minimal nondiffusible layer is included."

In spite of this modification, I would to acknowledge the substantial effort invested in the revision. Beyond clarifying scope and terminology, the authors have expanded the Introduction and Discussion to better situate the work relative to prior literature, added explicit limitations regarding nonlinear dynamics, and incorporated a thoughtful treatment of multistability in the Supplementary material, including additional numerical analyses to assess false negatives. These changes significantly improve the conceptual transparency of the manuscript.

The newly added biochemical examples and Jacobian presented in the response and Supplementary Information represent a clear improvement over the original version. These help illustrate how mass-action ligand-receptor and dimerization kinetics translate into concrete Jacobian structure, rather than relying purely on abstract network couplings. This explicitly demonstrates how larger mechanistic models can be treated analytically, beyond minimal topologies where selected Jacobian entries are simply set to zero (as commonly done in earlier studies). In this sense, the manuscript does indeed go beyond prior reduced-network approaches by showing how realistic biochemical interactions naturally generate the effective linear structure, which is appreciated.

The more explicit demonstration that the necessary condition N can be computed from reduced models yet applies broadly across arbitrarily complex intracellular networks strengthens the conceptual foundation of earlier reduction-based frameworks. The ReactionDiffusion.jl framework is well implemented and clearly documented, and the addition of biologically informed simulations (including Supplementary Figure S7) together with Boxes 1 and 2 (WNT-BMP and WNT-sFRP) substantially improves the biological grounding of the work. These examples help translate the formal results into experimentally interpretable scenarios and make the framework more usable for the wider community.

One small technical comment in this context: when presenting Jacobian graphs or interaction diagrams, I would encourage the authors to avoid omitting self-negative terms (diagonal degradation or decay loops). These terms are mathematically as important as any other interaction, and are essential for diffusion-driven instability. Omitting them visually can be misleading, and this has already caused some confusion in previous literature. Including these self-loops explicitly would make the representations more consistent and conceptually clearer.

Overall, the revision presents a much clearer and more complete picture of the authors' goals and contributions. The primary advance lies in showing the insensitivity of linear diffusion-driven instability to intracellular complexity, together with a useful computational framework, explicit biochemical modeling, careful engagement with nonlinear caveats, and illustrative biological examples, all of which significantly strengthen the manuscript.

Reviewer 2

*Advance summary and potential significance to field*

This paper gives a framework for when reaction-diffusion signalling networks can break symmetry. They classify distinct extracellular inhibitor mechanisms and back these up with an open computational workflow for dispersion and parameter exploration. The potential significance is that

© 2026. Published by The Company of Biologists under the terms of the Creative Commons Attribution License (https://creativecommons.org/licenses/by/4.0/). 

it explains why many plausible networks don't self organise, and it helps the field prioritise mechanisms and perturbations when designing or interpreting patterning experiments.

*Comments for the author*

All of my comments have been covered by the authors. I just have two further minor comments:

There is a bit of confusion in the text. Namely, it says first they say that " can predict whether a system can initially break symmetry but does not predict what patterns will finally self-organize". Later they claim that N is "sufficient to predict the possible wavelengths of pattern that can form". So, is N just the initial bifurcation criteria, or the full dispersion relation?

Box 2 is a good addition, but it would help to add one or two explicit, falsifiable perturbation style predictions that follow directly from the mechanism.

Reviewer 3

*Advance summary and potential significance to field*

In their revised manuscript, the authors have appropriately addressed all reviewers' comments. For the new text block "Another approach is to instead consider linear coupling between each component, facilitating a comprehensive algebraic treatment (Diego et al., 2018a; Marcon et al., 2016). However, the assumption of linearity neglects the intrinsically nonlinear character of most biological interactions (even when considering linearized dynamics since the nonlinearities themselves dictate the allowed values of any linearized coefficients). Therefore, whilst these approximations have allowed systematic analyses of reaction-diffusion circuits in silico - which has pointed to important guiding principles - it remains unclear to what extent their simplifying assumptions will impact the applicability of model predictions in vivo", the authors could consider mentioning that - as previously shown by Marcon et al., 2016 - results from linear models can be translated into nonlinear dynamical systems.

---

**Second revision**

Author response to reviewers' comments

Our response

Quoted text from revised manuscript

**Reviewer 1:**

We are delighted with the reviewer's positive evaluation of our revision, and thank them once again for their helpful feedback on our original manuscript. We agree that their suggestions have helped us to provide "a much clearer and more complete picture of the authors' goals and contributions" as well as "translate the formal results into experimentally interpretable scenarios and make the framework more usable for the wider community." Thank you!

There are two remaining suggestions in the comments below, which we respond to inline.

I appreciate the authors' clarification that the manuscript is now explicitly restricted to diffusion-driven instabilities, that is, the linear loss of stability of a homogeneous steady state. Framed this way, it becomes clear that the work does not address pattern formation in the nonlinear sense (e.g. multistability), but rather the existence of a Turing-type instability governed entirely by the linearized dynamics.

© 2026. Published by The Company of Biologists under the terms of the Creative Commons Attribution License (https://creativecommons.org/licenses/by/4.0/).

With this reframing, the scope of the contribution becomes much clearer. In practice, after reduction, the effective systems controlling instability remain structurally equivalent to previously studied reduced reaction-diffusion models with nondiffusible components. The central mathematical object is still a reduced Jacobian whose diffusion-driven eigenvalue crossing determines instability, as in earlier work, where the presence of immobile species changes pattern forming conditions. The classification of activator-inhibitor behaviors and the emergence of in-phase or out-of-phase relationships are all consequences of this linear framework and closely mirror results already accessible in previous studies.

In this sense, the manuscript is now showing more consistently that it does not introduce fundamentally new symmetry-breaking mechanisms, nor does it reveal qualitatively new instability classes beyond those already identified in prior studies. Rather, the main theoretical result is the ability to derived reduced models from larger realistic biochemical descriptions (this is nice and strong) and that once a minimal nondiffusible layer is included, adding further intracellular complexity does not further modify the diffusion-driven instability conditions. This is a technically solid and useful finding, which establishes robustness of the reduced description.

IMPORTANT: In this context, before publication the authors must clarify the statement in the Abstract that "Rather than resulting in minor, quantitative differences, we find that these more realistic systems display qualitatively distinct behaviours compared to simple activator-inhibitor models", which remains misleading. The results do not demonstrate qualitatively distinct dynamical behaviours in the sense of new pattern- forming regimes. Indeed, changes in phase relationships are already known from reduced network models, and the relaxation of differential diffusivity requirements follows earlier work incorporating nondiffusible components. The genuinely new qualitative result here is the saturation of intracellular effects, namely that once a single nondiffusible layer is included, further intracellular complexity no longer alters the diffusion- driven instability constraints. For clarity, this sentence could either be removed from the Abstract or replaced with a shorter formulation such as: "We show that diffusion-driven instability constraints saturate once a minimal nondiffusible layer is included."

We thank the reviewer for drawing our attention to this point. The aim of this sentence was not to imply that we had found distinct dynamical behaviours in the mathematical sense, but rather that different classes of activator-inhibitor model (e.g., WNT-DKK vs WNT-BMP vs WNT-sFRP) would be associated with rather different regulatory circuits and thus expected expression patterns (i.e., the qualitative differences shown in Figure 4).

However, we acknowledge that this sentence was not precise enough to convey this point accurately. Therefore we have taken the reviewer's suggestion and removed it from the abstract.

In spite of this modification, I would to acknowledge the substantial effort invested in the revision. Beyond clarifying scope and terminology, the authors have expanded the Introduction and Discussion to better situate the work relative to prior literature, added explicit limitations regarding nonlinear dynamics, and incorporated a thoughtful treatment of multistability in the Supplementary material, including additional numerical analyses to assess false negatives. These changes significantly improve the conceptual transparency of the manuscript.

The newly added biochemical examples and Jacobian presented in the response and Supplementary Information represent a clear improvement over the original version. These help illustrate how mass-action ligand-receptor and dimerization kinetics translate into concrete Jacobian structure, rather than relying purely on abstract network couplings. This explicitly demonstrates how larger mechanistic models can be treated analytically, beyond minimal topologies where selected Jacobian entries are simply set to zero (as commonly done in earlier studies). In this sense, the manuscript does indeed go beyond prior reduced- network approaches by showing how realistic biochemical interactions naturally generate the effective linear structure, which is appreciated.

The more explicit demonstration that the necessary condition N can be computed from reduced models yet applies broadly across arbitrarily complex intracellular networks strengthens the conceptual foundation of earlier reduction-based frameworks. The ReactionDiffusion.jl framework

© 2026. Published by The Company of Biologists under the terms of the Creative Commons Attribution License (https://creativecommons.org/licenses/by/4.0/).

is well implemented and clearly documented, and the addition of biologically informed simulations (including Supplementary Figure S7) together with Boxes 1 and 2 (WNT-BMP and WNT-sFRP) substantially improves the biological grounding of the work. These examples help translate the formal results into experimentally interpretable scenarios and make the framework more usable for the wider community.

One small technical comment in this context: when presenting Jacobian graphs or interaction diagrams, I would encourage the authors to avoid omitting self-negative terms (diagonal degradation or decay loops). These terms are mathematically as important as any other interaction, and are essential for diffusion-driven instability. Omitting them visually can be misleading, and this has already caused some confusion in previous literature. Including these self-loops explicitly would make the representations more consistent and conceptually clearer.

We acknowledge this point, and in our previous response to reviewers, we had included a Jacobian graph that omitted the self-loops – we provide the amended version below with decay loops included for completeness.

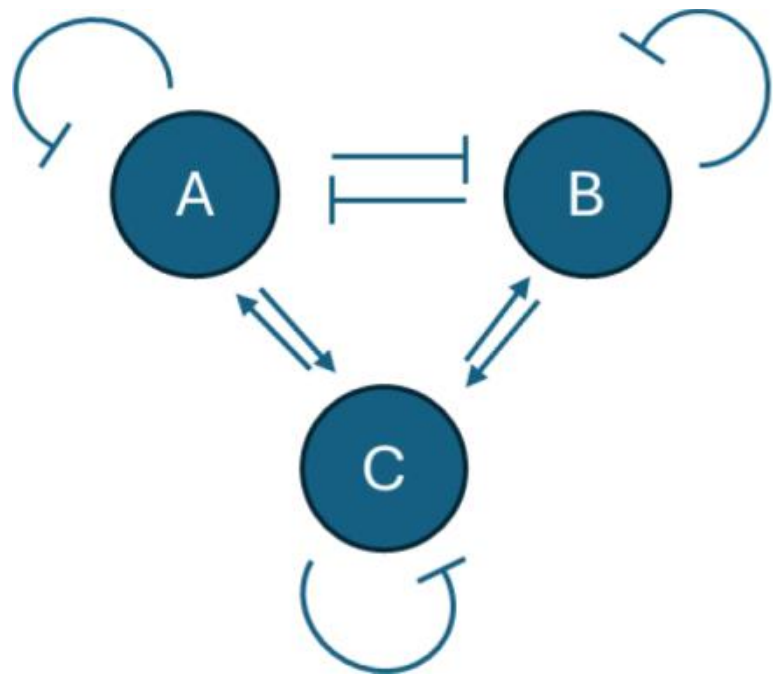


We note that in the manuscript and supplementary itself, we have not used any Jacobian graphs and therefore have not modified any of the figures.

(The schematics in the main text do not represent Jacobians, they represent biochemical/regulatory interactions. E.g., in Figure 4C, we schematize complex formation via dotted lines. Our algebraic analysis in the supplement provides details of the corresponding Jacobian structure for each model).

Overall, the revision presents a much clearer and more complete picture of the authors' goals and contributions. The primary advance lies in showing the insensitivity of linear diffusion-driven instability to intracellular complexity, together with a useful computational framework, explicit biochemical modeling, careful engagement with nonlinear caveats, and illustrative biological examples, all of which significantly strengthen the manuscript.

**Reviewer 2:**

We thank the reviewer for their comments and respond inline below.

SUMMARY OF THE ADVANCE MADE IN THIS PAPER AND ITS POTENTIAL SIGNIFICANCE TO THE FIELD

This paper gives a framework for when reaction-diffusion signalling networks can break symmetry. They classify distinct extracellular inhibitor mechanisms and back these up with an open computational workflow for dispersion and parameter exploration. The potential significance is that it explains why many plausible networks don't self organise, and it helps the field prioritise mechanisms and perturbations when designing or interpreting patterning experiments.

SUGGESTIONS TO AUTHORS

All of my comments have been covered by the authors. I just have two further minor comments:

© 2026. Published by The Company of Biologists under the terms of the Creative Commons Attribution License (https://creativecommons.org/licenses/by/4.0/).

There is a bit of confusion in the text. Namely, it says first they say that " can predict whether a system can initially break symmetry but does not predict what patterns will finally self-organize". Later they claim that N is "sufficient to predict the possible wavelengths of pattern that can form". So, is N just the initial bifurcation criteria, or the full dispersion relation?

Thank you for pointing out this imprecision in our writing.

Firstly, our approach is indeed just applicable to the initial symmetry breaking criterion and cannot "predict what patterns will finally self-organize". Therefore instead of saying "pattern that can form" we should have said "pattern that can break symmetry".

Secondly, you are correct, as we currently define it, N does not explicitly state the possible unstable wavelengths. However, as detailed in Supplementary information section 2.2, our calculation of N does implicitly consider the wavelengths (equivalently, wavenumbers q) at which the determinant of the linearized reaction-diffusion matrix changes sign. Therefore, if a Turing-instability occurs (i.e., if, in addition to N, the sufficient conditions also hold), then the determinant sign change points will correspond exactly to the wavelengths at which an eigenvalue transitions from negative to positive real part (or vice versa) i.e., they place lower and upper limits on pattern wavelengths that can break symmetry from an initially homogeneous state. To correct this, instead of saying "$\mathcal{N}$ is also sufficient to predict the possible wavelengths…", we should have said "this approach is also sufficient to predict the possible wavelengths…".

Accordingly, we have changed the sentence in the main text:

> Moreover, we show that ~~N~~ this approach is also sufficient to predict the possible wavelengths of pattern that can ~~form~~ break symmetry

Box 2 is a good addition, but it would help to add one or two explicit, falsifiable perturbation style predictions that follow directly from the mechanism.

Whilst we acknowledge this suggestion, we consider it to be outside of the scope of our current manuscript, which is focused on general approaches/principles rather than this specific example. Box 2 is intended as an example to illustrate how our findings/methods (both theoretical and computational) may be applied to concrete biological scenarios and hypotheses.

We have amended the final sentence slightly to highlight that further work – not just experimental studies – would be needed to evaluate the applicability of this model in vivo:

> Together, our results identify WNT-sFRP as a novel circuit that can break symmetry in silico; further work will be required to evaluate to what extent it is involved in symmetry breaking in vivo.

**Reviewer 3:**

We are pleased that the reviewer is satisfied with our revisions, and address their specific comment inline below.

In their revised manuscript, the authors have appropriately addressed all reviewers' comments.

For the new text block "Another approach is to instead consider linear coupling between each component, facilitating a comprehensive algebraic treatment (Diego et al., 2018a; Marcon et al., 2016). However, the assumption of linearity neglects the intrinsically nonlinear character of most biological interactions (even when considering linearized dynamics since the nonlinearities themselves dictate the allowed values of any linearized coefficients). Therefore, whilst these approximations have allowed systematic analyses of reaction-diffusion circuits in silico - which has pointed to important guiding principles - it remains unclear to what extent their simplifying assumptions will impact the applicability of model predictions in vivo", the authors could consider mentioning that - as previously shown by Marcon et al., 2016 - results from linear models can be translated into nonlinear dynamical systems.

© 2026. Published by The Company of Biologists under the terms of the Creative Commons Attribution License (https://creativecommons.org/licenses/by/4.0/).

We thank the reviewer for this suggestion, and have modified the main text accordingly:

> Another approach is to instead consider linear coupling between each component, facilitating a comprehensive algebraic treatment (Diego et al., 2018a; Marcon et al., 2016). Whilst linearized models can predict Turing instabilities even in complex nonlinear systems, a limitation of starting with linear models directly is that the coupling coefficients are allowed to freely vary, whereas their magnitudes are in fact determined by the nonlinear system itself.

---

Third decision letter

MS ID#: dev.205067R2

MS TITLE: Fundamental limits on symmetry breaking by Turing-like activator-inhibitor mechanisms

AUTHORS: Daniel Muzatko, Bijoy Daga and Tom W. Hiscock

Dear Dr Hiscock,

I am happy to tell you that your manuscript has been accepted for publication in Development, pending our standard publication integrity checks.

© 2026. Published by The Company of Biologists under the terms of the Creative Commons Attribution License (https://creativecommons.org/licenses/by/4.0/).
